# Glycine receptors expression in rat spinal cord and dorsal root ganglion in prostaglandin E2 intrathecal injection models

**DOI:** 10.1186/s12868-018-0470-8

**Published:** 2018-11-09

**Authors:** Hung-Chen Wang, Kuang-I Cheng, Pei-Ru Chen, Kuang-Yi Tseng, Aij-Lie Kwan, Lin-Li Chang

**Affiliations:** 1 Department of Neurosurgery, Kaohsiung Chang Gung Memorial Hospital, Chang Gung University College of Medicine, Kaohsiung, Taiwan; 2 Department of Anesthesiology, Faculty of Medicine, College of Medicine, Kaohsiung Medical University, Kaohsiung, Taiwan; 3 Graduate Institute of Medicine, College of Medicine, Kaohsiung Medical University, Kaohsiung, Taiwan; 4 Department of Neurosurgery, Faculty of Medicine, College of Medicine, Kaohsiung Medical University, Kaohsiung, Taiwan; 5 Department of Microbiology and Immunology, Faculty of Medicine, College of Medicine, Kaohsiung Medical University, 100, Shih-Chuan 1st Road, Kaohsiung, 80708 Taiwan; 6 Graduate Institute of Clinical Medicine, College of Medicine, Kaohsiung Medical University, Kaohsiung, Taiwan; 7 Center for Infectious Disease and Cancer Research, Kaohsiung Medical University, Kaohsiung, Taiwan; 8 Department of Medical Research, Kaohsiung Medical University Hospital, Kaohsiung, Taiwan

**Keywords:** Glycine receptors, Spinal cord dorsal horn, Dorsal root ganglion, Prostaglandin E2, Inflammatory pain

## Abstract

**Background:**

Glycine receptors (GlyRs) are involved in the development of spinal pain sensitization. The GlyRα3 subunit has recently emerged as a key factor in inflammatory pain pathways in the spinal cord dorsal horn (DH). Our study is to identify the extent of location and cell types expressing different GlyR subunits in spinal cord and dorsal root ganglion (DRGs). To tease out the possible actions of GlyRs on pain transmission, we investigate the effects produced by GlyRs on acute inflammatory pain by behavioral testing using prostaglandin E2 (PGE2) intrathecal injection models. Furthermore, we investigate the changes of GlyR expression in DRGs and spinal cord in rats after the induction of acute inflammatory pain.

**Results:**

Compared to the vehicle administration, the PGE2 intrathecal injection model produced significantly higher hyperalgesia, which started 3 h after PGE2 injection and lasted more than 5 h. PGE2 intrathecal injection significantly decreased GlyRα1 and GlyRα3 protein expressions in the L5 DH at 1 h and lasted to 5 h, and similar results were observed in the L5 DRG at 5 h. Confocal microscopic images showed the co-existence of punctate gephyrin and GlyRα3 immunoreactivity (IR) throughout the gray matter of the spinal cord, mainly in DH laminae I–III neurons and in ventral horn neurons. It also showed the co-existence of punctate gephyrin and GlyRα3 IR in DRG neurons.

**Conclusions:**

In this study, PGE2 intrathecal injection significantly decreased protein expression of gephyrin, GlyRα1 and GlyRα3 in spinal cord DH and DRG. The gephyrin and GlyRα3 were localized on neuron cells both in the DH and DRG.

**Electronic supplementary material:**

The online version of this article (10.1186/s12868-018-0470-8) contains supplementary material, which is available to authorized users.

## Background

Glycine receptors (GlyRs) are pentameric proteins belonging to the Cys-loop family of ligand-gated ion channels [1]. In rats, GlyRs consists of four different subunit families, α1-3 and β subunits, consisting of homomeric receptors that contains a single α-subunit or heteromeric receptors that contains α-and β-subunits [1]. Subsequent reports show that GlyRs are expressed in neurons in the brain, spinal cord and other regions of the mammalian central nervous system [2, 3].

Recent findings showed the involvement of GlyRs in the development of spinal pain sensitization [4]. Although the GlyRα3 subunit has recently become a key factor in inflammatory pain pathways in the dorsal horn of the spinal cord [5], The completeness of the position and the identification of the cell types expressing different GlyR subunits are not clear. Therefore, more quantitative approaches are necessary to uncover the role of GlyRs and their changes in expression profile in inflammatory pain. Quantitative methods allow the comparison of GlyRs activity at different times following inflammation.

In the present study, to tease out the possible actions of GlyRs on pain transmission, we will investigate the effects produced by GlyRs on acute inflammatory pain by behavioral testing of rats using prostaglandin E2 (PGE2) intrathecal injection models. Furthermore, we will investigate the GlyRs expression changes in the dorsal root ganglion (DRGs) and spinal cord in rats after the induction of acute inflammatory pain.

## Materials and methods

### Drugs

The prostaglandin E2 (EMD Millipore Corp., MA, USA) was dissolved in 99.9% ethanol at a concentration of 1 mg/ml. Different PGE2 dose intrathecal injection has been reported in mice [5, 6]. After converting the dose from mouse to rat [7], we used a concentration of PGE2 intrathecal injection of 625 ng/25 ul (equals to 25 mg/ml).

### Animals and experimental groups

Ninety male Sprague–Dawley rats (250–300 g, 8 weeks old) were purchased from BioLASCO Taiwan Co. (Taipei, Taiwan). The rats were housed in plastic cages at room temperature in a 12-h light-and-dark cycle, with free access to food and water. The rats were kept at least 7 days under these conditions before the study. The rats were divided into three groups: a sham (vehicle control) group; a 1 h after PGE2 intrathecal injection group, and a 5 h after PGE2 intrathecal injection group. The vehicle used in the sham group is the solvent of PGE2, 99.9% ethanol. There are 48 rats in sham group, 36 rats in 1 h after PGE2 intrathecal injection group and 44 rats in 5 h after PGE2 intrathecal injection group. In sham group, 24 rats sacrificed at 1 h and 24 rats sacrificed at 5 h after vehicle intrathecal injection. In 1 h after PGE2 intrathecal injection group, 12 rats used for behavior testing, 12 rats used for Western Blotting (6 used for GlyRα1 and 6 used for GlyRα3) and 12 rats used for Immunofluorescence (6 used for GlyRα1 and 6 used for GlyRα3). In 5 h after PGE2 intrathecal injection group, 20 rats used for behavior testing, 12 rats used for Western Blotting (6 used for GlyRα1 and 6 used for GlyRα3) and 12 rats used for Immunofluorescence (6 used for GlyRα1 and 6 used for GlyRα3). The Kaohsiung Institutional Animal Care and Use Committee approved all of the experimental procedures (Approval No. 102157).

### Intrathecal injection of prostaglandin E2

All surgical procedures were performed under isoflurane/O_2_ anesthesia. Using a modification of the intrathecal injection technique described by De la Calle and Paino [8], rats were placed in the prone position and a 2 cm longitudinal skin incision was made on the midline just above the L5 and L6 spinal process. The L5/L6 interspinous ligaments were incised, and half of the anterior L6 spinal process removed, allowing direct visualization of the L5/6 ligamentum flavum. A 30 gauge needle was inserted between the ligamentum flavum at an angle of 15°–30° horizontal to the subarachnoid space of the cauda equina. A P-10 tube was connected to a 50 μl Hamilton syringe with a 30 gauge needle (Hamilton, Reno, NV, USA) and inserted into the subarachnoid space of the cauda equina. Thereupon 25 μl of PGE2 (625 ng) was administered by intrathecal injection through the L4–L5 intervertebral space. Tail flick was a sign to identify successful intrathecal injection. After withdrawing the needle, a microscope was used to checked to ensure no fluid was leaking out. The wound was approximated with surgical sutures. The animals were placed in a recovery cage to wake up and monitored until they resumed normal activity. All the procedures were performed in institutional laboratory animal center.

### Behavioral testing

#### Hyperalgesia (noxious heat stimuli)

The latency of foot withdrawal from noxious heat stimuli was measured using the method described previously [9]. Briefly, an infrared light beam emitted from a moveable light box was projected through a hole (2 × 5 mm) to heat the glass plate under one hind paw (Ugo Basile Model 7370, Italy). Abrupt lifting, withdrawal, licking of the hind paw, or guarding posture was considered a positive response. A photocell was used to automatically turn off the light beam when the rat lifted its paw.

The time from application of the light beam to the lifting of the hind paw was recorded and defined as foot withdrawal latency. Measurements were performed at 5-min intervals and repeated five times on each hind paw, alternating between the two paws. The results were expressed as mean ± standard deviation of the 50% withdrawal threshold.

### Immunofluorescence

As in a previous study [10], the rats were sacrificed by anaesthetized with 60 mg/kg thiopentone and perfused with 0.9% saline followed by 4% paraformaldehyde in a 0.1 mol/L phosphate buffer (pH 7.4). The L5 DRG and spinal cords were removed. The dissected tissues were then fixed in 4% (w/v) paraformaldehyde and then saturated in 10–30% (w/v) sucrose in 0.02 mol/L PBS (pH 7.4). After embedding the tissues in optimal cutting temperature (OCT) compound, L5 DRGs (10 µm) and L5 spinal cords (16 µm) were prepared for immunostaining.

### Expressions of GlyRα1, GlyRα3, gephyrin, and NeuN in DRG and spinal cord

By triple immunofluorescence labelling, OCT sections were incubated for 24 h at 4 °C with the combination of three primary antibodies: goat anti-GlyRα3 polyclonal antibody (1:50, SC-17282, Santa Cruz Biotechnology, Santa Cruz, CA, USA), rabbit anti-GlyRα1 polyclonal antibody (1:50, 146,003, Synaptic Systems, Germany), or mouse anti-gephyirn monoclonal antibody (1:200, 147,021, Synaptic Systems, Germany) or chicken anti-NeuN polyclonal antibody (NeuN is neuronal nuclear protein and it is specific for neurons) (1:200, ABN91, EMD Millipore Corp., MA, USA).

These incubations were followed by incubation with secondary antibodies DyLight 405-conjugated Affinity Pure goat anti-chicken IgY, Alexa Fluor488-conjugated Affinity Pure donkey anti-goat IgG, Cy3 conjugated goat anti-mouse IgG antibody (1:200, Jackson Immuno Research, West Grove, PA, USA), or Cy5 conjugated donkey anti-mouse IgG antibody (1:200, Jackson Immuno Research, West Grove, PA, USA).

The immunoreactivity (IR) of each section was examined. The images were captured using a Zeiss LSM 700 Confocal Microscope (Zeiss, Jena, Germany).

### Western blotting

As in a previous study [10], the L5 DRG were removed to evaluate the GlyRα1, GlyRα3 and gephyrin expression in the L5 DRG and L5 spinal cord. First, the tissues were homogenized in RIPA lysis buffer [50 mmol/L Tris (pH 7.4), 150 mmol/L NaCl, 1 mmol/L EDTA, 0.1% (w/v) sodium dodecyl sulphate (SDS), 1% (v/v) NP-40, 0.5% (w/v) sodium deoxycholate] containing a complete protease inhibitor mixture (Roche Diagnostics GmbH, Mannheim, Germany).

Protein lysate (15 μg) from each sample was electrophoretically placed in 8% SDS–polyacrylamide gels and transferred onto polyvinylidene fluoride membranes (PVDF, Millipore, Bedford, MA, USA). The membranes were firstly blocked with 5% milk in phosphate-buffered saline (PBS) with 0.1% Tween-20 for 1 h at room temperature, and then probed overnight at 4 °C with Rabbit anti-GlyRα1 Polyclonal Antibody (1:1000, AGR-001, Alomone Labs, Israel), Rabbit anti-gephyrin Polyclonal Antibody (1:2000, AIP-005, Alomone Labs, Israel), goat Anti-GlyRα3 polyclonal Antibody (1:500, SC-17282, Santa Cruz Biotechnology, Santa Cruz, CA, USA), or mouse anti-actin monoclonal antibody (1:10,000, MAB1501, Indianapolis, IN, USA) primary antibody to detect the expression of GlyRα1, GlyRα3 and gephyrin in the DRG and spinal cord. This was followed by reaction with a horseradish peroxidase-conjugated secondary antibody (EMD Millipore Corp., MA, USA).

The intensity of each band was visualized by ECL Western blotting detection reagents (EMD Millipore Corp., MA, USA). Each protein expression was internally normalized using β-actin, while the expression level was normalized against the expression level of each protein in sham control rats.

### Statistical analysis

Group comparisons for behavioral responses were performed using the Mann–Whitney *U*-test. Multiple comparisons of time-dependent differences of GlyRα1, GlyRα3 and gephyrin expression in Western blots were determined by analysis of variance (ANOVA), followed by the least significant difference test for multiple post hoc analyses. The SPSS 18.0 (SPSS Inc., Chicago, IL, USA) software was used for all statistical analyses. Statistical significance was set at *p < 0.05, **p < 0.01, and ***p < 0.001.

## Results

This study involved 90 rats. After surgery, none of the animals revealed autotomy and none exhibited permanent ventroflexion or dragging of the hind paw during forward movement.

### Intrathecal injection with PGE2 induced significant hyperalgesia compared to vehicle administration

Purpose: To see the effect of PGE2 intrathecal injection produced hyperalgesia on the hind paw.

**Fig. 1:**
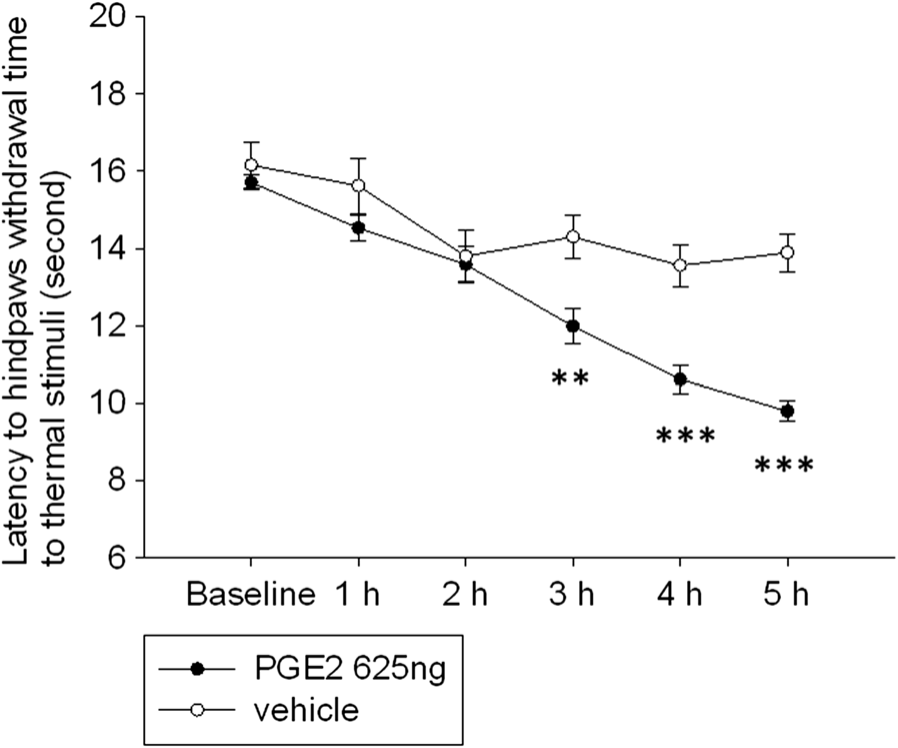
Intrathecal injection with PGE2 induced hyperalgesic responses in rats. In the PGE2 group, the hyperalgesic response was significantly different at 3 h post PGE2 injection compared with vehicle injection and lasted to 5 h. Significant differences of behavior hypersensitivity were as indicated. Mann–Whitney *U*-test, Error bars represent SE. **p < 0.01, and ***p < 0.001 (between the PGE2 and vehicle groups); h, hour

The PGE2 intrathecal injection model produced significant hyperalgesia on the hind paw as compared to vehicle treated control at 3 h and lasted more than 5 h after PGE2 intrathecal injection (Fig. 1; Additional file 1).

### Intrathecal injection with PGE2 significantly decreased gephyrin, GlyRα1 and GlyRα3 protein expressions in DH

Purpose: To see the effect of PGE2 intrathecal injection on gephyrin, GlyRα1 and GlyRα3 protein expressions in DH.

**Fig. 2:**
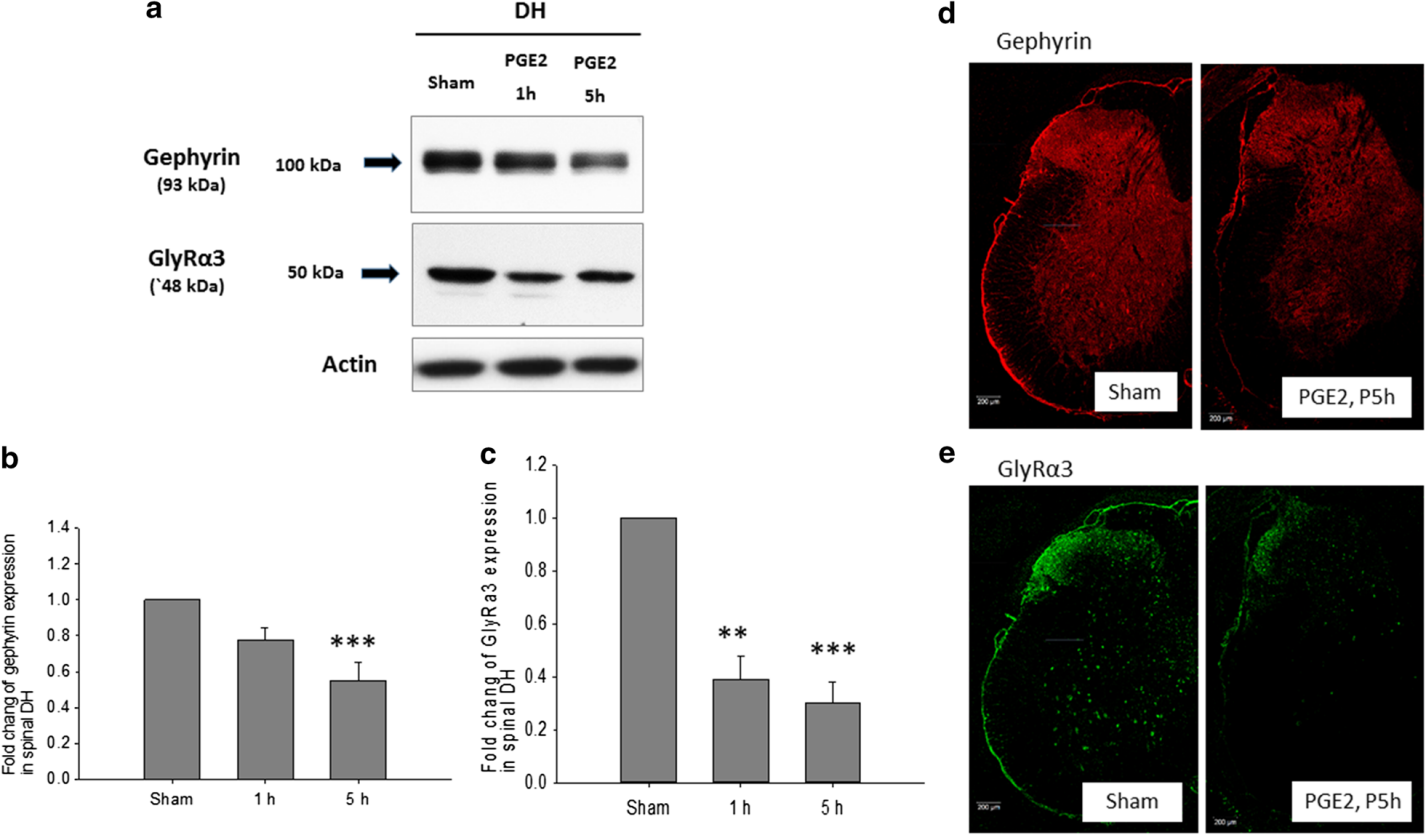
**a** Western blot and quantitative analysis of **b** gephyrin, **c** GlyRα3 expressions in L5 spinal cord dorsal horn at 1 h and 5 h after PGE2 intrathecal injection. The GlyRα3 protein expressions were significantly decreased in the PGE2 group at 1 h and lasted up to 5 h. The gephyrin expression decreased significantly at 5 h after PGE2 injection. **d** Confocal microscopic imaging showed expression of gephyrin throughout the gray matter of spinal cord. **e** Expression of GlyRα3 was mainly in the superficial layer of dorsal horn and in the ventral horn. Each group had n = 3–6 rats. Scale Bars: 200 μm; **p < 0.01, and ***p < 0.001, One-way ANOVA, followed by the LSD test

**Fig. 3:**
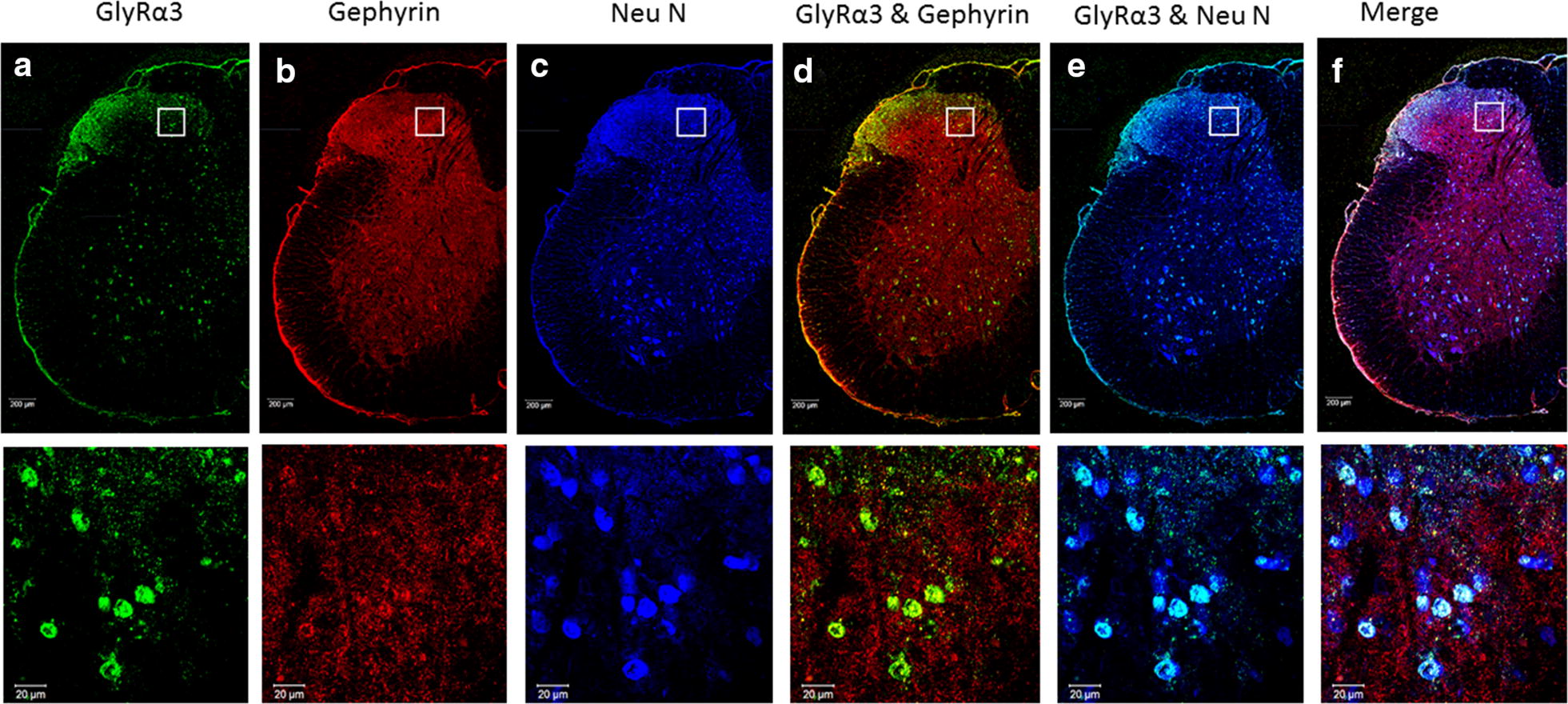
Triple immunofluorescence staining showing GlyRα3, Gephyrin and NeuN co-localization in the L5 spinal cord. Positive for GlyRα3 are shown in green (**a**), positive for Gephyrin are shown in red (**b**) and positive for NeuN are shown in blue (**c**). Double-labelled images of GlyRα3 and Gephyrin (**d**), GlyRα3 and NeuN (**e**) are indicated. **f** The merged image demonstrates co-localization of GlyRα3, Gephyrin in Neurons. Scale bars = 200 µm, 20 µm

The PGE2 intrathecal injection significantly decreased GlyRα1 (Additional file 2: Fig. 1) and GlyRα3 protein expressions in the L5 DH at 1 h and lasted to 5 h (Fig. 2a, c, and e; Additional file 3). The gephyrin expression decreased significantly in the L5 DH at 5 h (Fig. 2a, b and d; Additional file 3). Confocal microscopic images showed the co-existence of punctate gephyrin and GlyRα3 immunoreactivity (IR) throughout the gray matter of the spinal cord, mostly in DH laminae I–III neurons and in ventral horn neurons (Fig. 3).

### Intrathecal injection with PGE2 significantly decreased gephyrin, GlyRα1 and GlyRα3 protein expressions in DRG

Purpose: To see the effect of PGE2 intrathecal injection on gephyrin, GlyRα1 and GlyRα3 protein expressions in DRG.

**Fig. 4:**
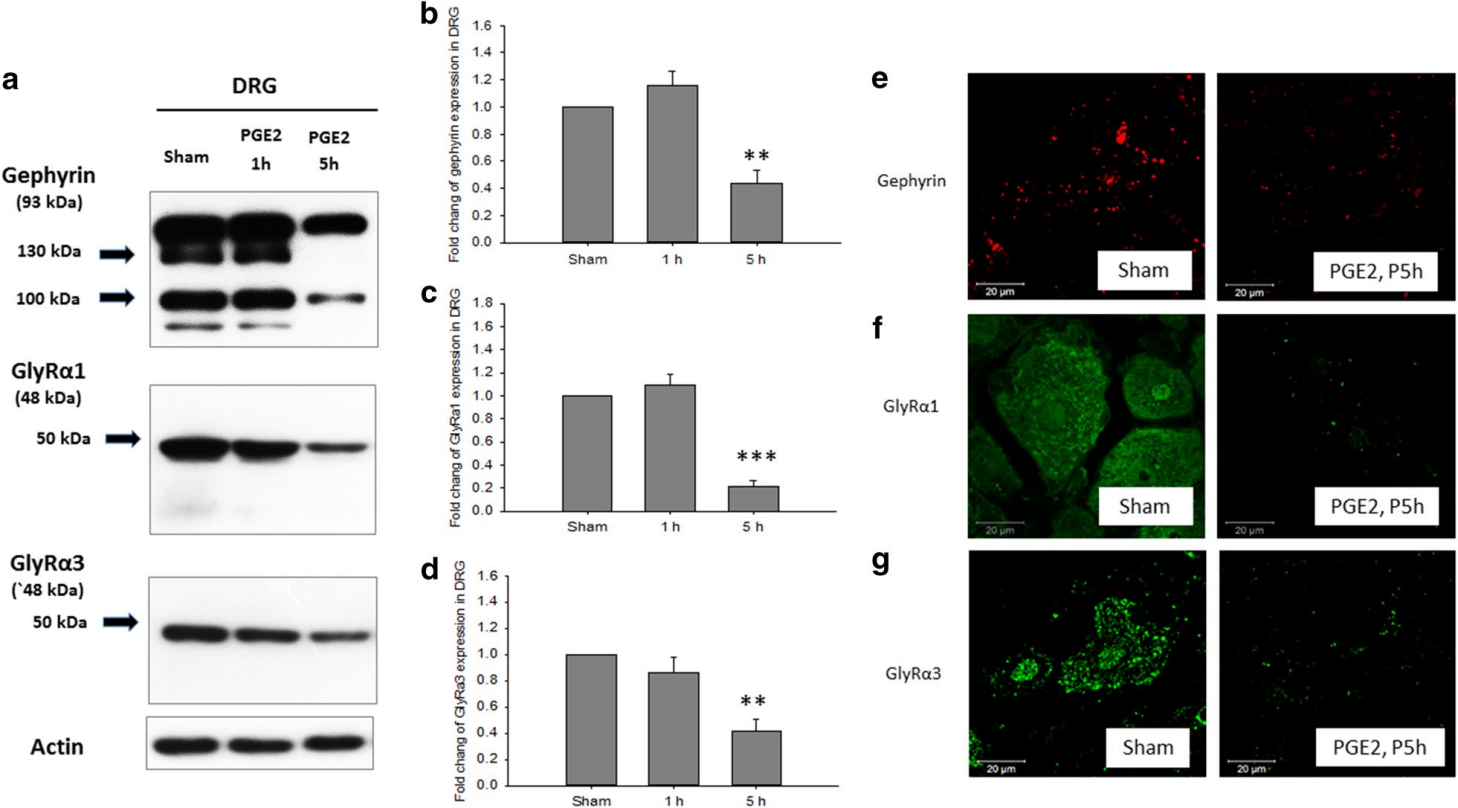
**a** Western blot and quantitative analysis of **b** gephyrin, **c** GlyRα1, and **d** GlyRα3 protein expressions in L5 DRG at 1 h and 5 h after PGE2 intrathecal injection. The gephyrin, GlyRα1 and GlyRα3 protein expressions were significantly decreased in the PGE2 group at 5 h. Confocal microscopic imaging showed expression of **e** gephyrin, **f** GlyRα1 and **g** GlyRα3 localized mainly in the neuron cells. Each group had n = 3–6 rats. Scale Bars: 20 μm; **p < 0.01, and ***p < 0.001, One-way ANOVA, followed by the LSD test

**Fig. 5:**
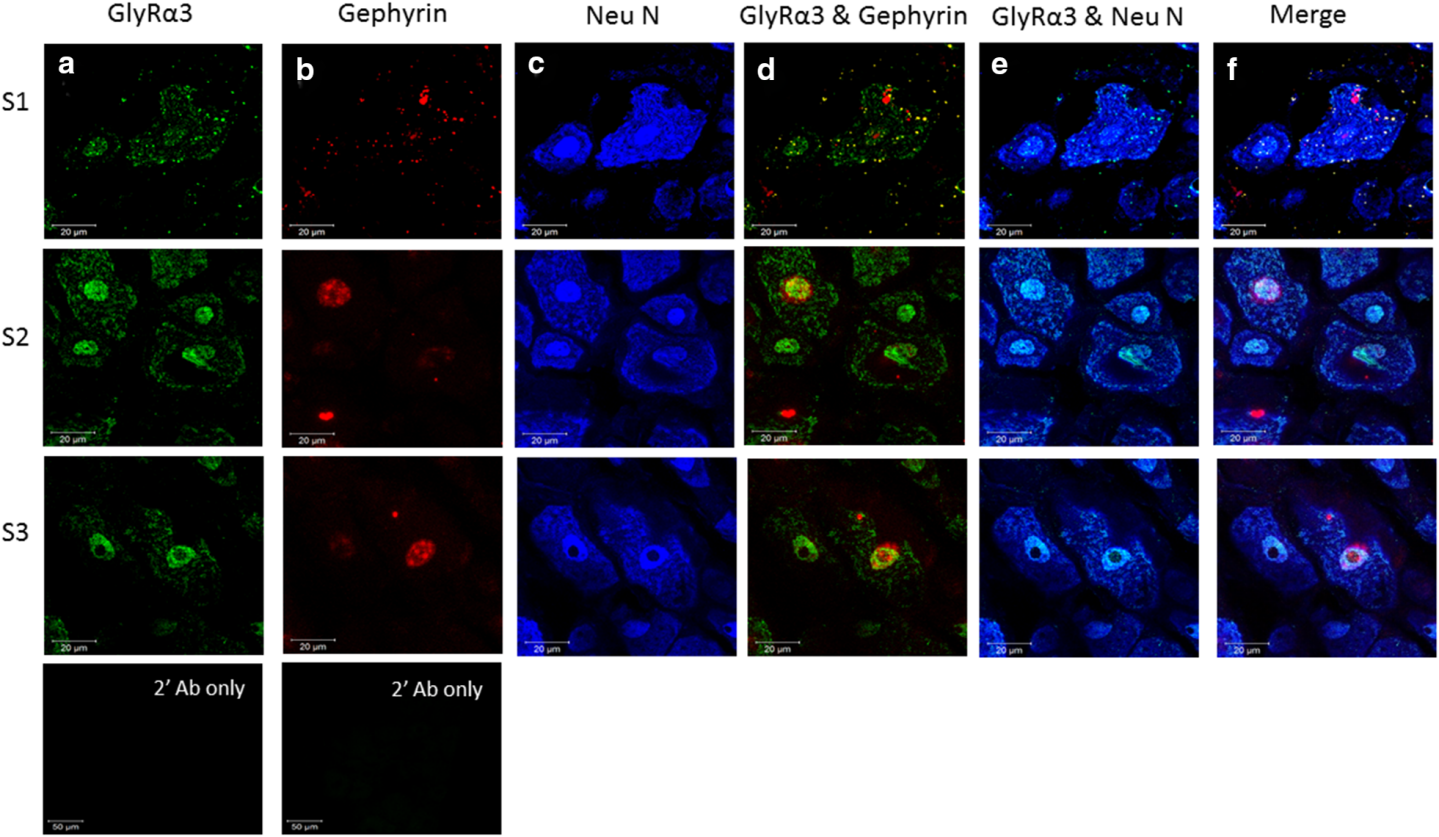
Triple immunofluorescence staining showing GlyRα3, Gephyrin and NeuN co-localization in the L5 DRG. Positive for GlyRα3 are shown in green (**a**), positive for Gephyrin are shown in red (**b**) and positive for NeuN are shown in blue (**c**). Double-labelled images of GlyRα3 and Gephyrin (**d**), GlyRα3 and NeuN (**e**) are indicated. **f** The merged image demonstrates co-localization of GlyRα3, Gephyrin in Neurons of DRG. IF experiments from three different sham controls were performed. Secondary Abs only were included as negative controls. Scale bars = 20 µm

The PGE2 intrathecal injection model significantly decreased gephyrin, GlyRα1 and GlyRα3 protein expressions in the L5 DRG at 5 h (Fig. 4; Additional file 4). These protein expressions did not significant change in the L5 DRG at 1 h. Confocal microscopic images showed the co-existence of punctate gephyrin and GlyRα3 IR in DRG neurons (Fig. 5; Additional files 5 and 6).

## Discussion

### Gephyrin, GlyRα1 and GlyRα3 expressions in the spinal cord

The location of the GlyRs subunit combinations in human adult brain and spinal cord remains largely undetermined [11, 12]. GlyRs IR was different throughout the gray matter of the spinal cord and its intensity was especially strong in the dorsal horn and ventral horn [13]. In addition, the co-expression of glycine receptors and gethyrin in human brain and spinal cord have been presented by Baer et al. [14]. Furthermore, the cell bodies and dendritic processes showed moderate to high levels of intense GlyRs puncta on their membranes of the dorsal horn superficial layer (laminia II–III) and ventral horn [13]. Harvey et al. showed that punctate GlyRα3 IR is distinctly expressed in lamina II of the spinal cord dorsal horn, and GlyRα3 subunit IR puncta were found to colocalize with gephyrin [5]. Similar with previous studies, the GlyRα3 in our study was colocalized with gephyrin throughout the gray matter of the spinal cord and was intensive in the superficial layer (laminia I–III) and ventral horn (Fig. 3). One recent study showed that temporal elevation of GlyRα1 and reduction of GlyRα2 were observed within the spinal cord following spinal cord injury [15]. In Harvey et al. study, costaining for GlyRα1 subunits and GlyRα3 subunits revealed 54 ± 3% colocalization [5]. Thus, both subunit-specific glycinergic synapses and mixed glycinergic synapses exist.

### Gephyrin, GlyRα1 and GlyRα3 expressions in dorsal root ganglion (DRG)

In our study, the western blotting of gephyrin, GlyRα1 and GlyRα3 proteins revealed that they were found in the DRG (Fig. 4). Furthermore, in the IF stain, the gephyrin and GlyRα3 receptors were localized in neuron cells (Fig. 5). In a study by Furuyama et al. expression of the β-subunit mRNA of the glycine receptor in the rat DRG was examined. About 44% of all DRG neurons were labeled by the probes for glycine receptor β-subunit mRNAs and the labeled neurons were mostly large cells [16]. However, in another study by Harvey et al. no specific punctate GlyRα3 or GlyRα1 IR was detectable in the DRG in fluorescence micrographs taken from a single section through the thoracic spinal cord [5]. In our experiments, we used the same antibody to stain the gephyrin, GlyRα1 and GlyRα3 receptors in the spinal cord and DRG, and both western blots and IF showed the same results. The different results in terms of the DRG between our study and the Harvey et al. study may be due to different DRG samples or antibody specificity. The fluorescence micrographs were taken from L5 DRG in our experiment, but they were taken from thoracic DRG in the Harvey et al. study.

### PGE2 intrathecal injection induced significant hyperalgesic responses and the time course was consistent with gephyrin, GlyRα1 and GlyRα3 expression in DH and DRG

In our experiments, the pain behavior of the rat was significantly different at 3 h and continued to decrease to 5 h post PGE2 injection compared with vehicle injection. The GlyRα1 and GlyRα3 in DH were significantly decreased from 1 to 5 h post PGE2 injection. The GlyRα1 and GlyRα3 in DRG did not have a significant change at 1 h, but was significantly decreased at 5 h post PGE2 injection. Previous studies showed the PGE2 intrathecal injection induced hyperalgesic responses were through GlyRα3 inhibition in the spinal cord dorsal horn [5, 17–19]. However, comparing those behavior changes with GlyRs expression changes in DH and in DRG in our study showed GlyRs in DRG should play a role in PGE2 induced pain behavior. Thus, the authors suggest the possibility that glycine presynaptically regulates the activity of neurons involved in low-threshold mechanoreception at axoaxonic synapses in the spinal cord [16].

In Harvey et al.’s study, the behavior was significant changed at 10 min after PGE2 intrathecal injection. This difference between our study and the Harvey et al. study may be due to different experimental animals (rat vs. mouse) and different PGE2 dose injection (625 ng and 70 ng, respectively). Furthermore, mice are more sensitive to pain stimulation [20].

This study showed PGE2 intrathecal injection significantly decreased gephyrin, GlyRα1 and GlyRα3 expression in DRG. IF revealed the gephyrin and GlyRα3 were localized on neuron cells. However, the interaction of these receptors with neuron cells needs further evaluation. In our experiments, we were not able to specifically suppress the function of these glycine receptors in the spinal cord or in the DRG, so we were not able to determine how much of a role these glycine receptors play in PGE2-induced pain. This question needs to be further explored in future experiments.

## Conclusions

This study showed PGE2 intrathecal injection significantly decreased gephyrin, GlyRα1 and GlyRα3 protein expressions in spinal cord DH and DRG. Through IF, it indicated that the gephyrin and GlyRα3 were localized on neuron cells. However, the exact nature of the interaction of these receptors with neuron cells needs further evaluation.

## Additional files

**Additional file 1.** Behavior; Intrathecal injection with PGE2 induced hyperalgesic responses in rats.

**Additional file 2: Fig. S1.** **a** Western blot and quantitative analysis of (**b**) GlyRα1 protein expressions in L5 spinal cord dorsal horn at 1 h and 5 h after PGE2 intrathecal injection. The GlyRα1 protein expressions were significantly decreased in the PGE2 group at 1 h and this lasted up to 5 h. Triple immunofluorescence staining showing GlyRα1, Gephyrin and NeuN co-localization in the L5 spinal cord dorsal horn. Positive for GlyRα1 are shown in green (**c**), positive for Gephyrin are shown in red (**d**) and positive for NeuN are shown in blue (**e**). Double-labelled images of GlyRα1 and Gephyrin (**f**), GlyRα1 and NeuN (**g**) are indicated. **h** The merged image demonstrates co-localization of GlyRα1, Gephyrin in Neurons. Scale bars = 200 µm. Each group had n = 3–6 rats, **p < 0.01, One-way ANOVA, followed by the LSD test.

**Additional file 3.** Western blot in L5 spinal cord dorsal horn; Western blot and quantitative analysis of gephyrin, GlyRα1 and GlyRα3 expressions in L5 spinal cord dorsal horn at 1 h and 5 h after PGE2 intrathecal injection.

**Additional file 4.** Western blot in L5 DRG; Western blot and quantitative analysis of gephyrin, GlyRα1 and GlyRα3 expressions in L5 DRG at 1 h and 5 h after PGE2 intrathecal injection.

**Additional file 5.** Triple immunofluorescence staining in the L5 DRG (Control group); Triple immunofluorescence staining showing GlyRα3, Gephyrin and NeuN co-localization in the L5 DRG.

**Additional file 6.** Triple immunofluorescence staining in the L5 DRG; Triple immunofluorescence staining showing GlyRα3, Gephyrin and NeuN co-localization in the L5 DRG.
