# Supplementary figures and images for: Glycine receptors expression in rat spinal cord and dorsal root ganglion in prostaglandin E2 intrathecal injection models
Source: BMC Neurosci. 2018 Nov 9;19:72. doi: 10.1186/s12868-018-0470-8 (PMC6230273; doi:10.1186/s12868-018-0470-8)

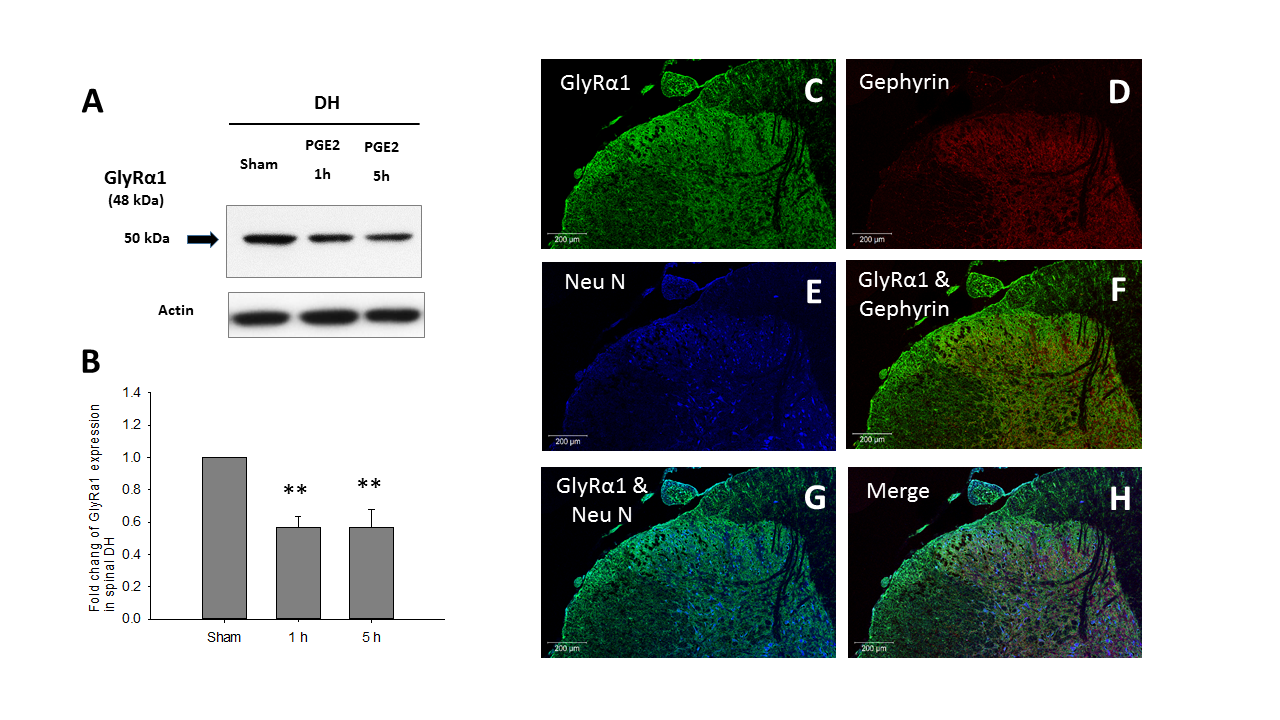

Supplement: Supplementary file 2 — Additional file 2: Fig. S1. a Western blot and quantitative analysis of (b) GlyRα1 protein expressions in L5 spinal cord dorsal horn at 1 h and 5 h after PGE2 intrathecal injection. The GlyRα1 protein expressions were significantly decreased in the PGE2 group at 1 h and this lasted up to 5 h. Triple immunofluorescence staining showing GlyRα1, Gephyrin and NeuN co-localization in the L5 spinal cord dorsal horn. Positive for GlyRα1 are shown in green (c), positive for Gephyrin are shown in red (d) and positive for NeuN are shown in blue (e). Double-labelled images of GlyRα1 and Gephyrin (f), GlyRα1 and NeuN (g) are indicated. h The merged image demonstrates co-localization of GlyRα1, Gephyrin in Neurons. Scale bars = 200 µm. Each group had n = 3–6 rats, **p < 0.01, One-way ANOVA, followed by the LSD test. [file 12868_2018_470_MOESM2_ESM.tif]
