# Supplementary material for: Glycine receptors expression in rat spinal cord and dorsal root ganglion in prostaglandin E2 intrathecal injection models
Source: BMC Neurosci. 2018 Nov 9;19:72. doi: 10.1186/s12868-018-0470-8 (PMC6230273; doi:10.1186/s12868-018-0470-8)

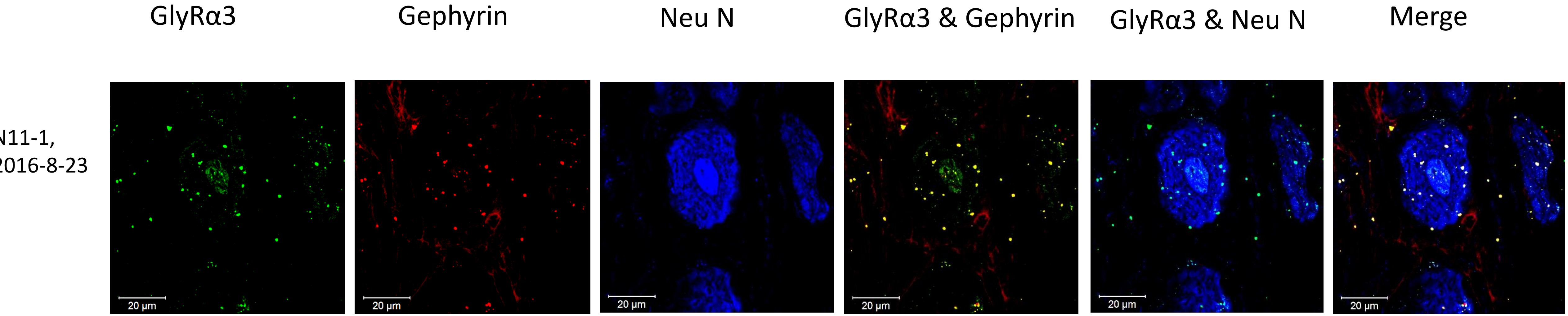
GlyRα3
Gephyrin
Neu N
GlyRα3 & Gephyrin
GlyRα3 & Neu N
Merge
N11-1,
2016-8-23
20 µm

N11-1, Gephyrin
2016-8-23

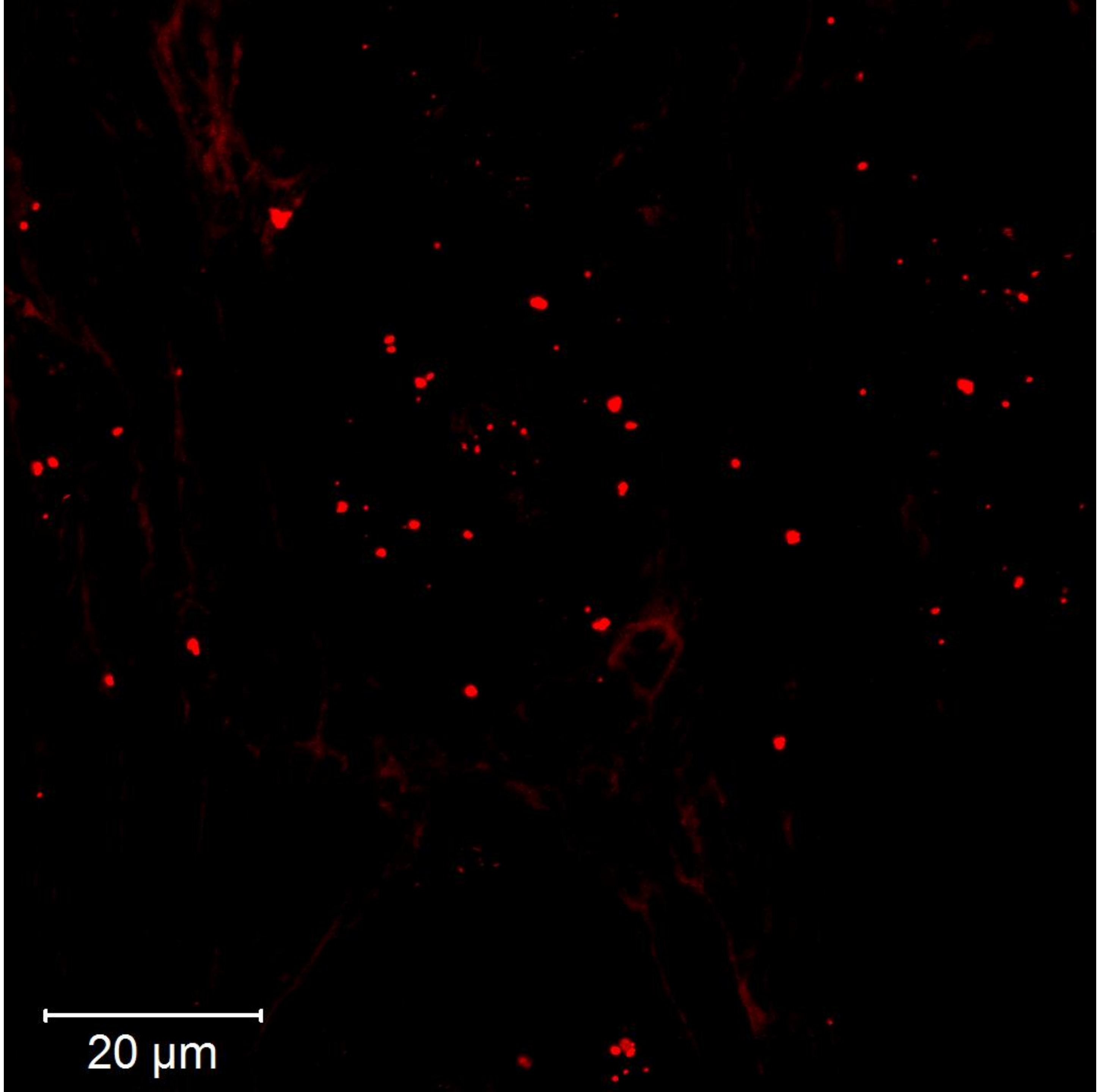

N11-1, Gephyrin & NeuN
2016-8-23

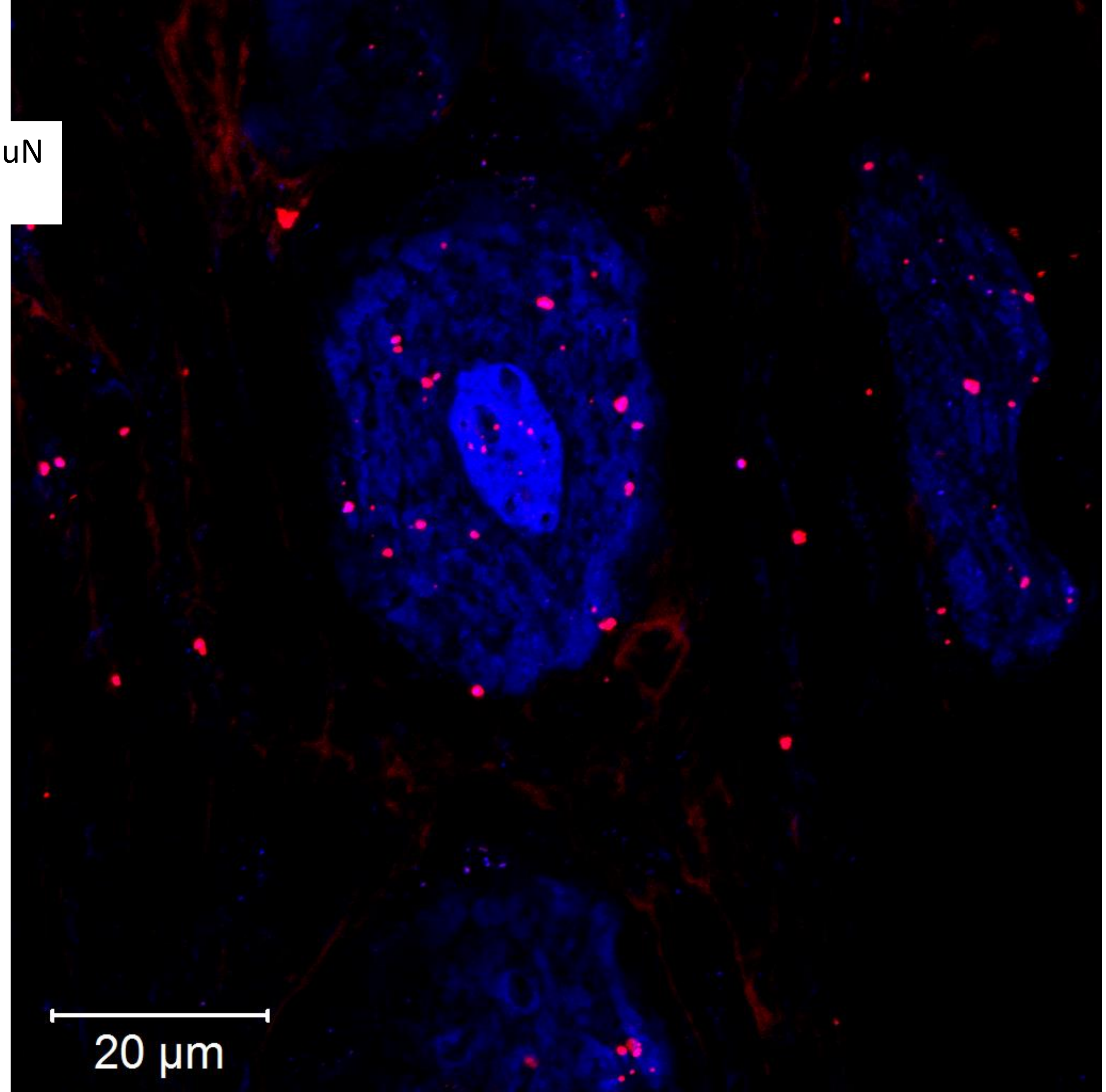

N11-1, GlyRa3
2016-8-23

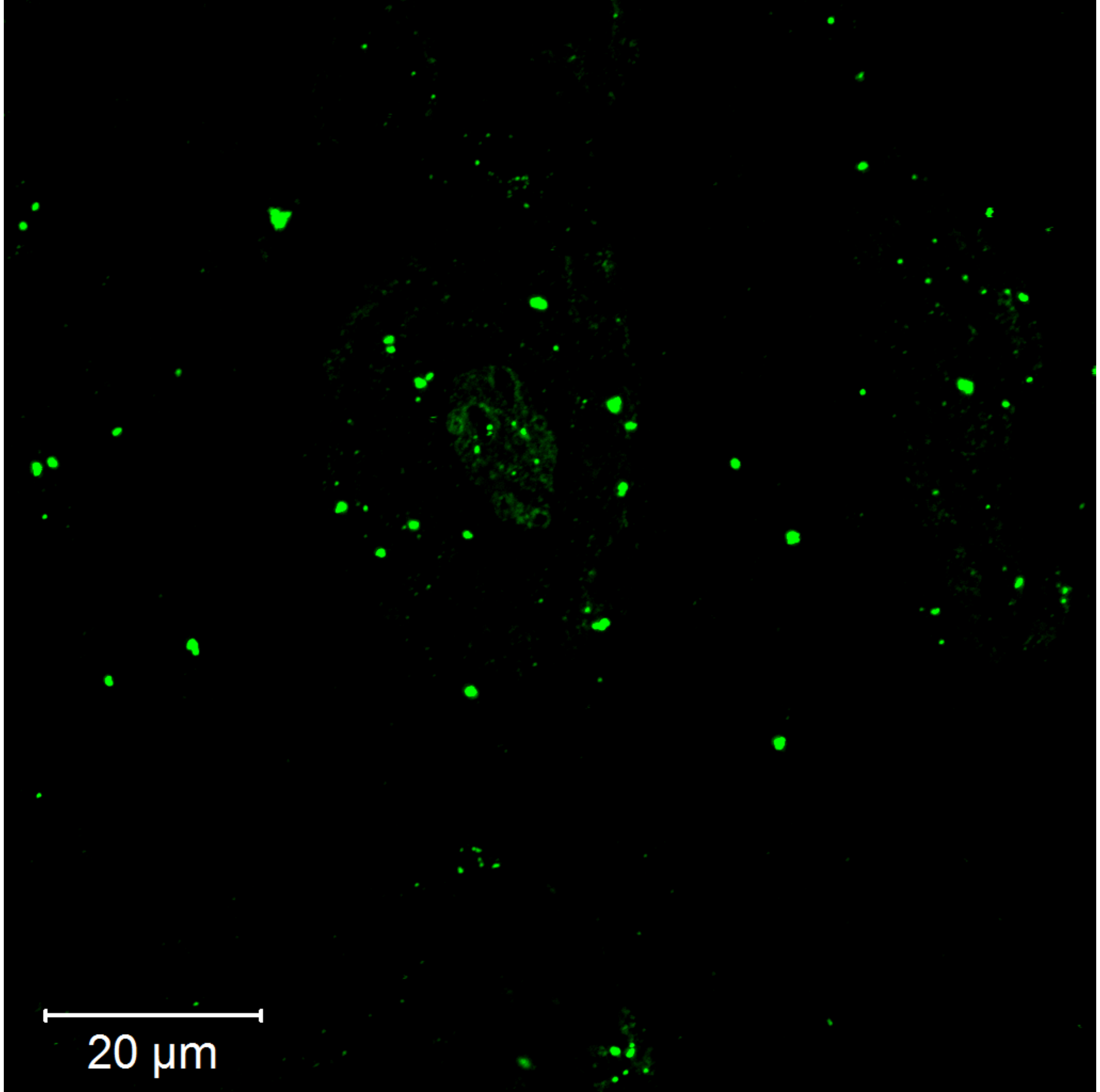

N11-1, GlyRa3 & Gephyrin
2016-8-23

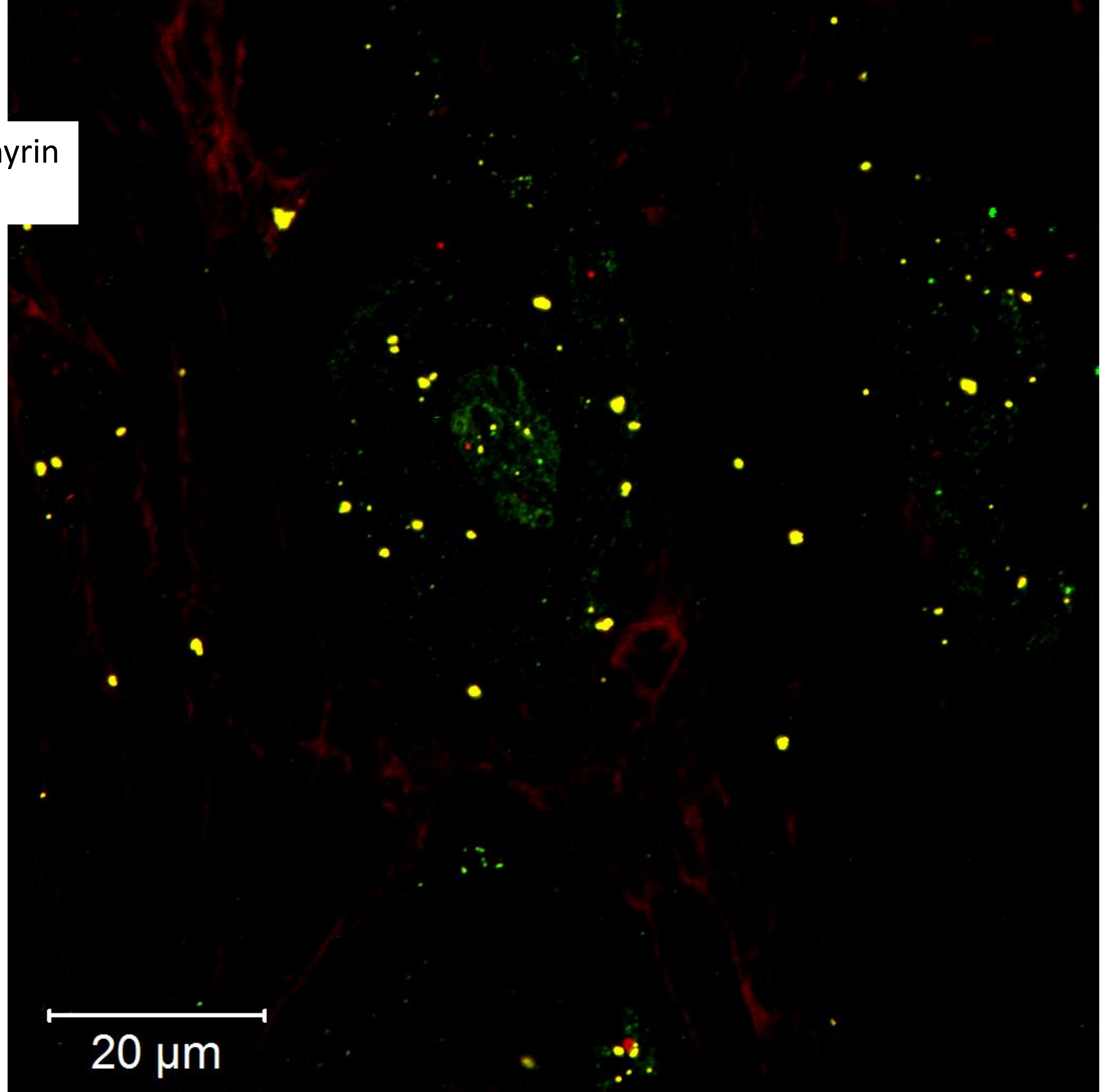

N11-1, GlyRa3 & Gephyrin & NeuN
2016-8-23

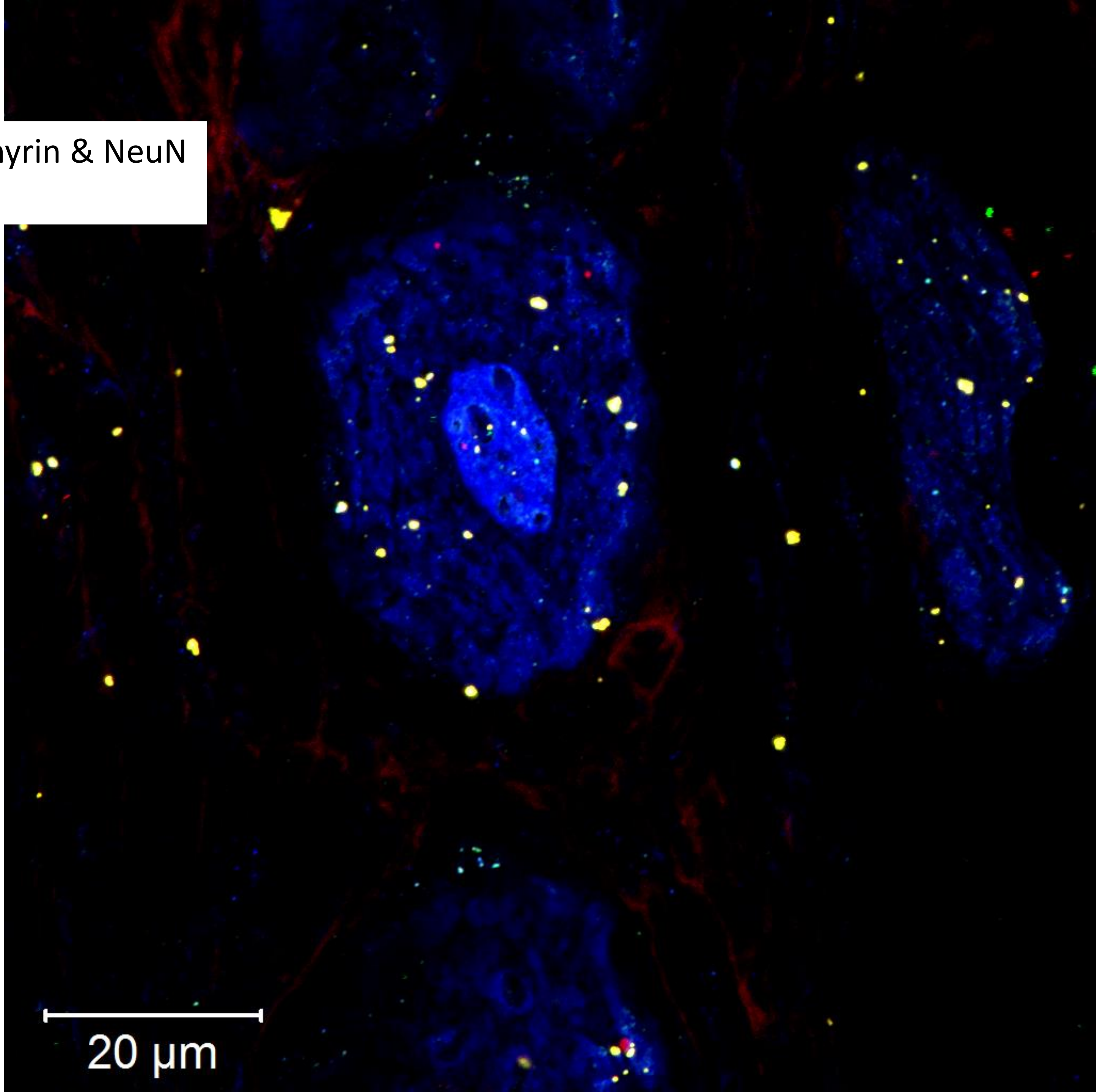

N11-1, GlyRa3 & NeuN
2016-8-23

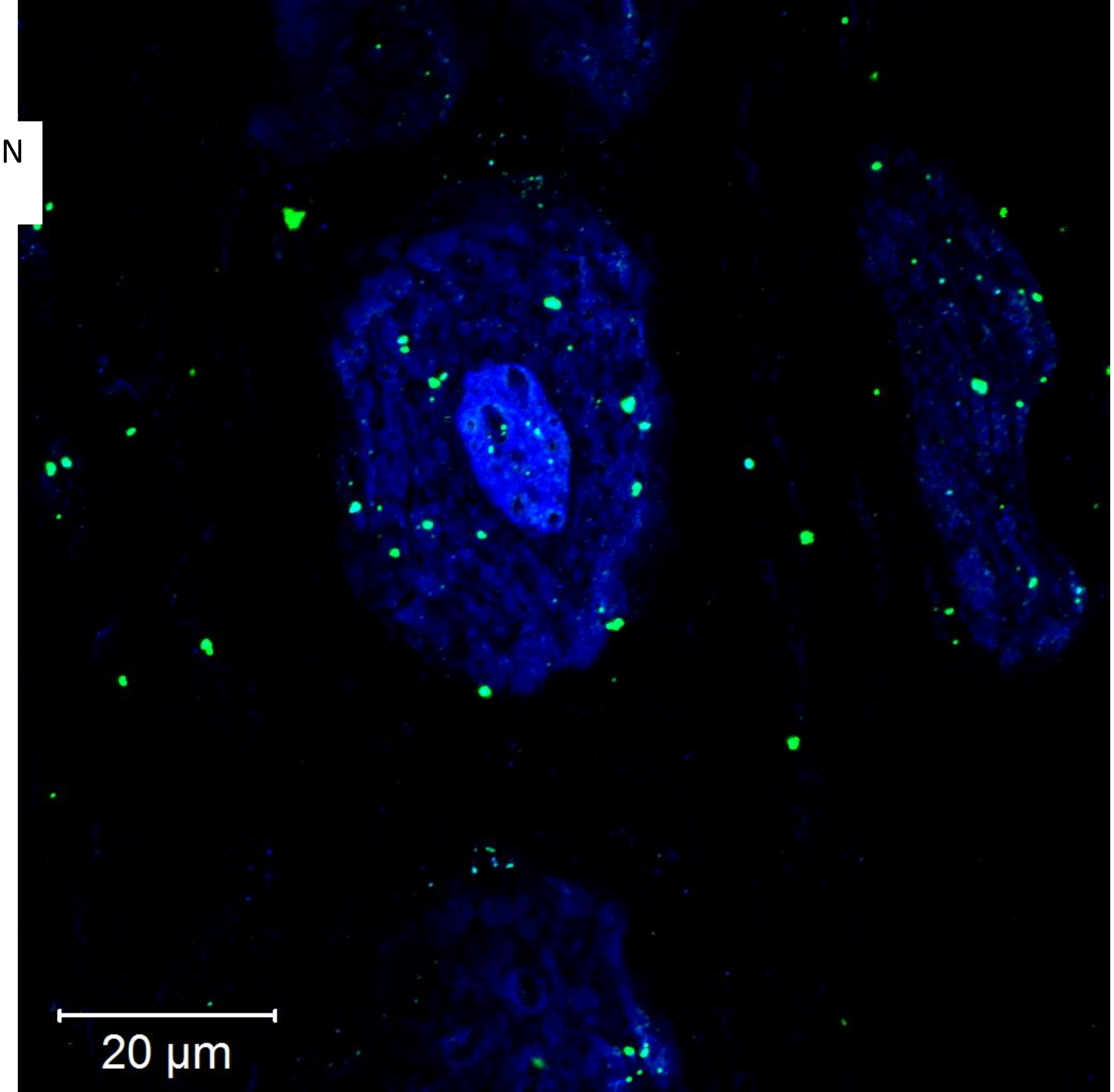

N11-1, NeuN
2016-8-23

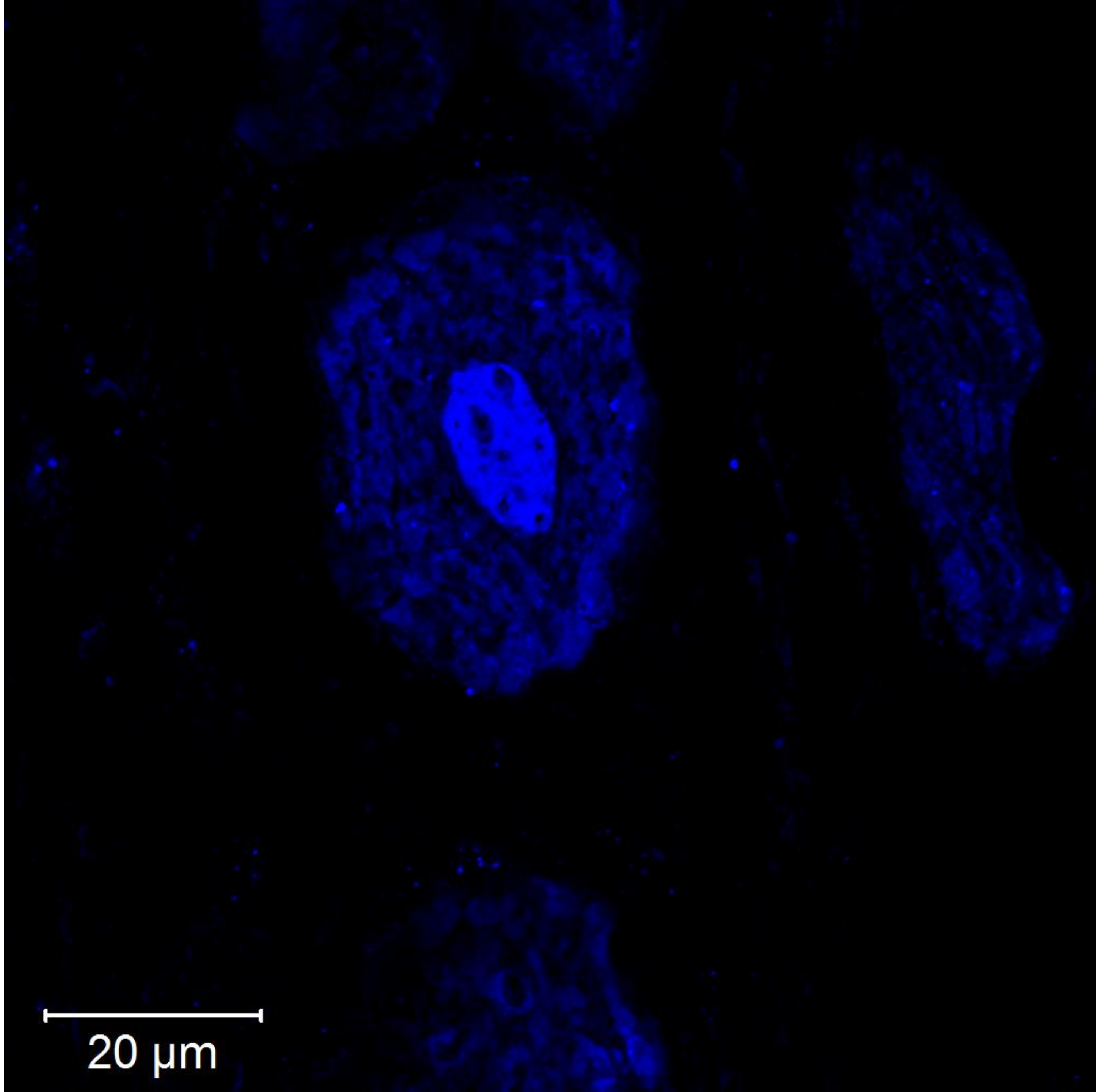

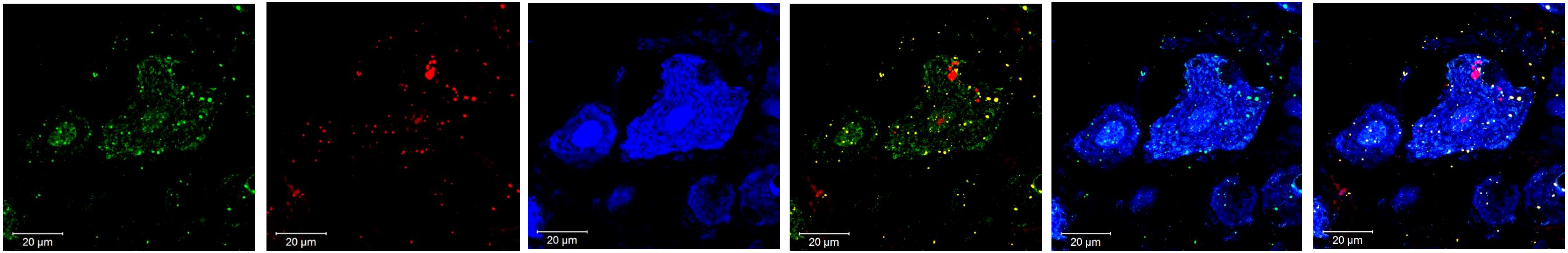
GlyRα3
Gephyrin
Neu N
GlyRα3 & Gephyrin
GlyRα3 & Neu N
Merge
1-2,
16-8-23
20 μm
20 μm
20 μm
20 μm
20 μm
20 μm

N11-2, Gephyrin
2016-8-23

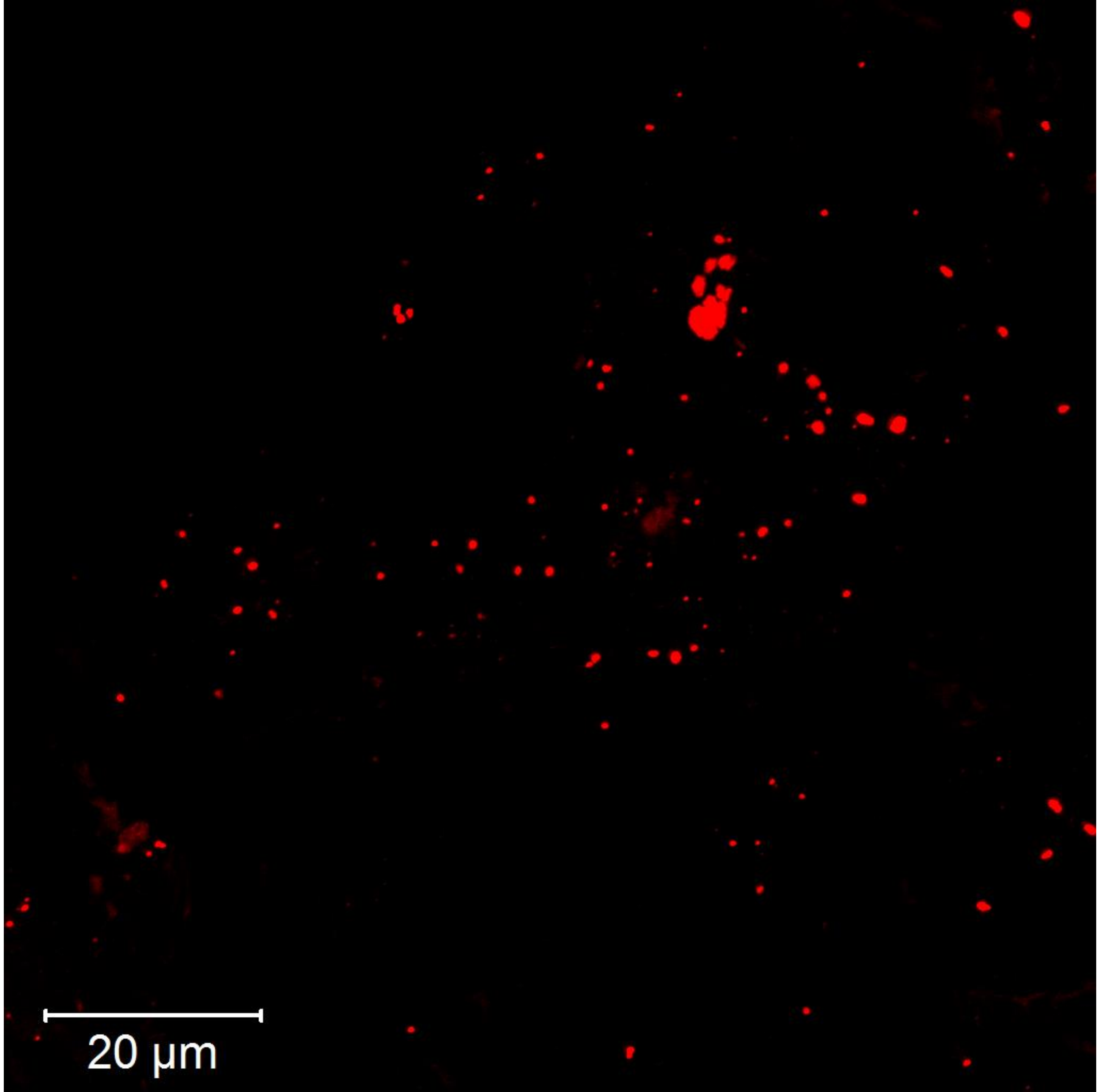

N11-2, Gephyrin & NeuN
2016-8-23

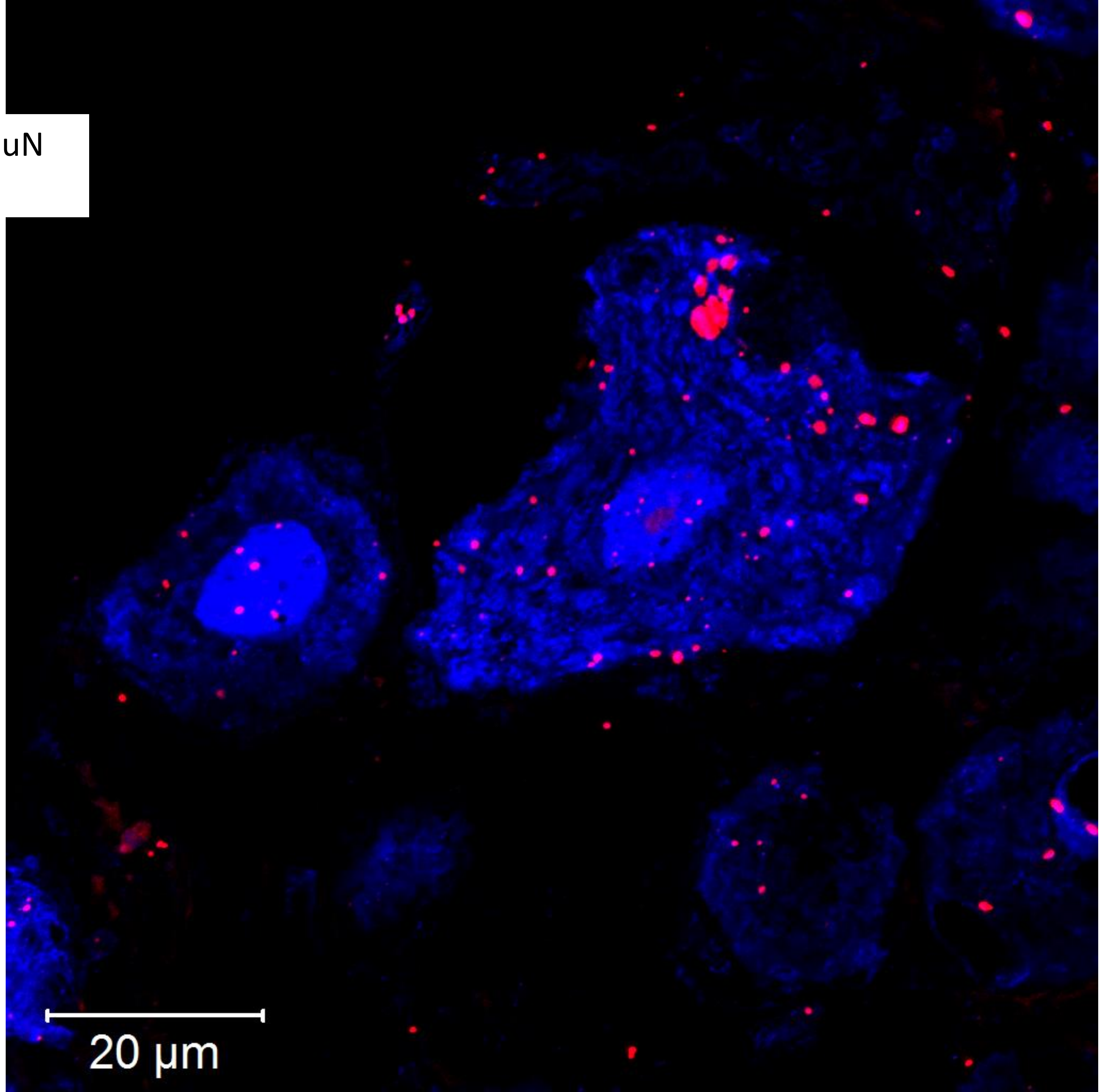

N11-2, GlyRa3
2016-8-23

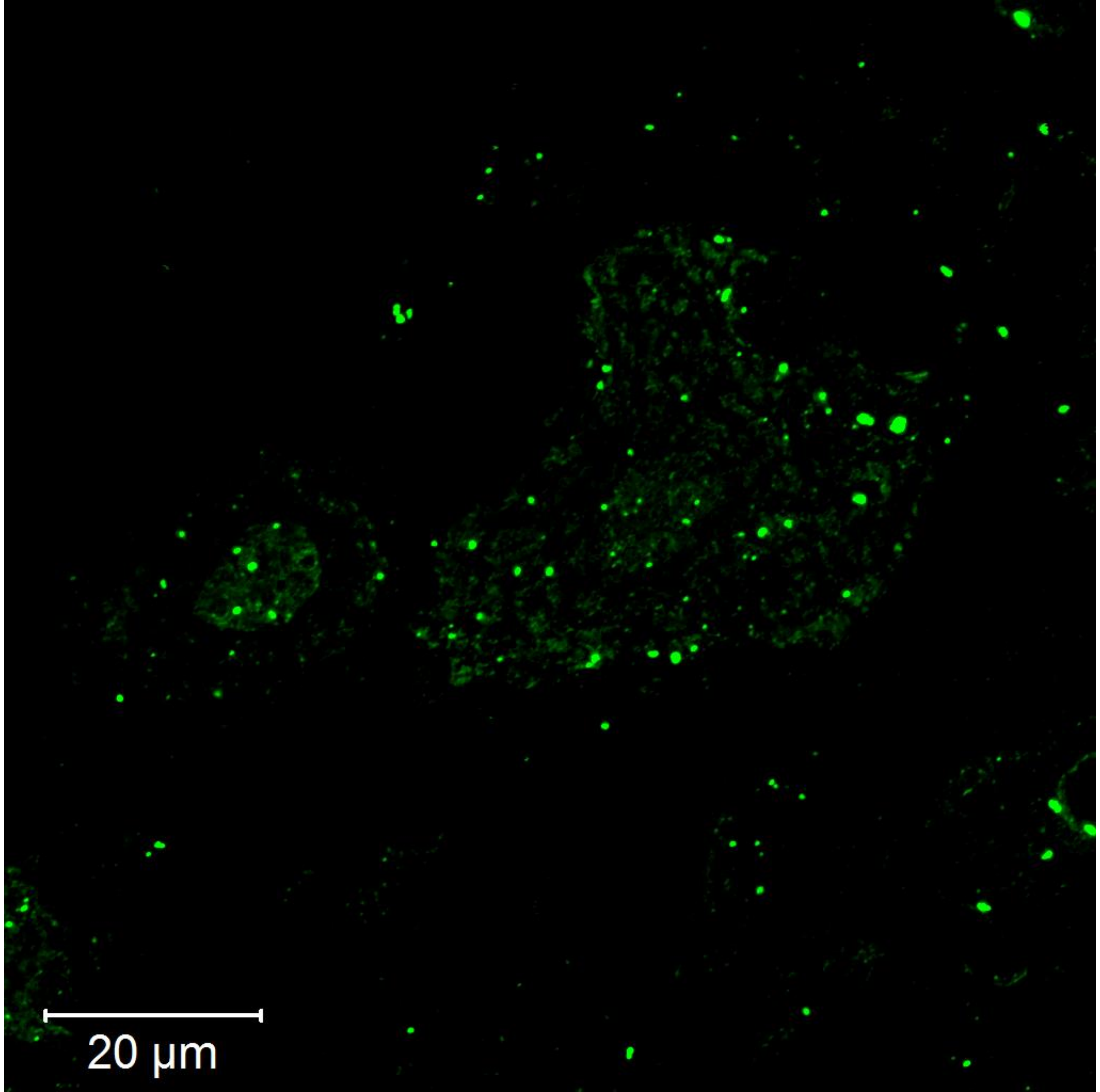

N11-2, GlyRa3 & Gephyrin
2016-8-23

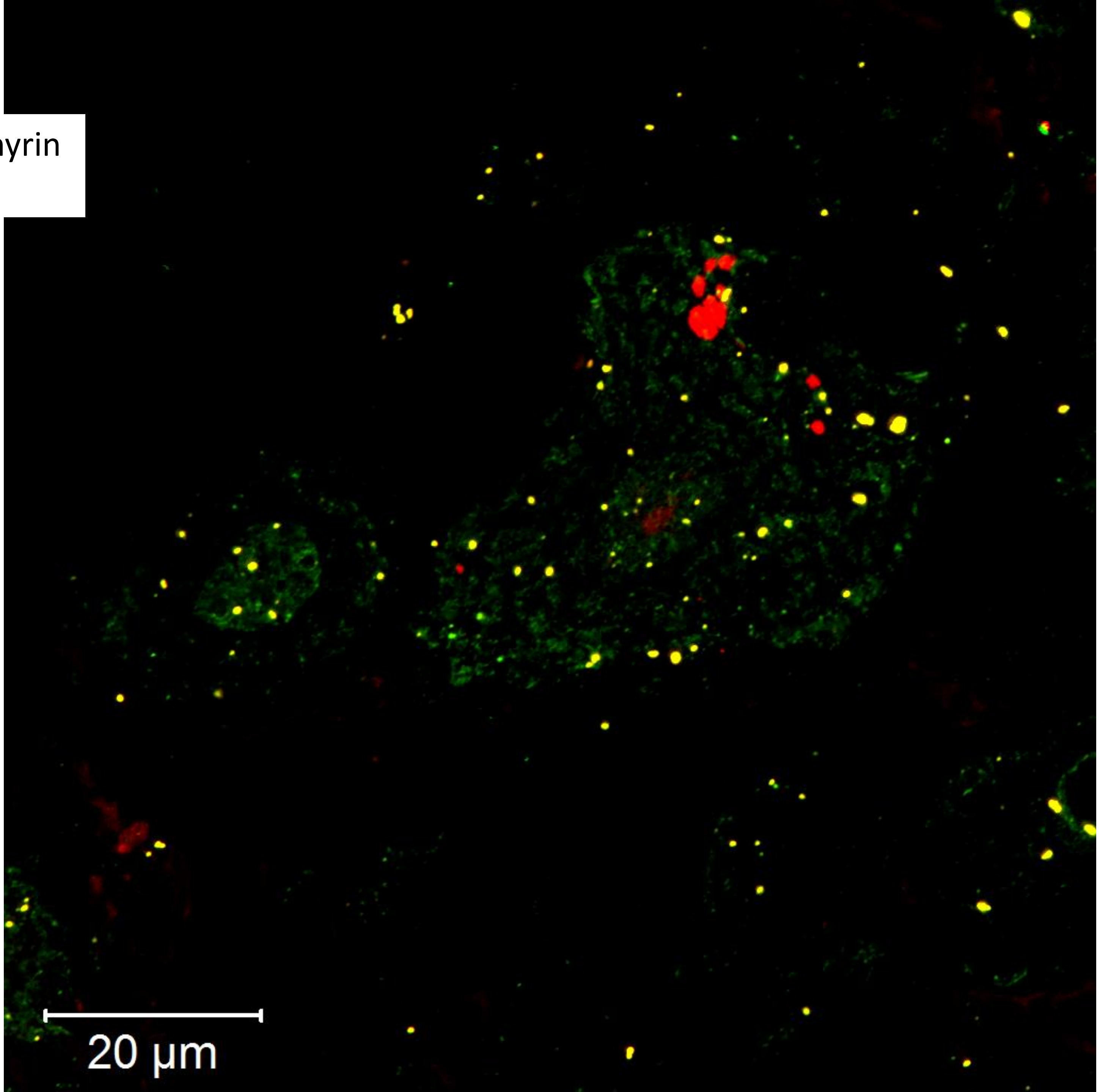

N11-2, GlyRa3 & Gephyrin & NeuN
2016-8-23

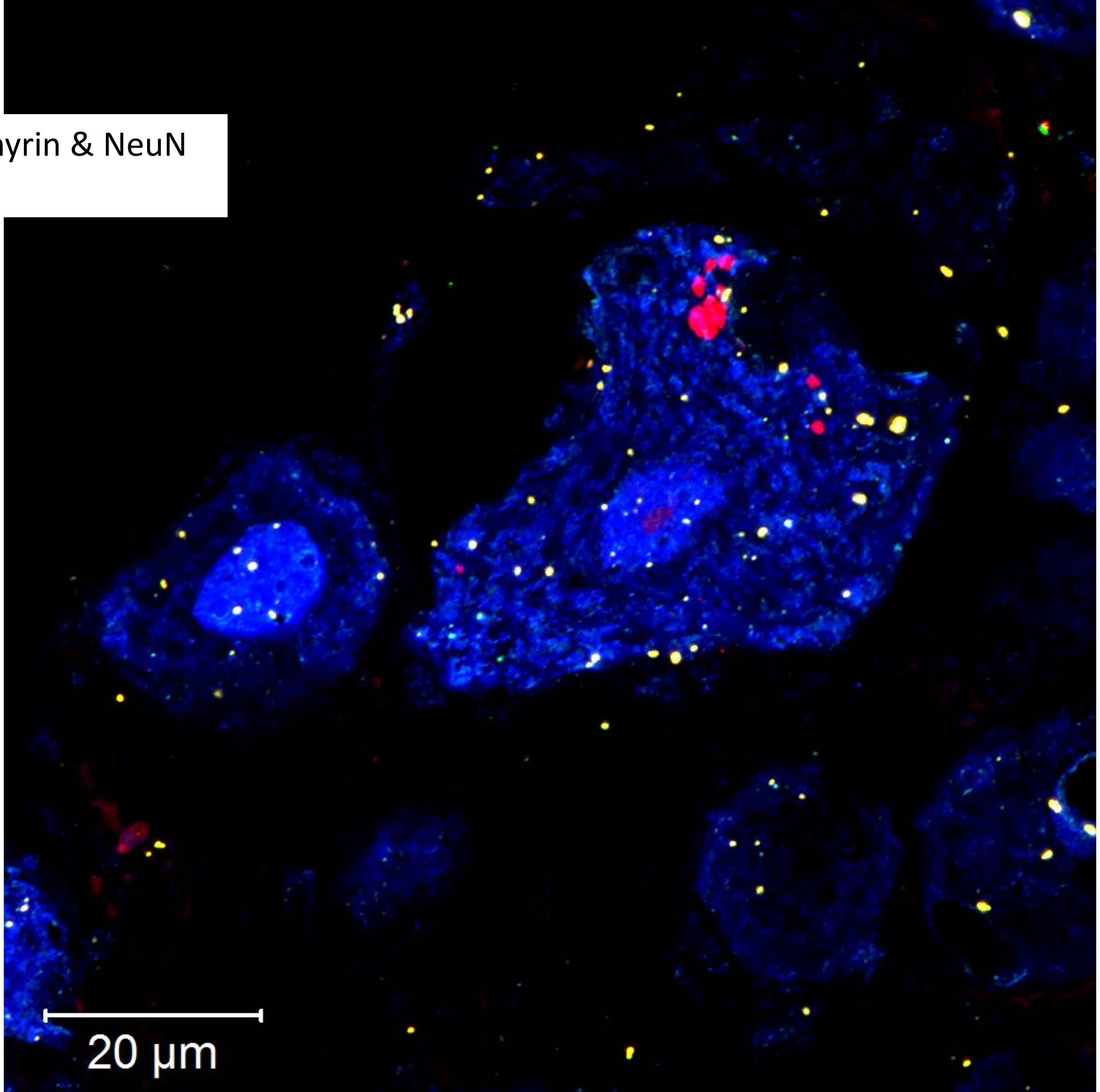

N11-2, GlyRa3 & NeuN
2016-8-23

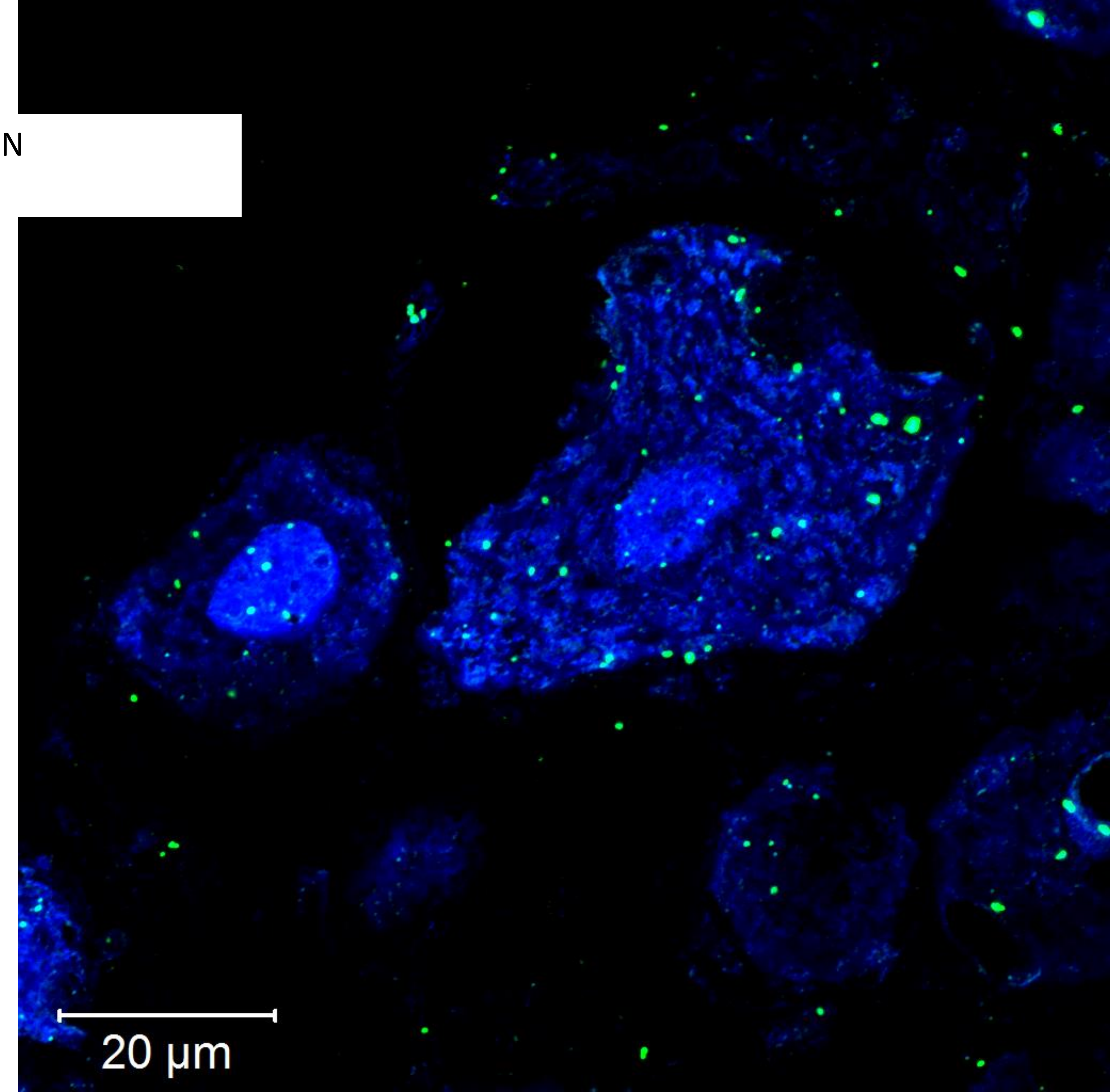

N11-2, NeuN
2016-8-23

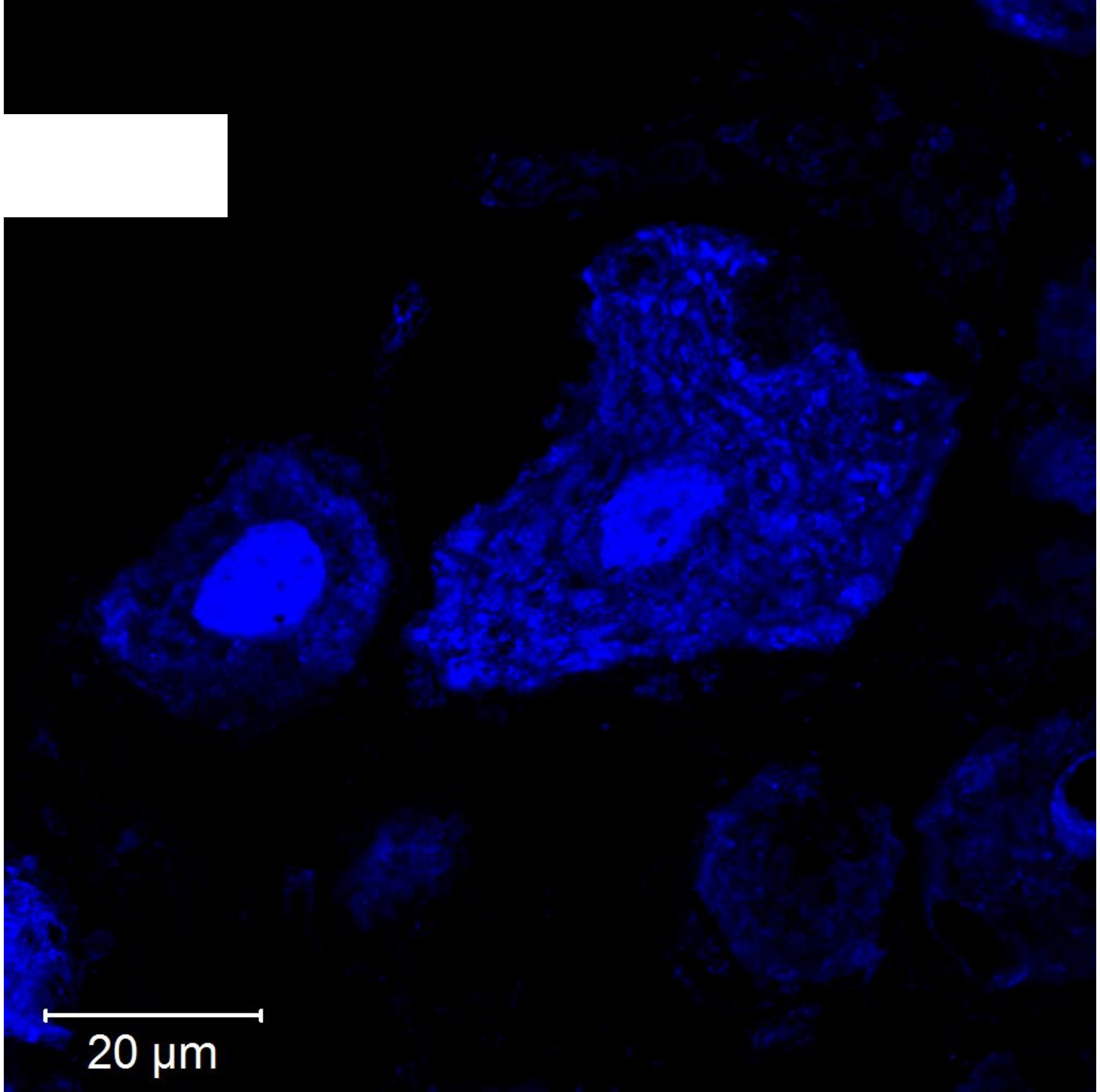

N11-3, Gephyrin
2016-8-23

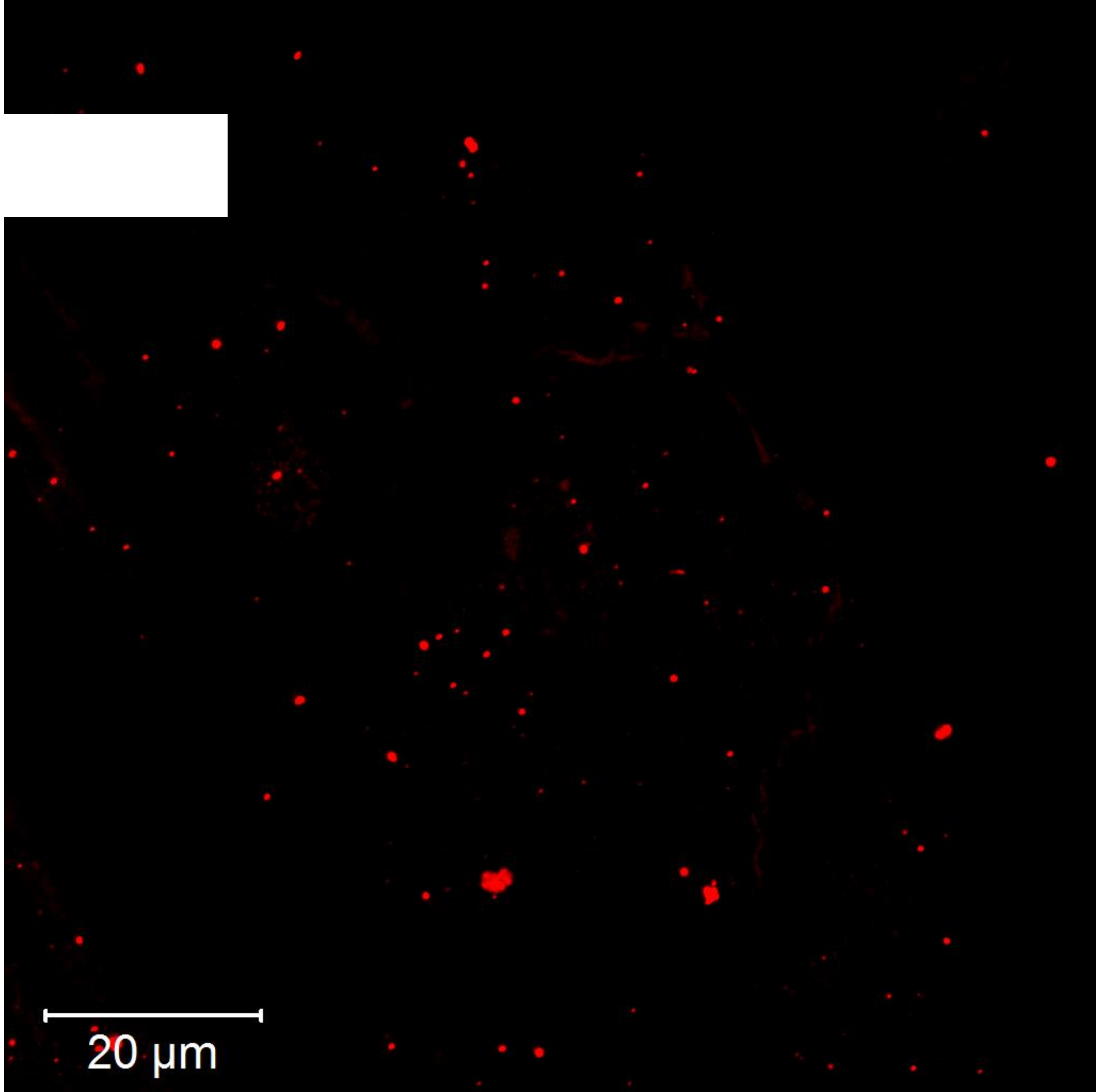

N11-3, Gephyrin & NeuN
2016-8-23

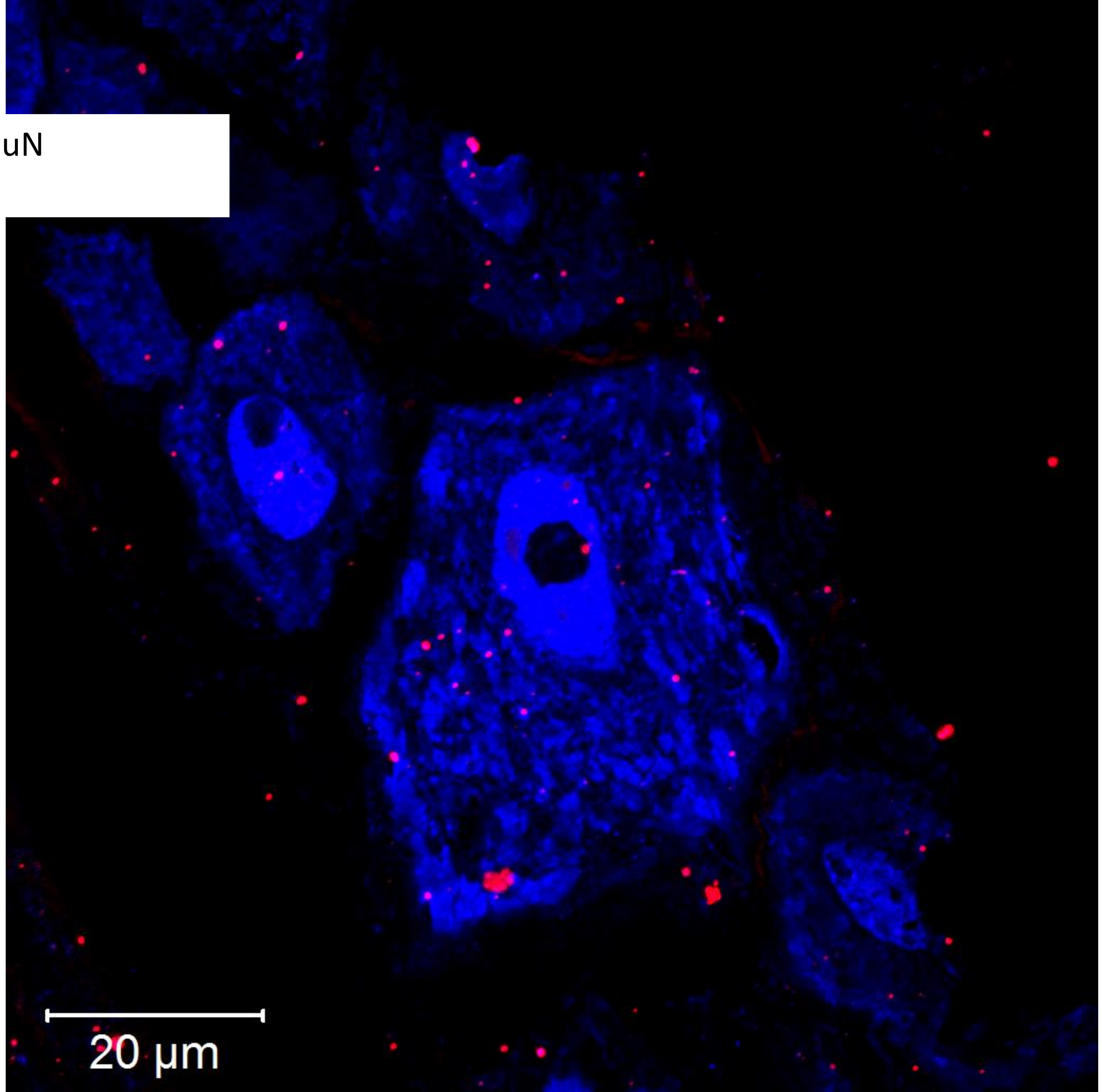

N11-3, GlyRa3
2016-8-23

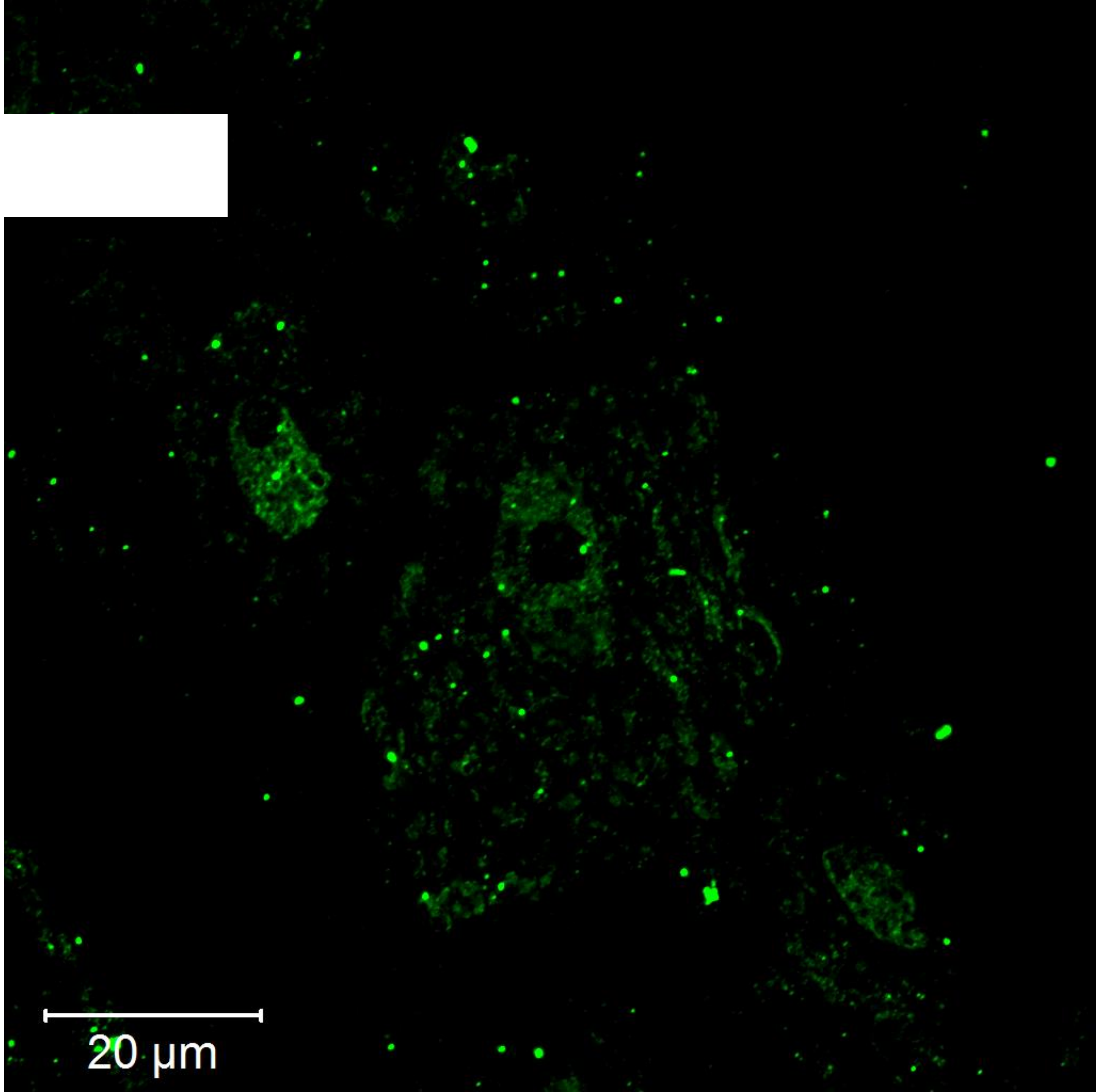

N11-3, GlyRa3 & Gephyrin
2016-8-23

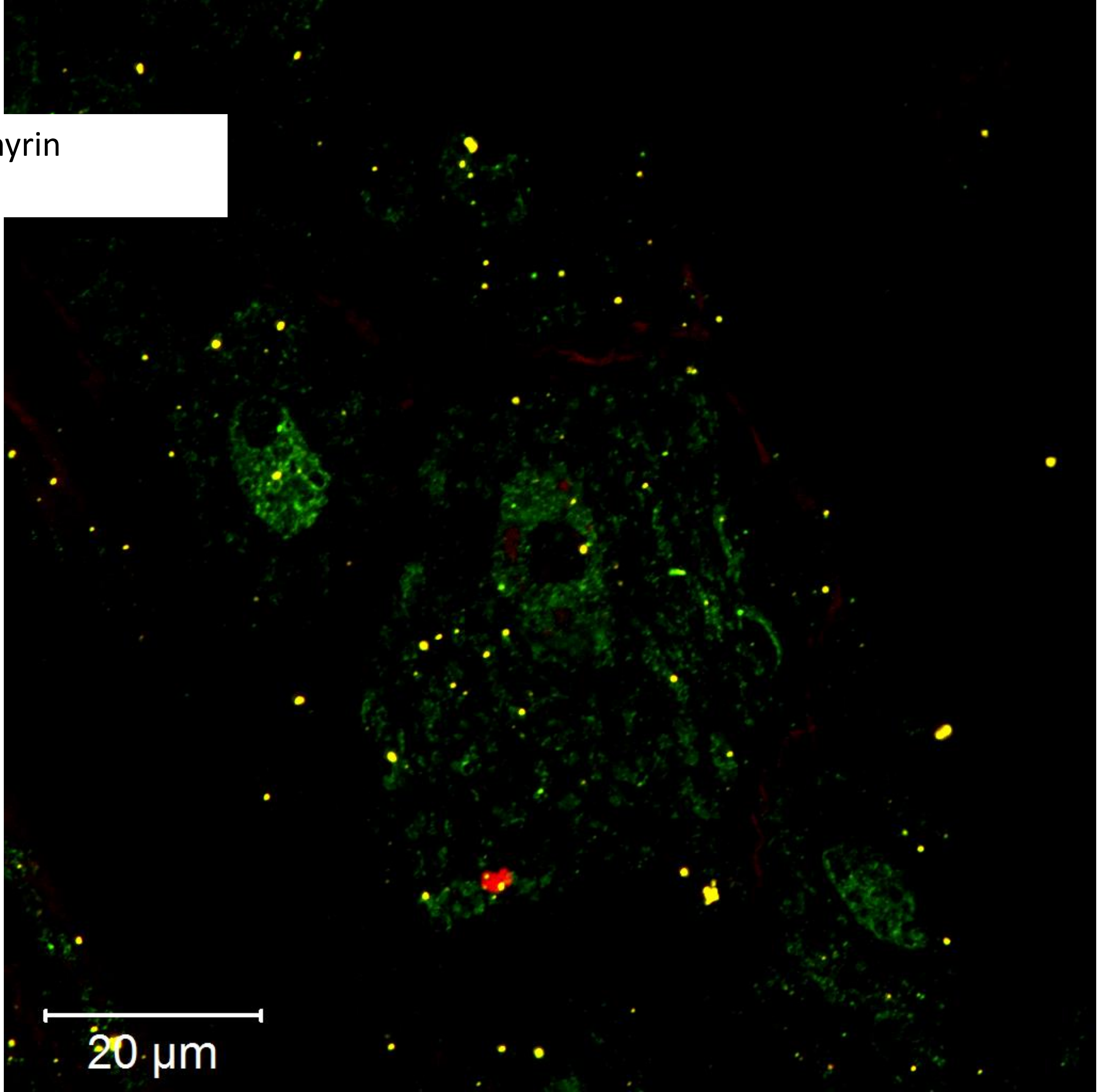

N11-3, GlyRa3 & Gephyrin & NeuN
2016-8-23

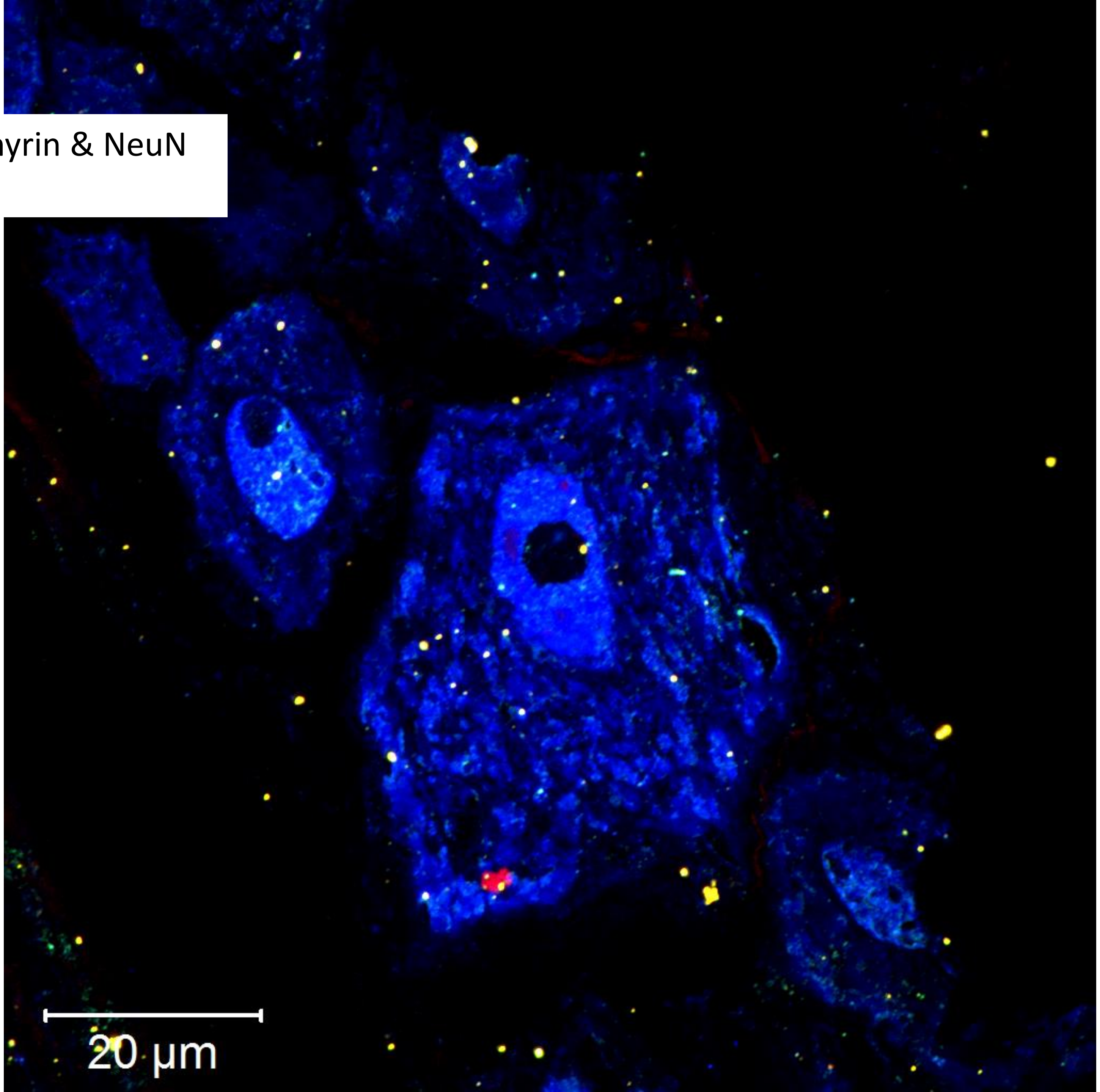

N11-3, GlyRa3 & NeuN
2016-8-23

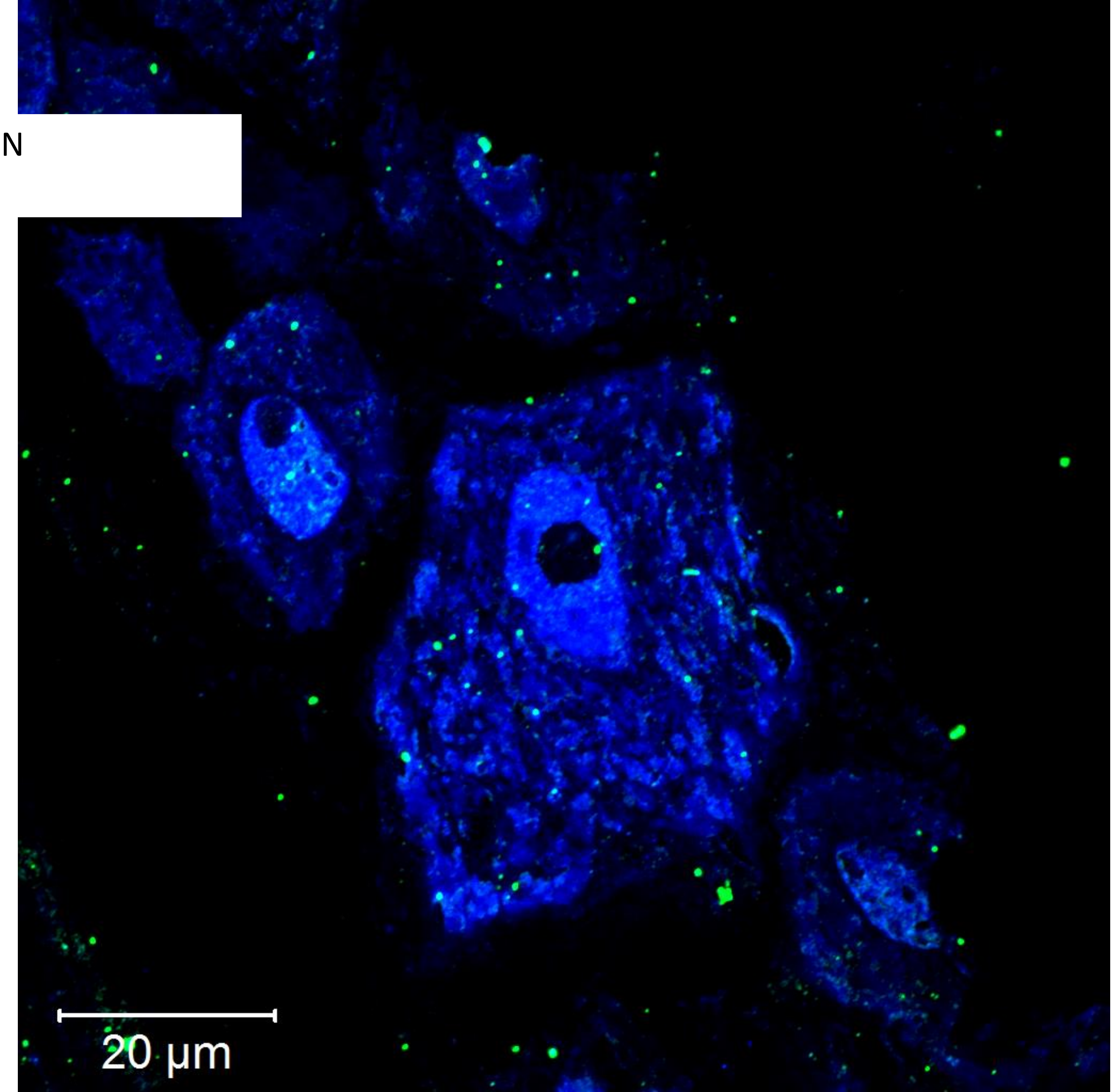

N11-3, NeuN
2016-8-23

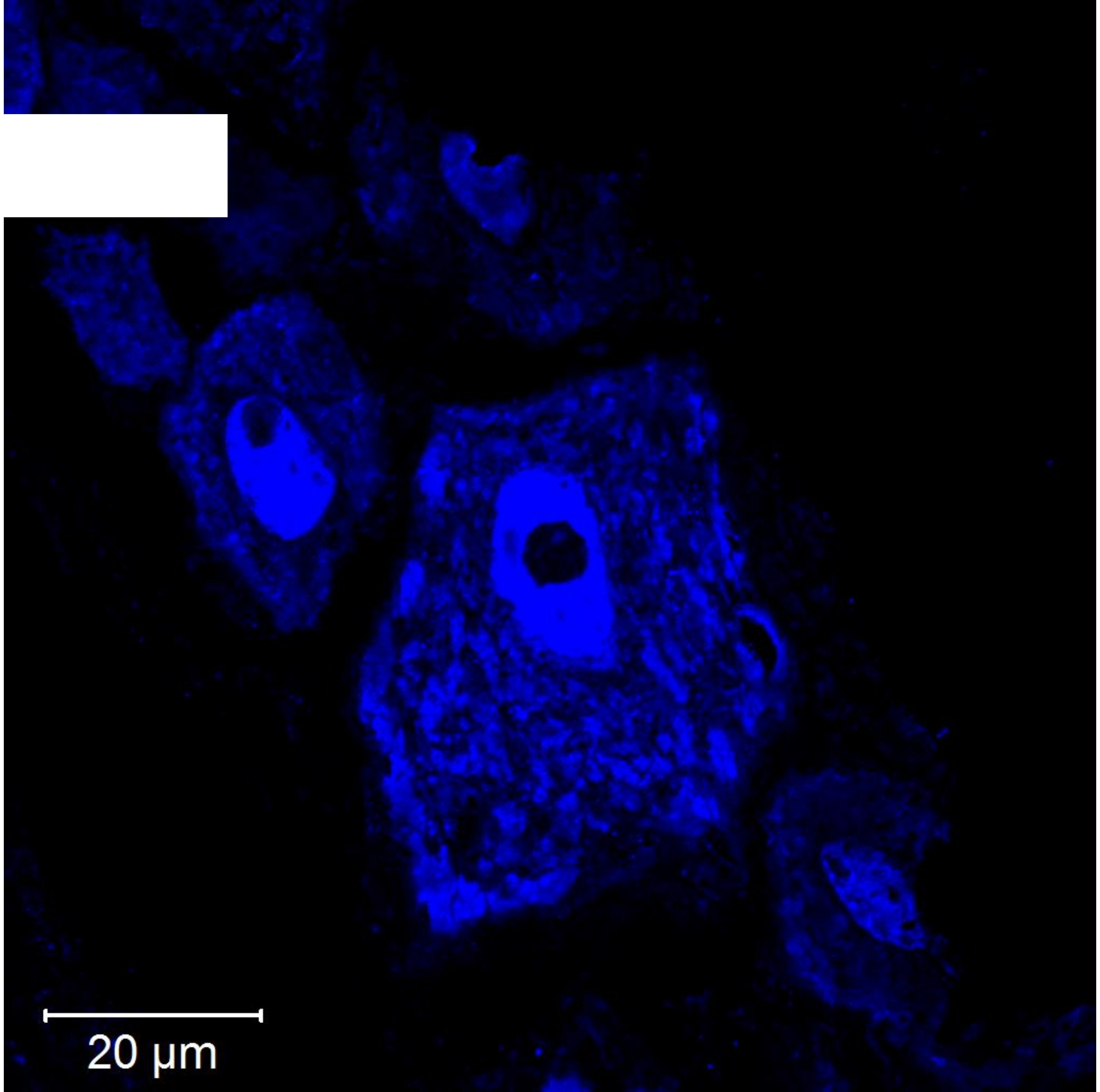

N11-4, Gephyrin
2016-8-23

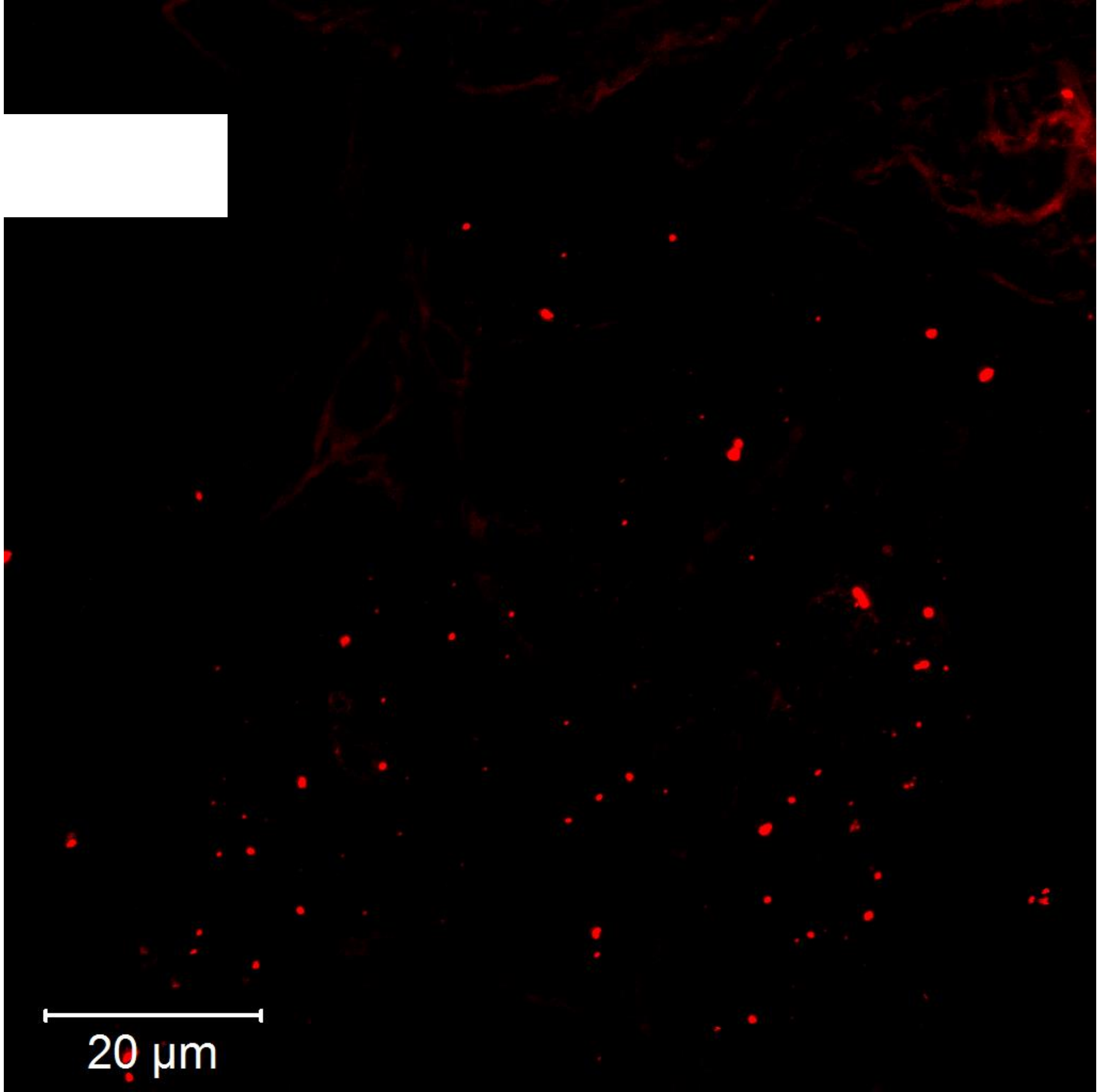

N11-4, Gephyrin & NeuN
2016-8-23

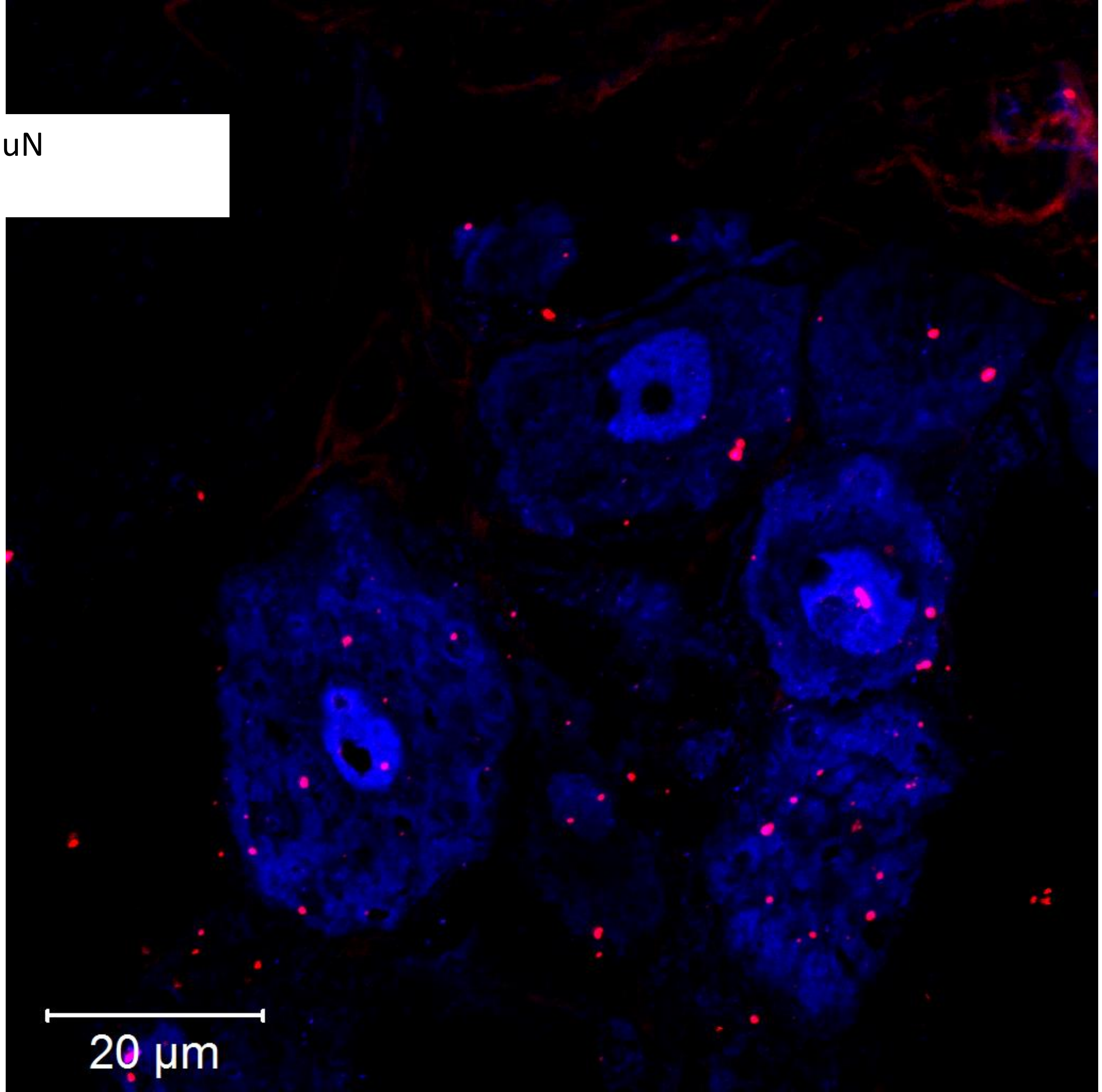

N11-4, GlyRa3
2016-8-23

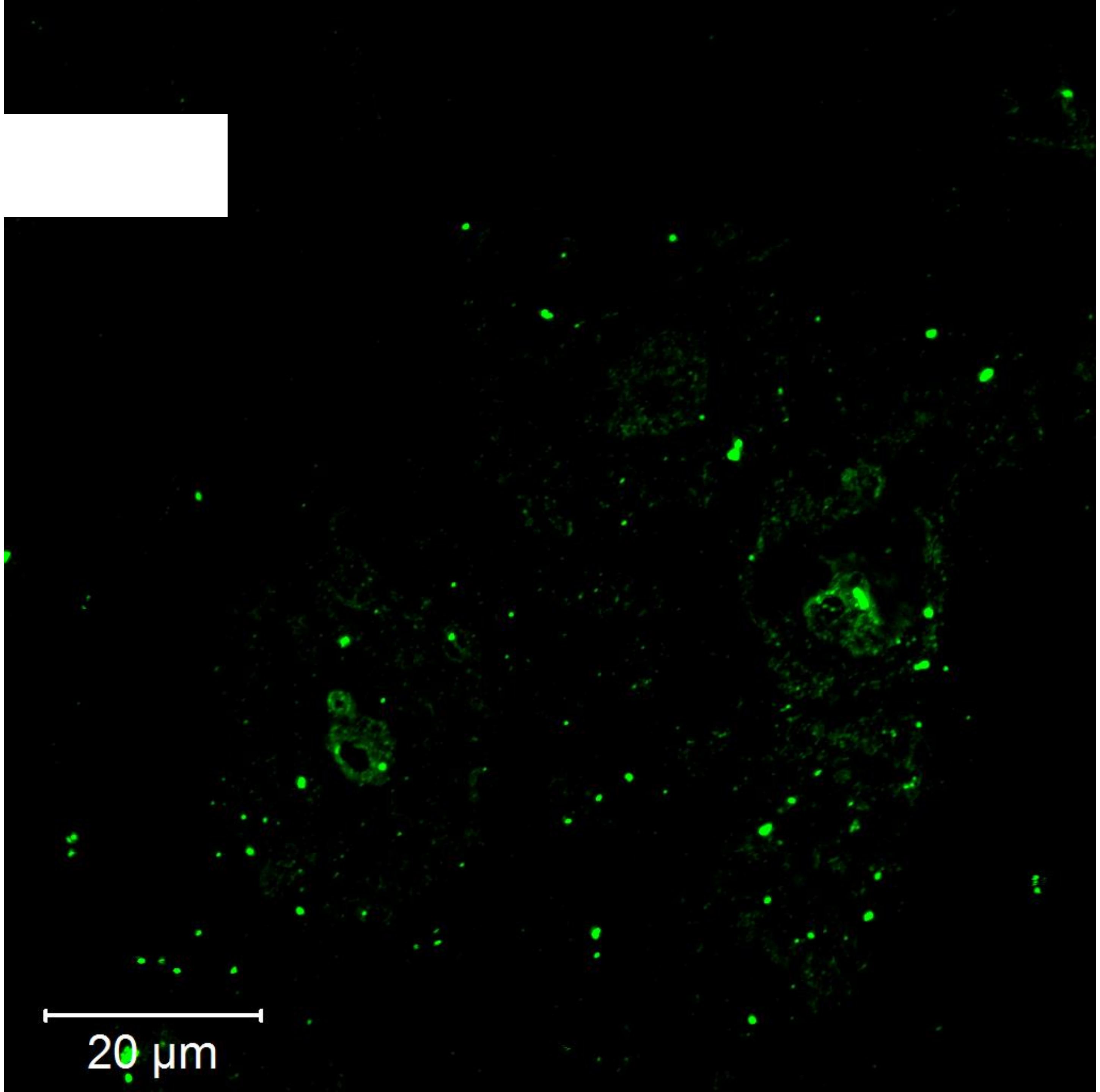

N11-4, GlyRa3 & Gephyrin
2016-8-23

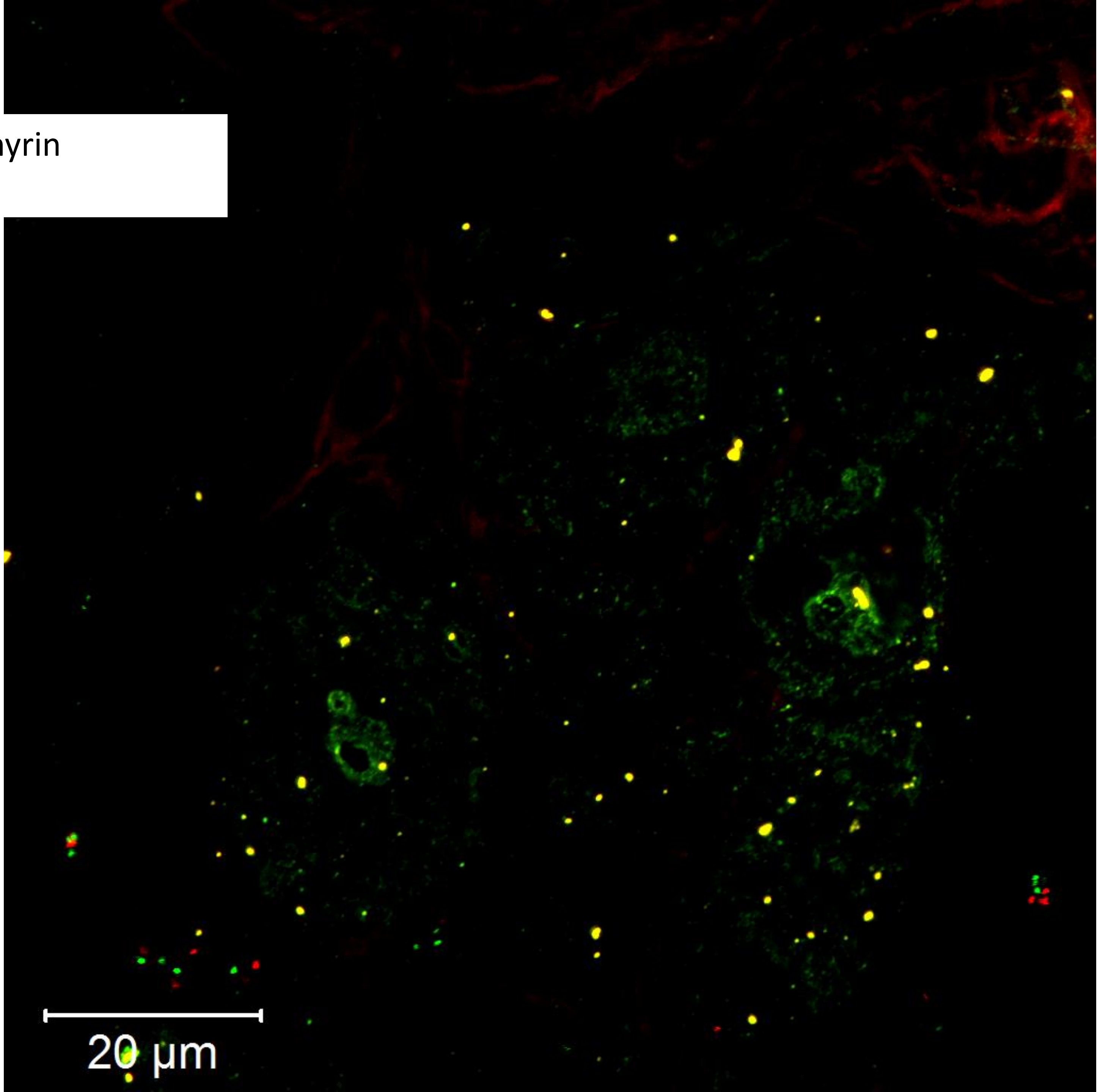

N11-4, GlyRa3 & Gephyrin & NeuN
2016-8-23

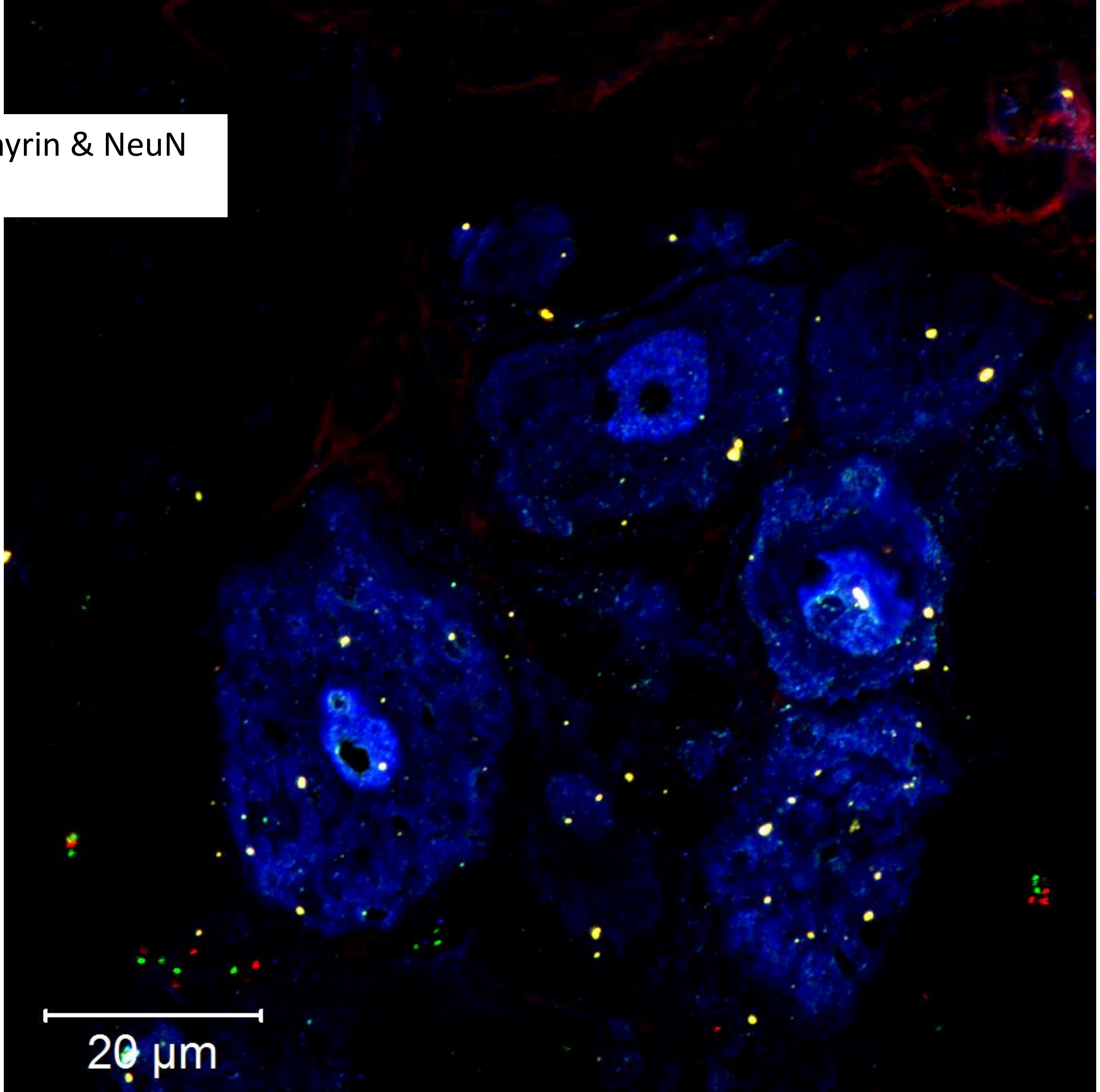

N11-4, GlyRa3 & NeuN
2016-8-23

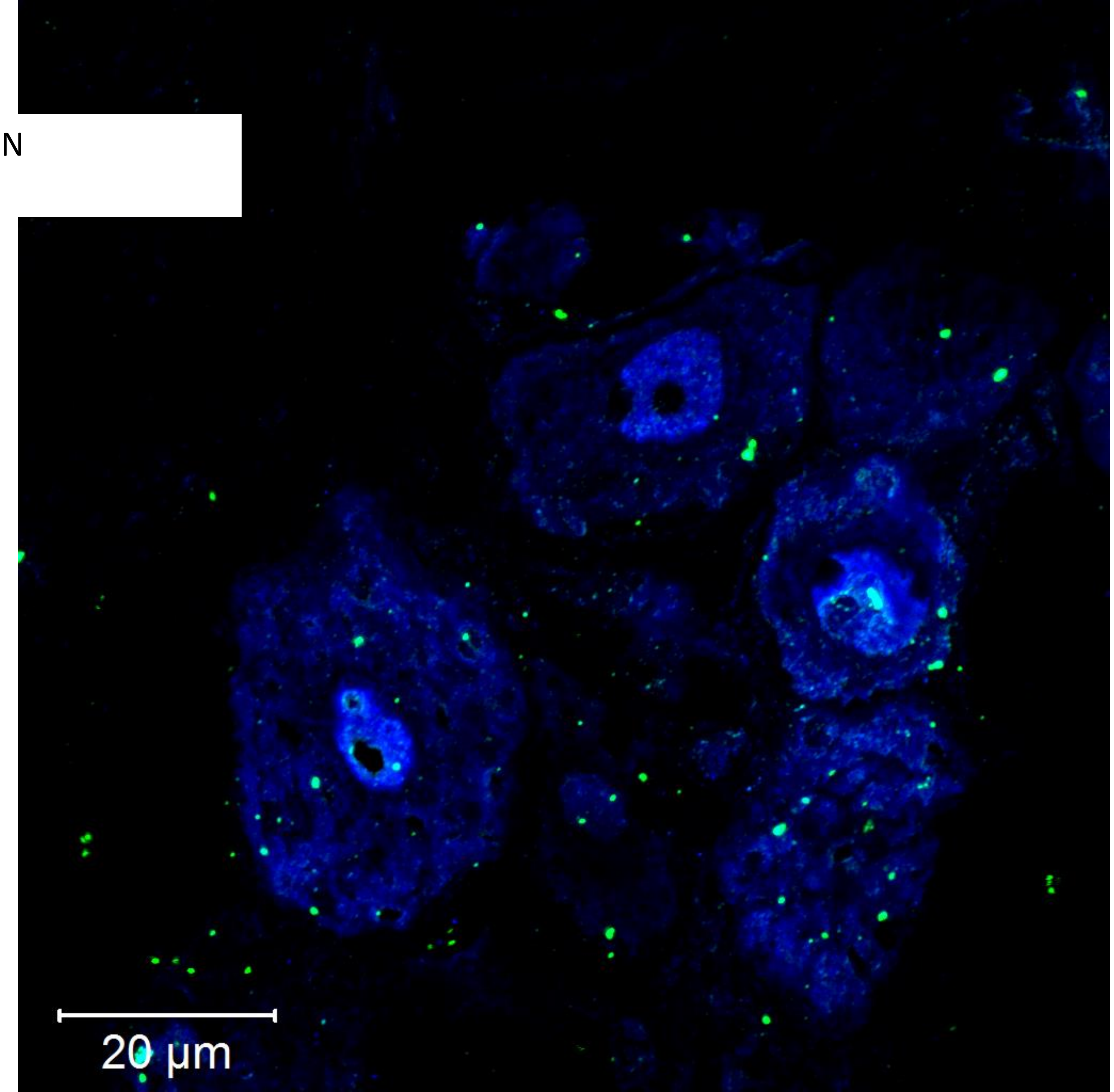

N11-4, NeuN
2016-8-23

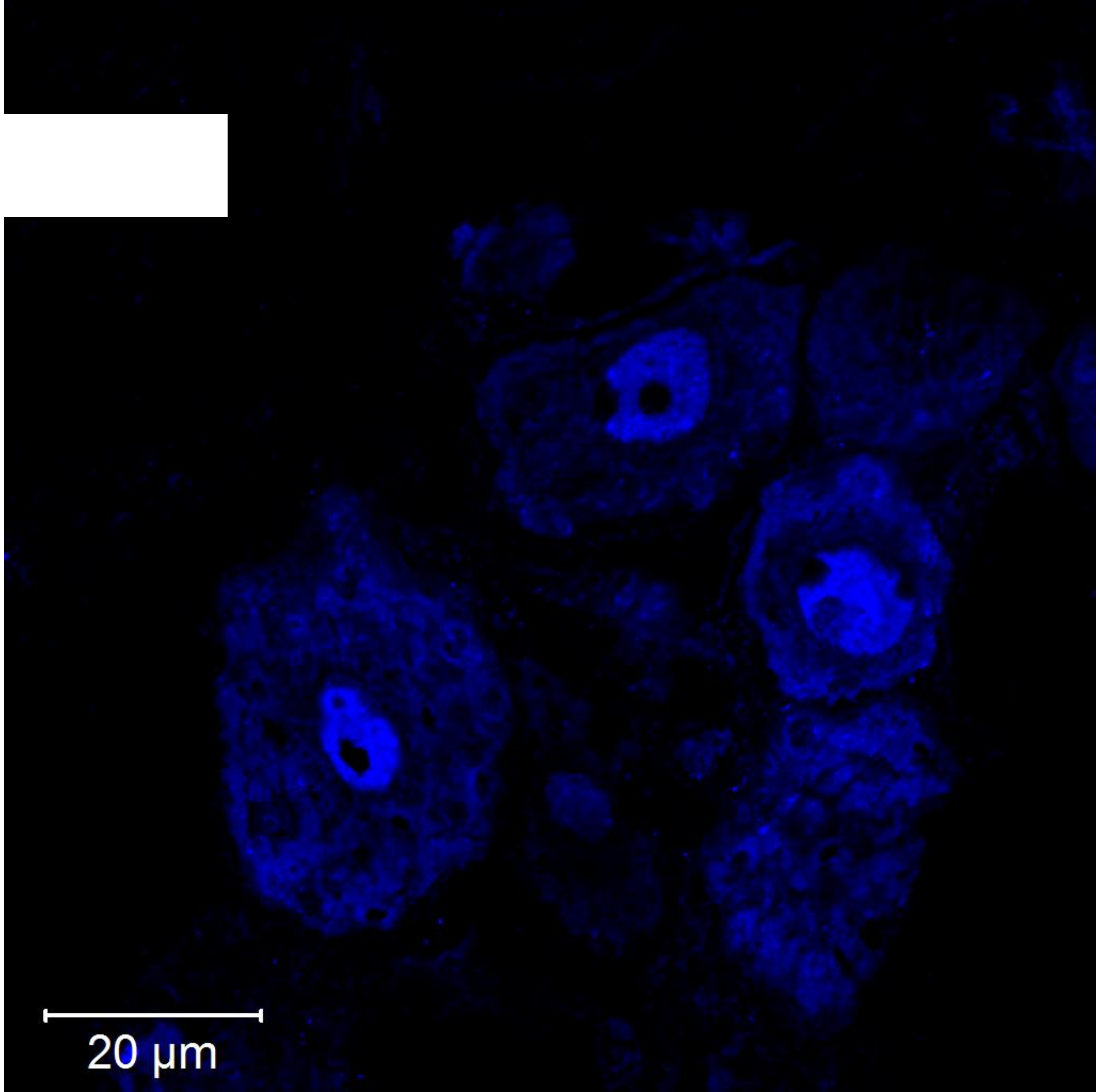

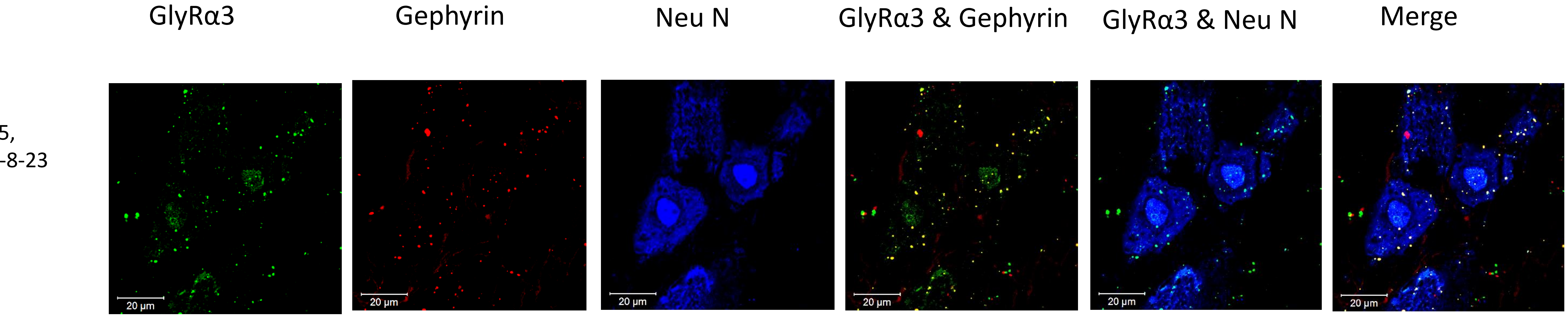
GlyRα3
Gephyrin
Neu N
GlyRα3 & Gephyrin
GlyRα3 & Neu N
Merge
5,
-8-23
20 µm

N11-5, Gephyrin
2016-8-23

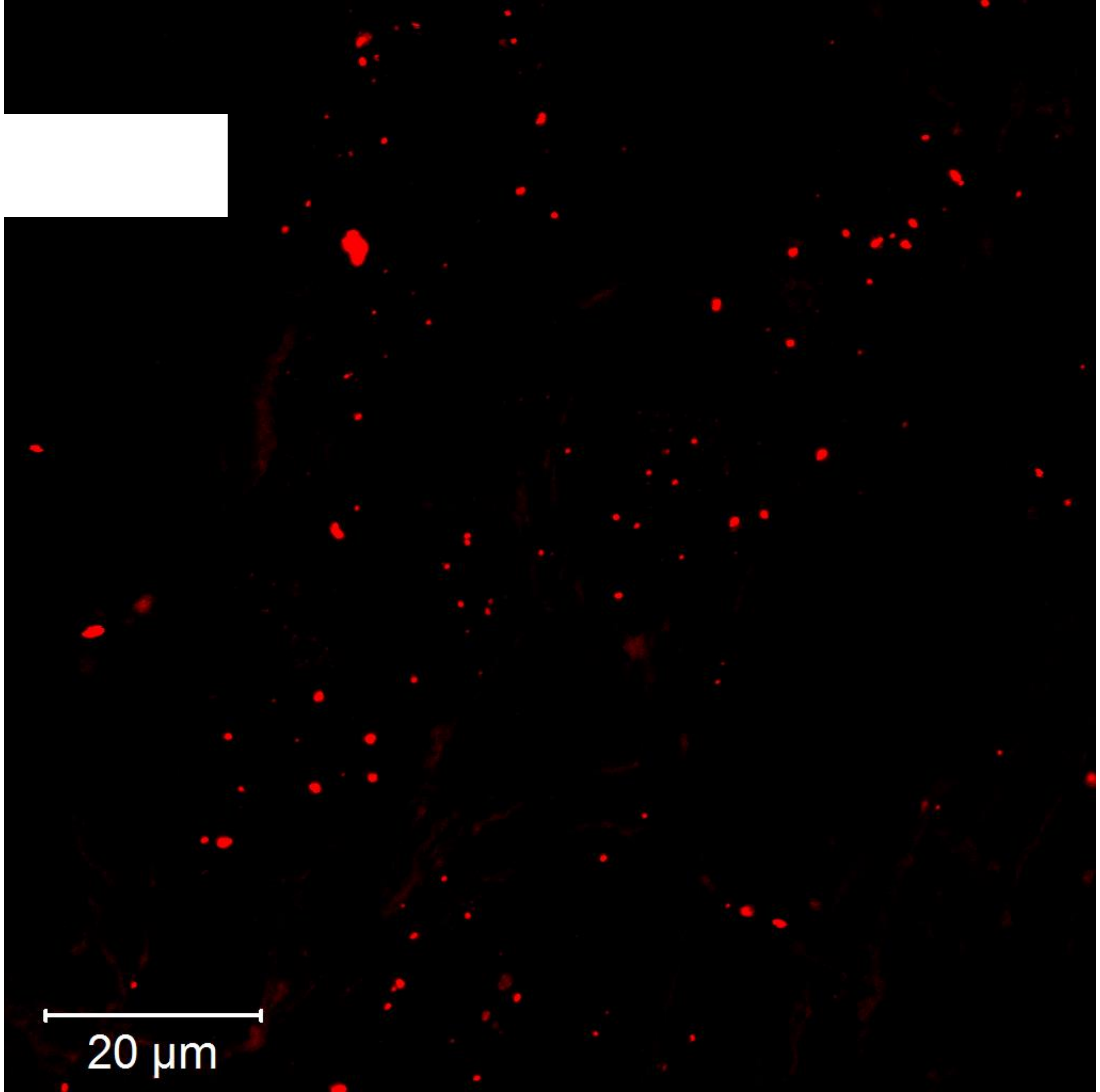

N11-5, Gephyrin & NeuN
2016-8-23

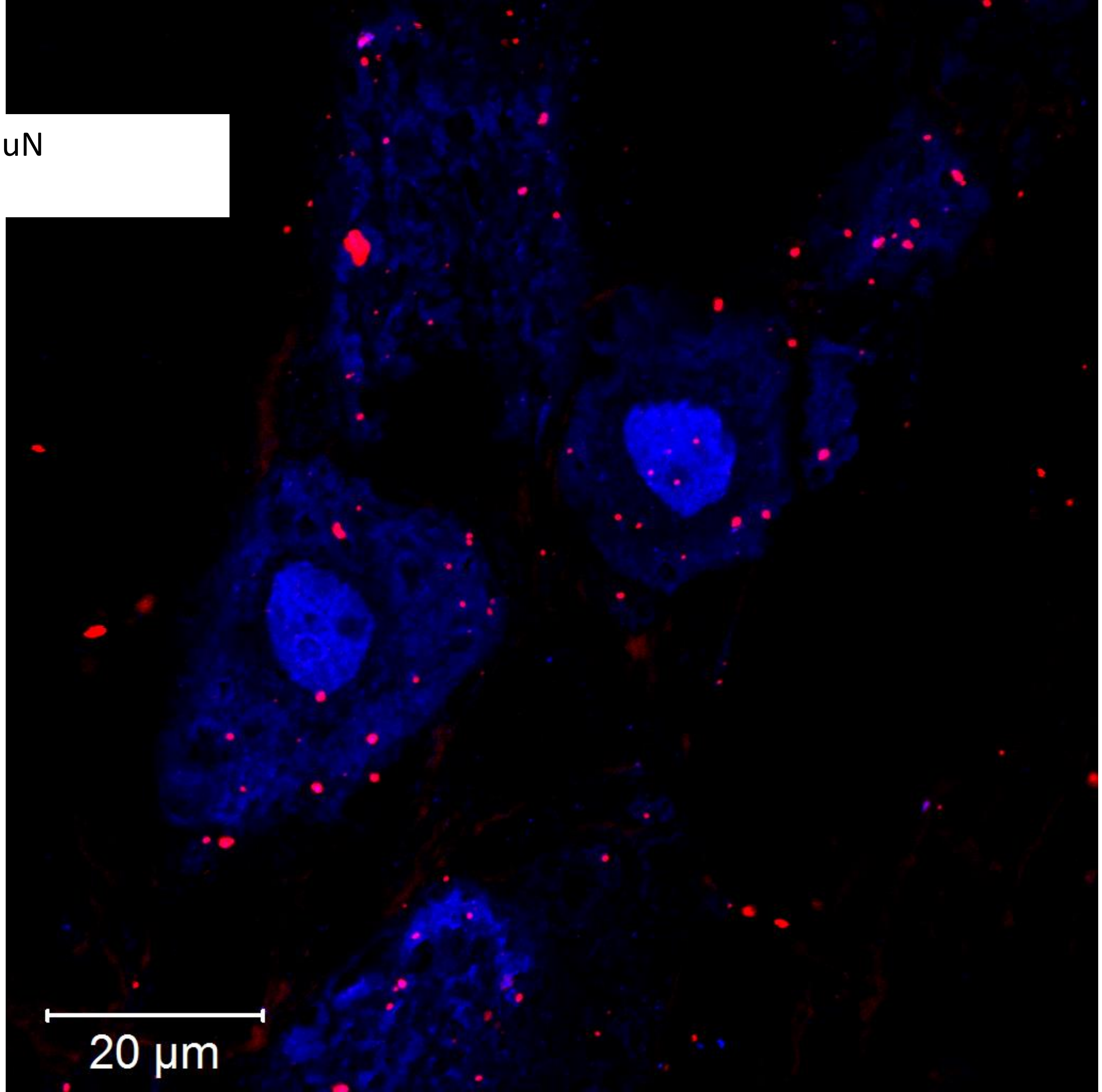

N11-5, GlyRa3
2016-8-23

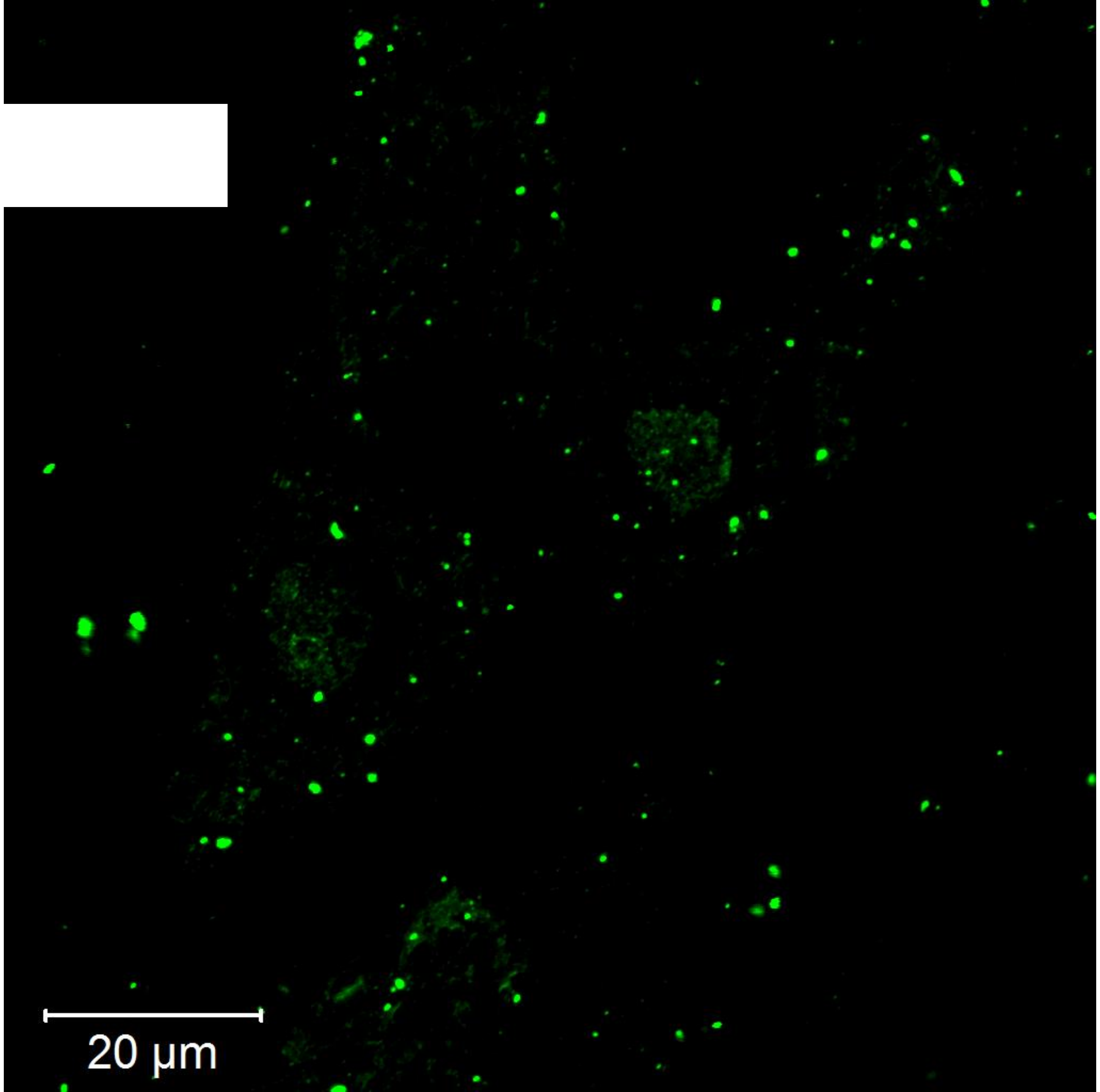

N11-5, GlyRa3 & Gephyrin
2016-8-23

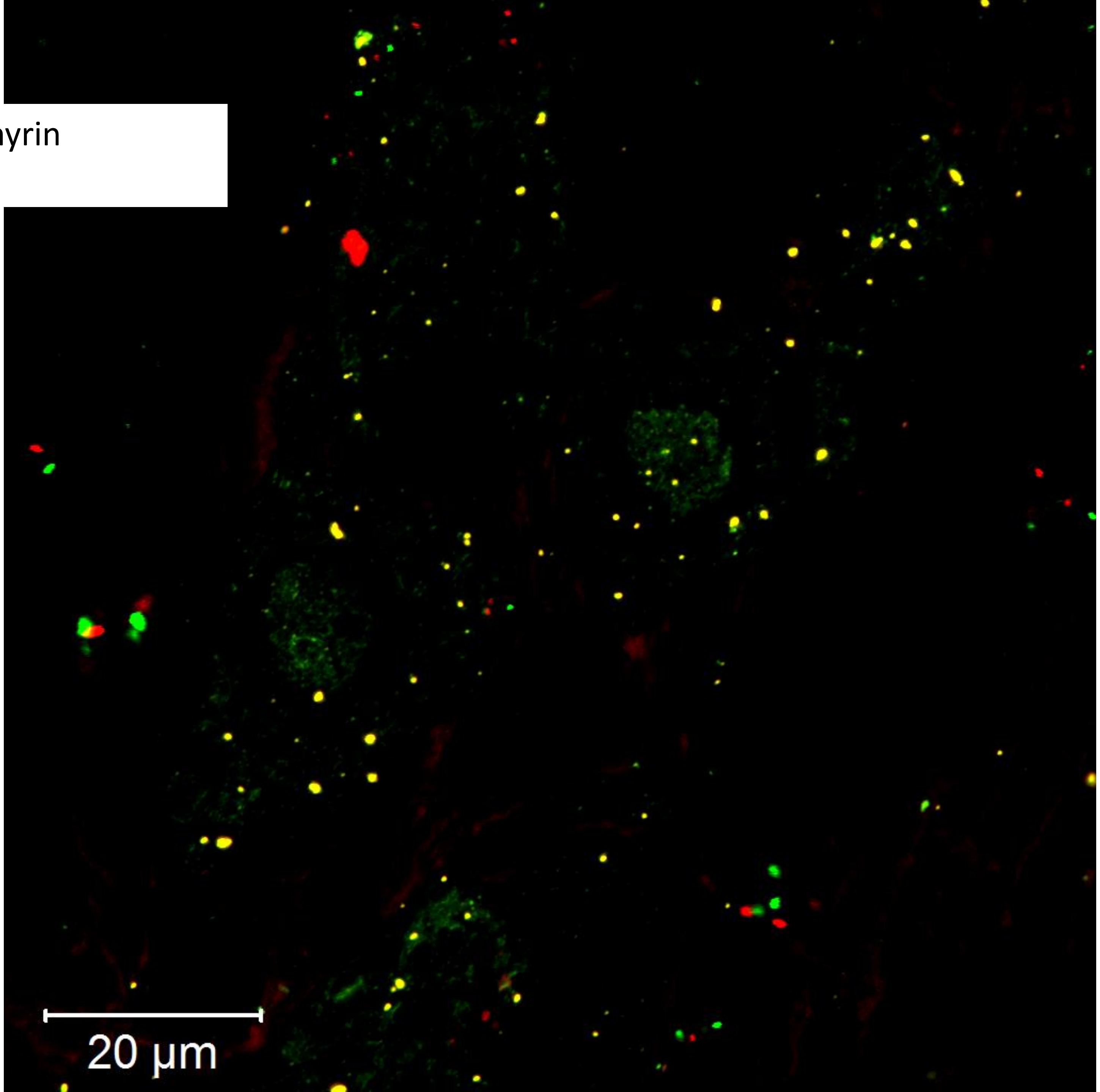

N11-5, GlyRa3 & Gephyrin & NeuN
2016-8-23

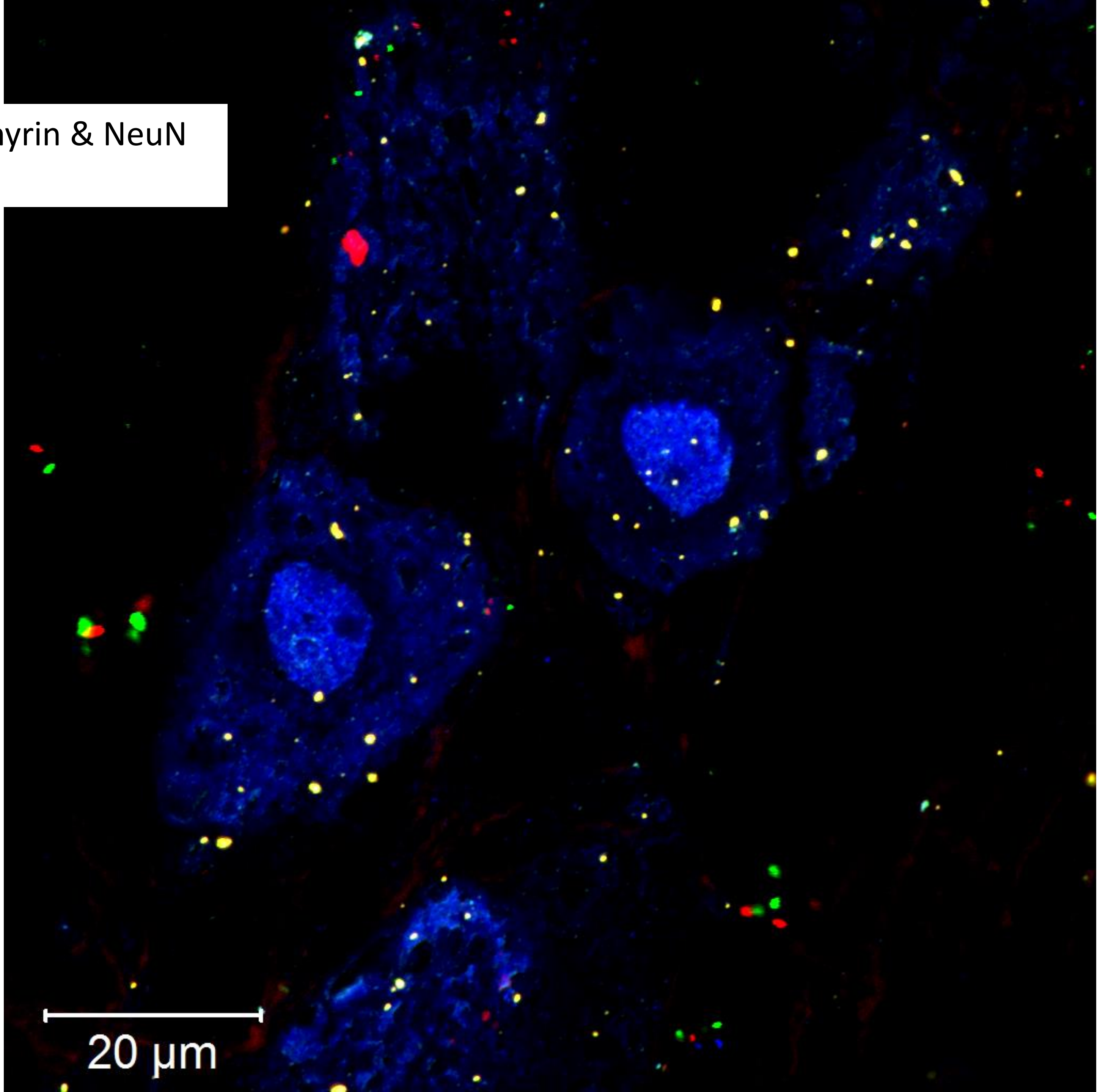

N11-5, GlyRa3 & NeuN
2016-8-23

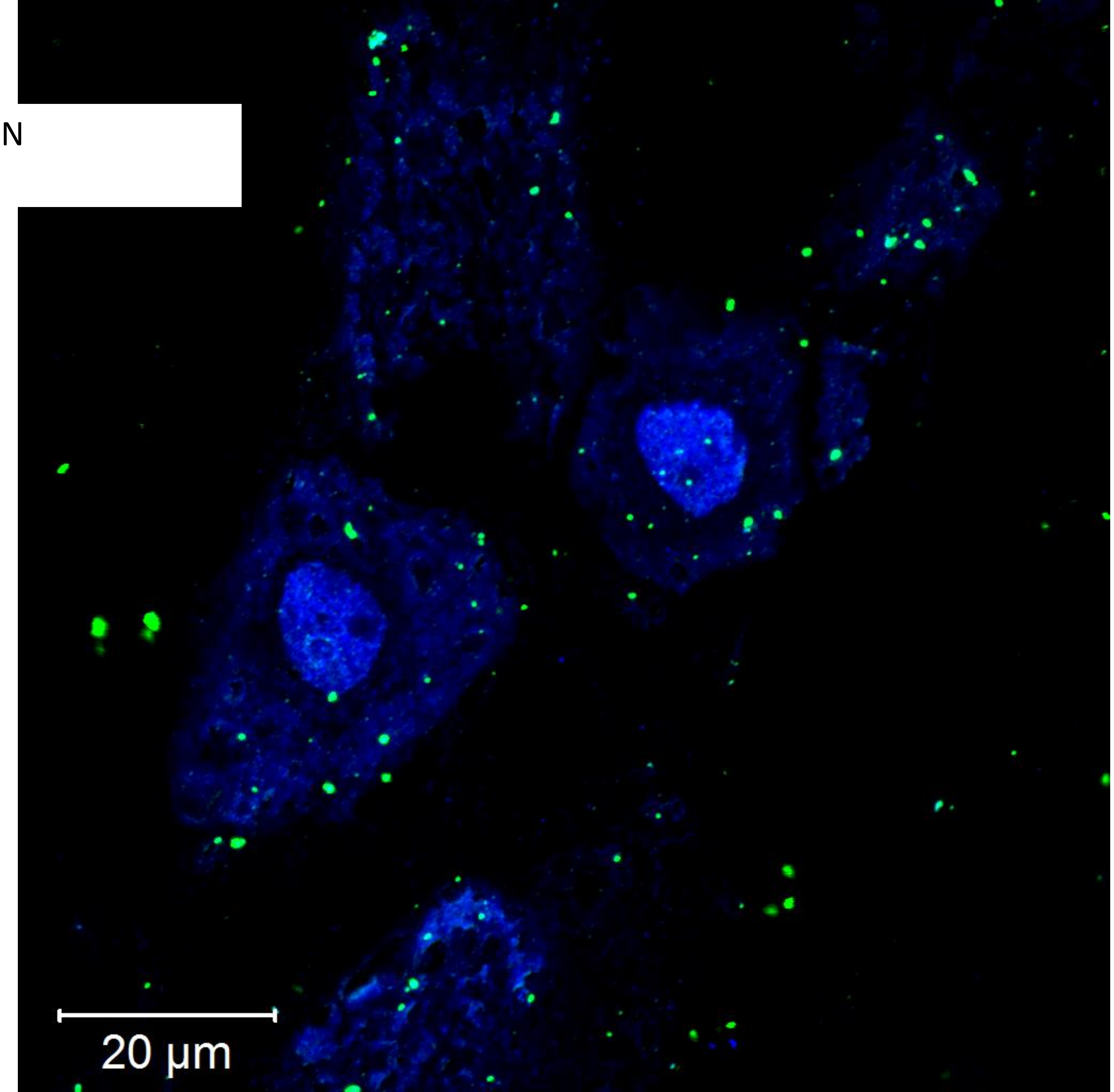

N11-5, NeuN
2016-8-23

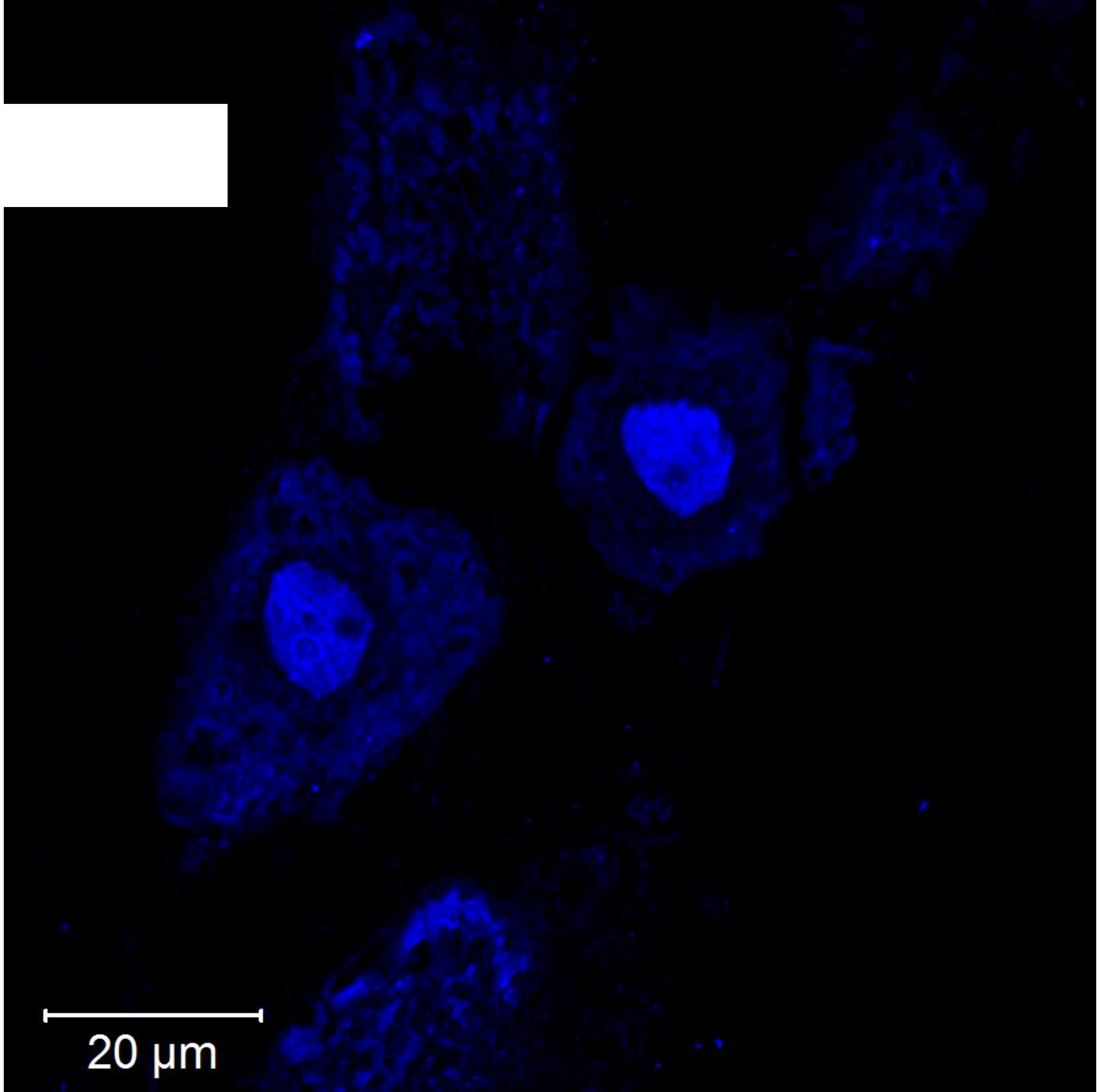

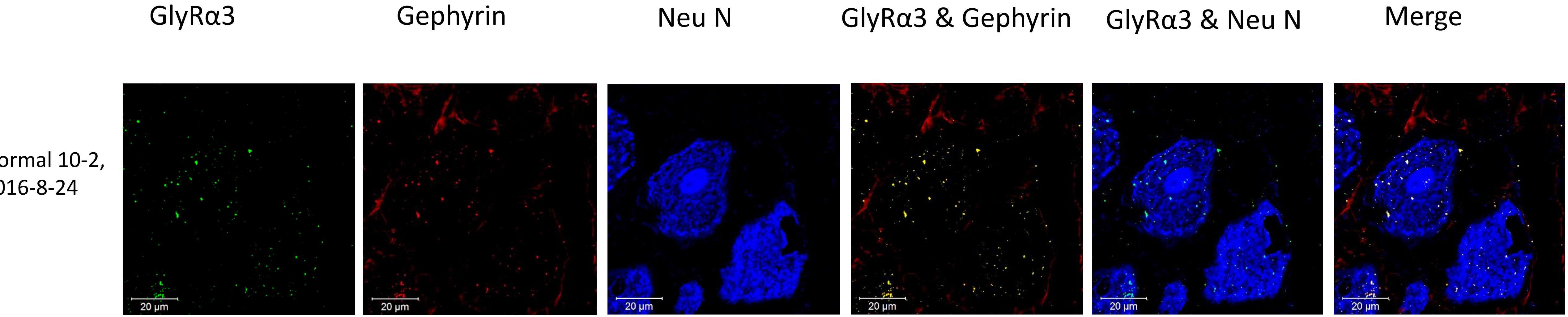
GlyRα3
Gephyrin
Neu N
GlyRα3 & Gephyrin
GlyRα3 & Neu N
Merge
ormal 10-2,
016-8-24
20 μm

Normal 10-2, Gephyrin
2016-8-24

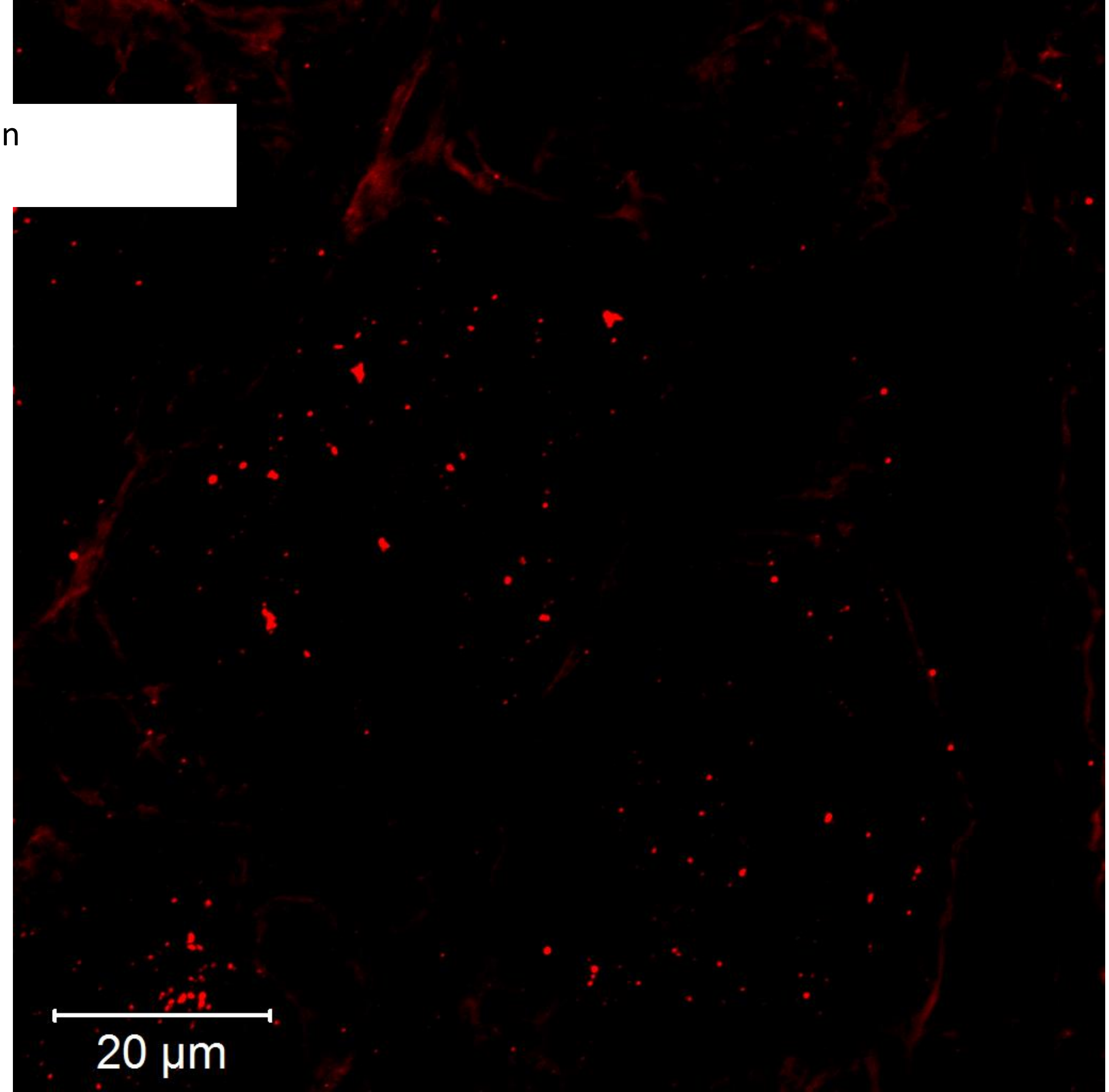

Normal 10-2, Gephyrin & NeuN
2016-8-24

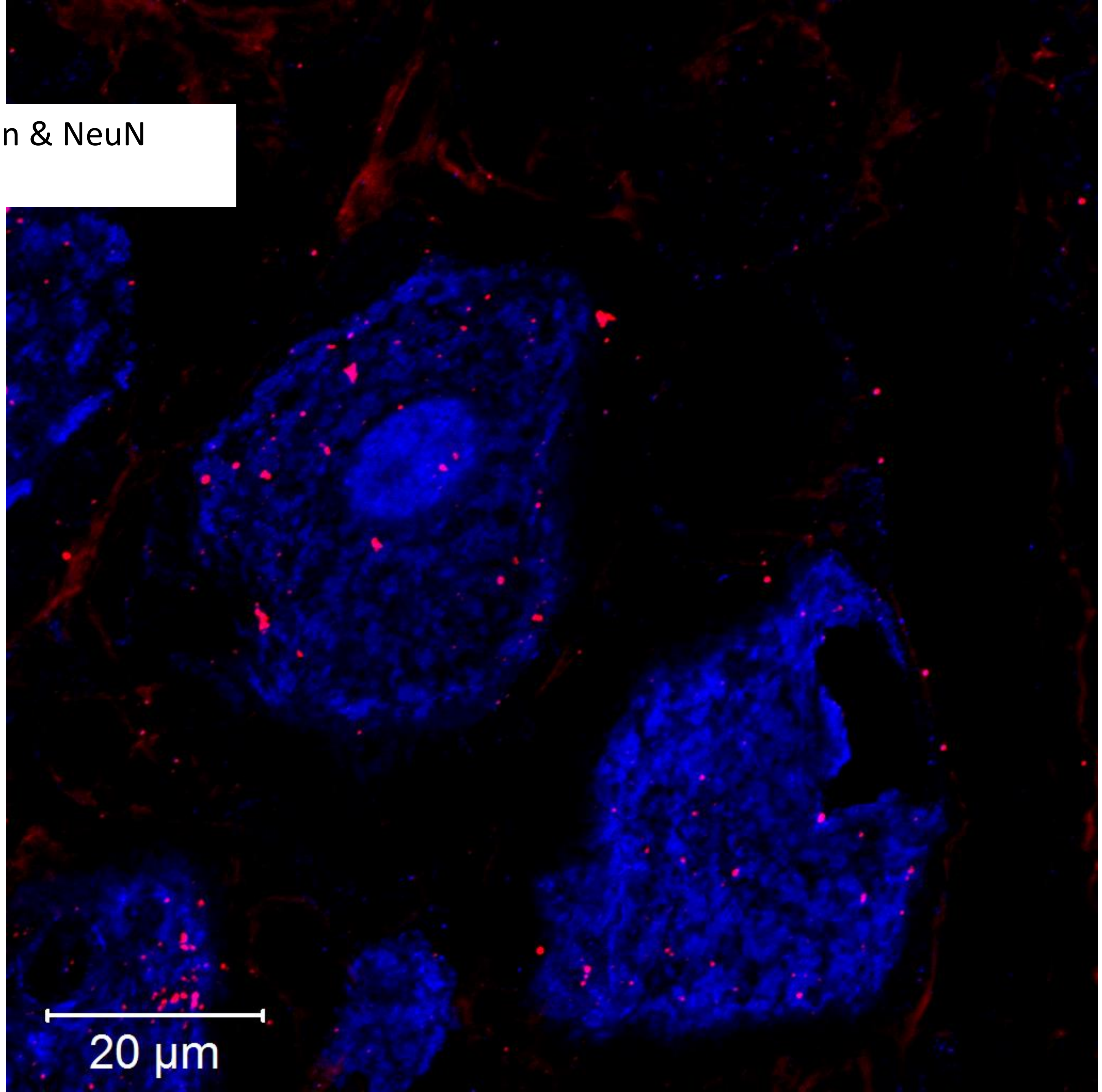

Normal 10-2, GlyRa3
2016-8-24

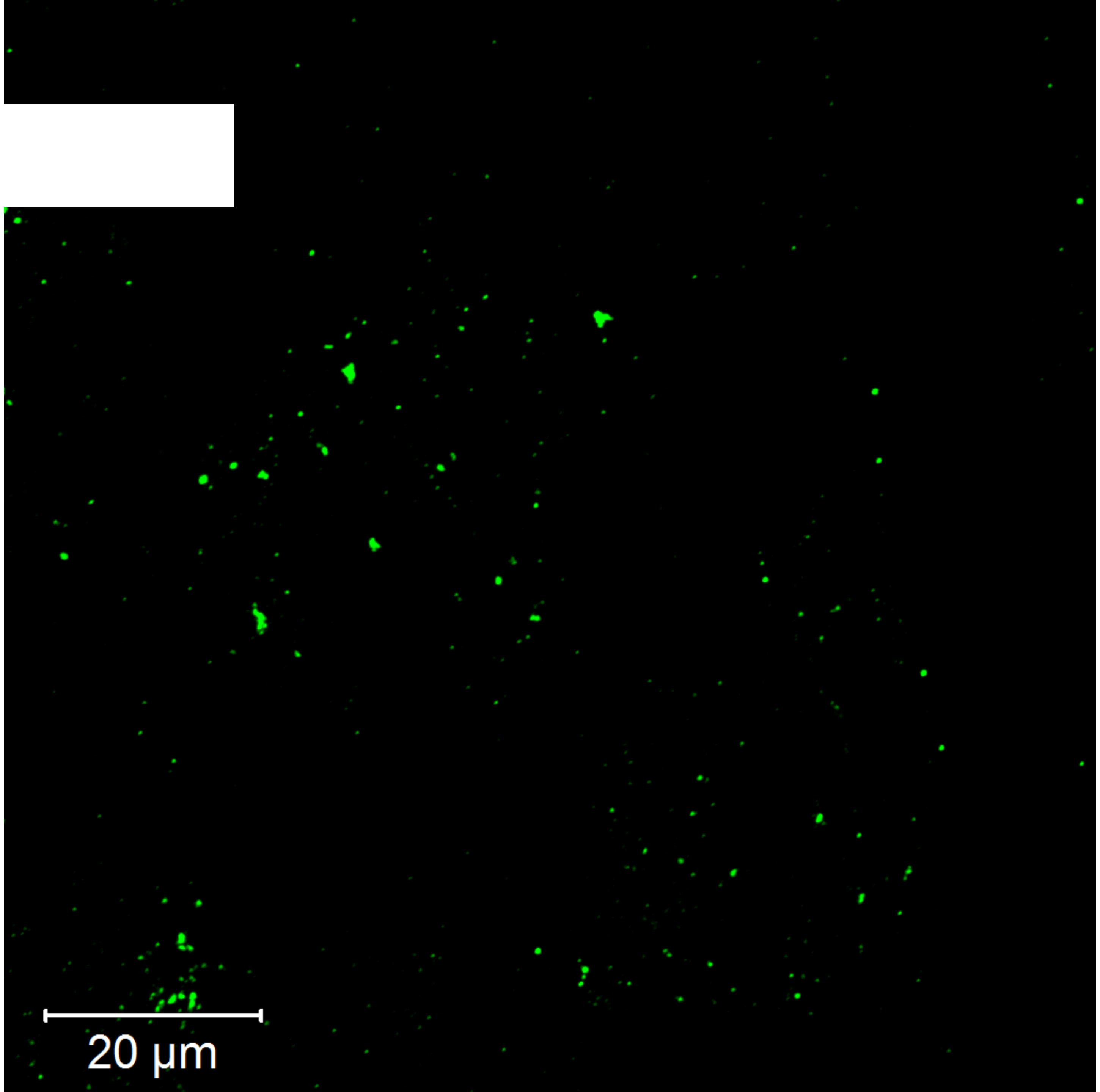

Normal 10-2, GlyRa3 & Gephyrin
2016-8-24

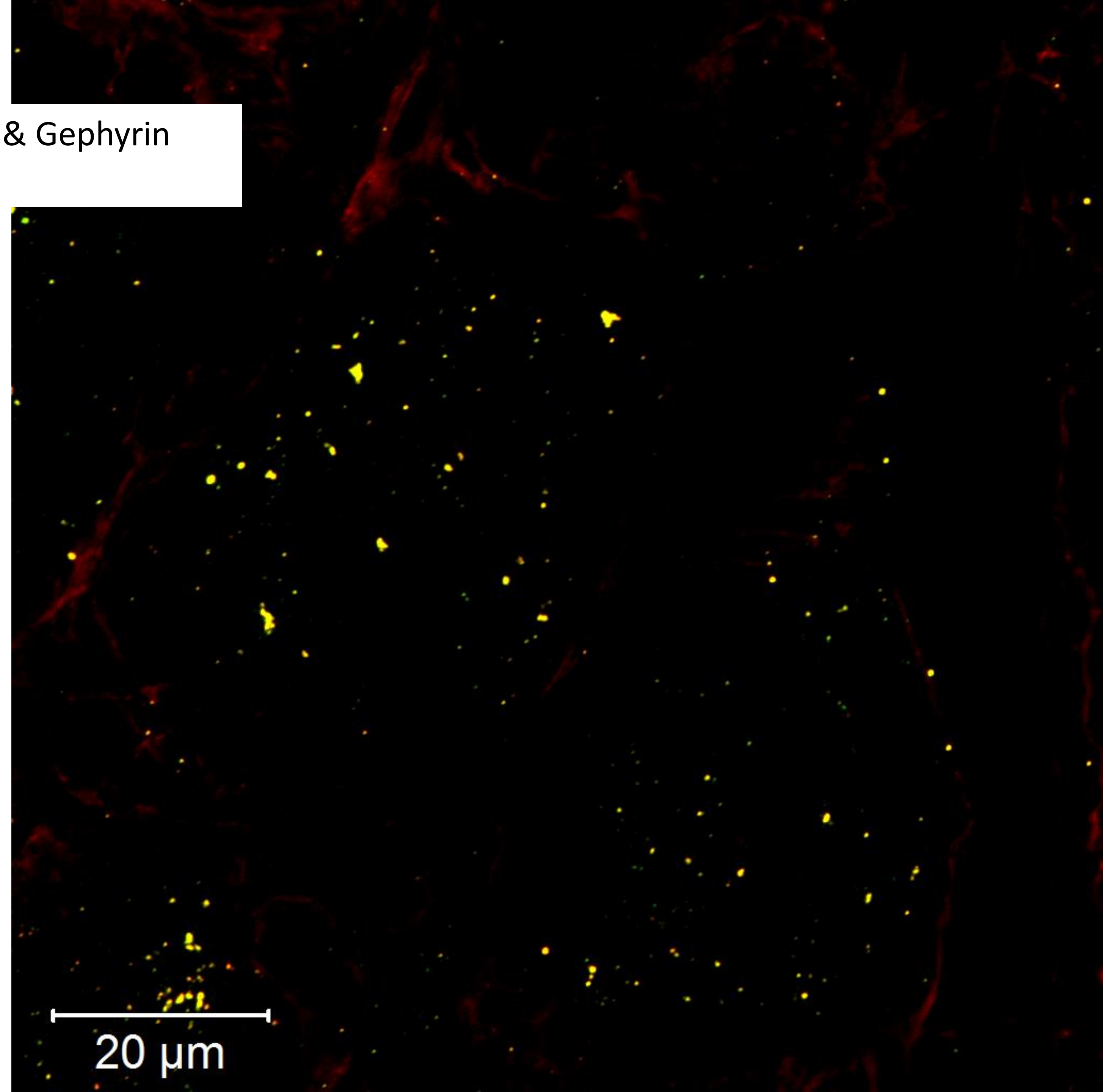

Normal 10-2, GlyRa3 & Gephyrin & NeuN
2016-8-24

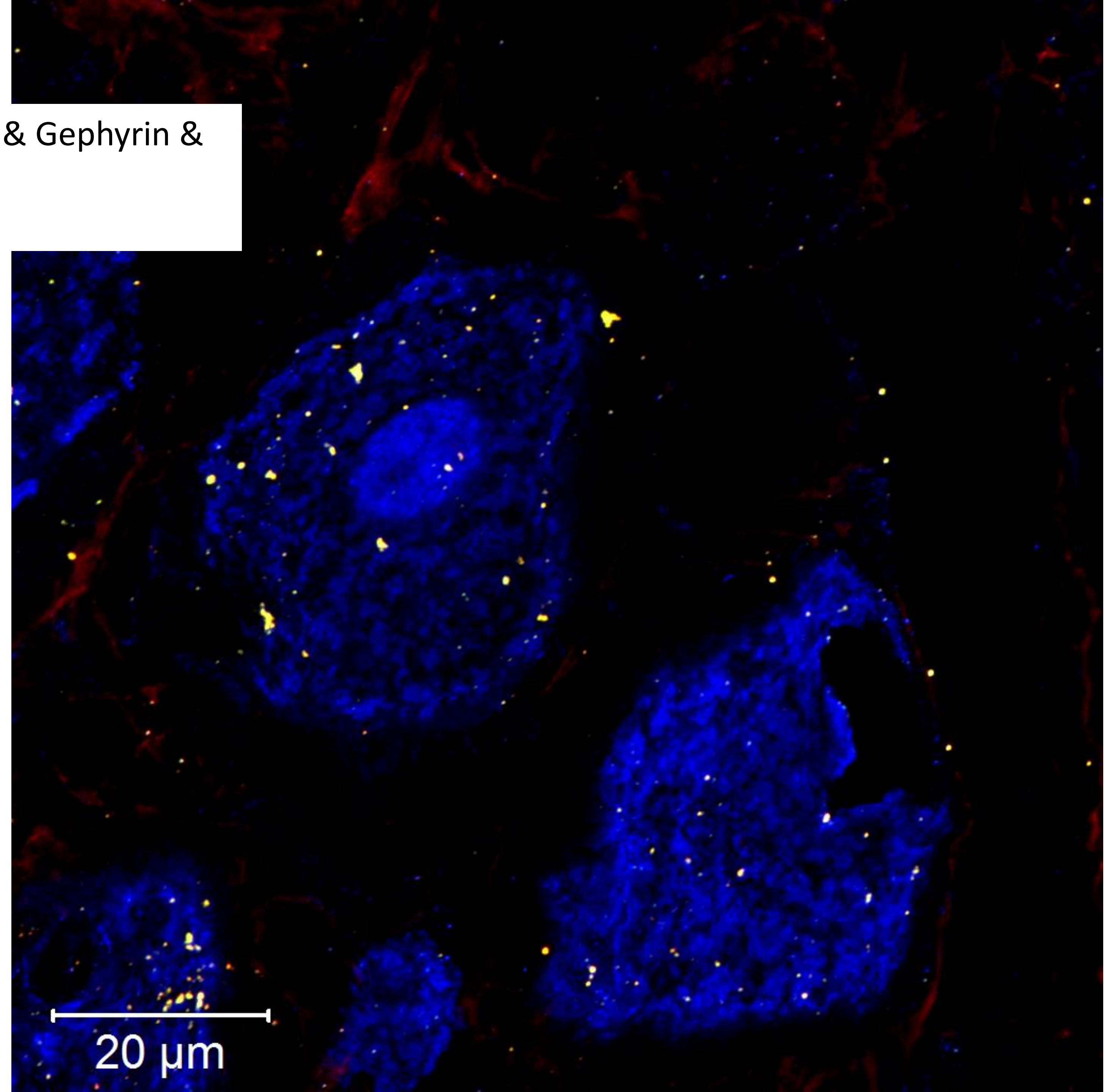

Normal 10-2, GlyRa3 & NeuN
2016-8-24

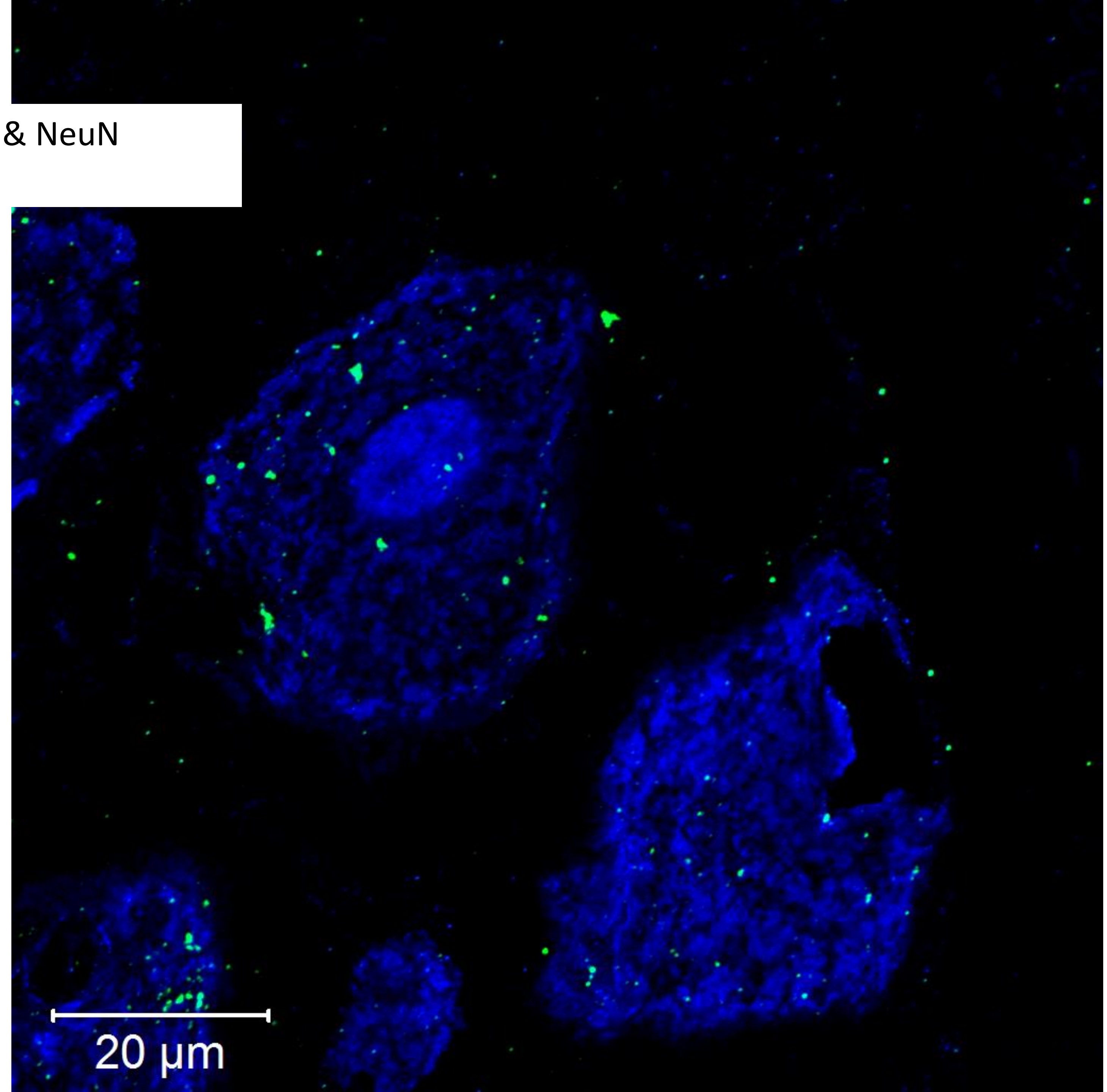

Normal 10-2, NeuN
2016-8-24

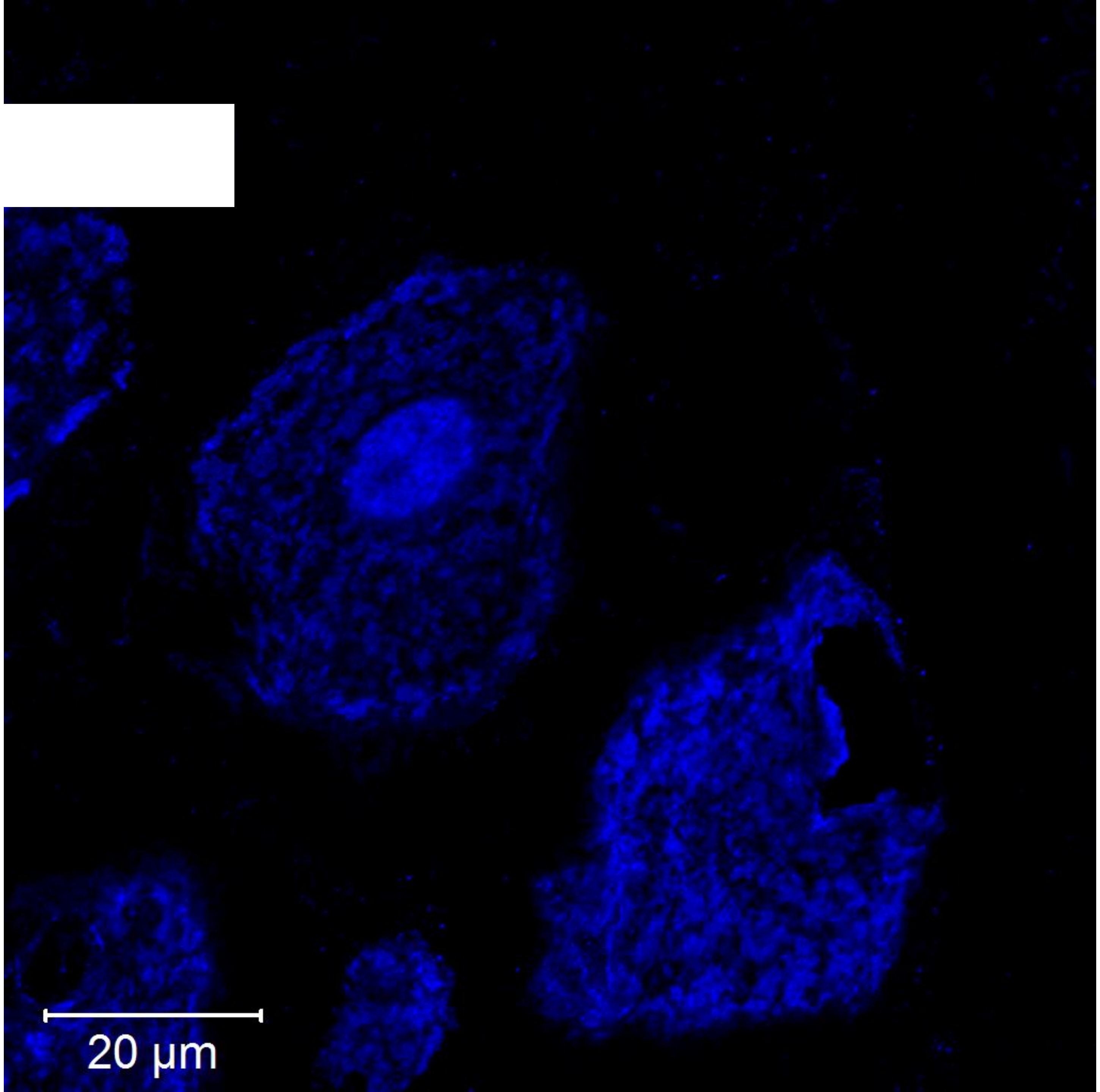

Normal 10-3, Gephyrin
2016-8-24

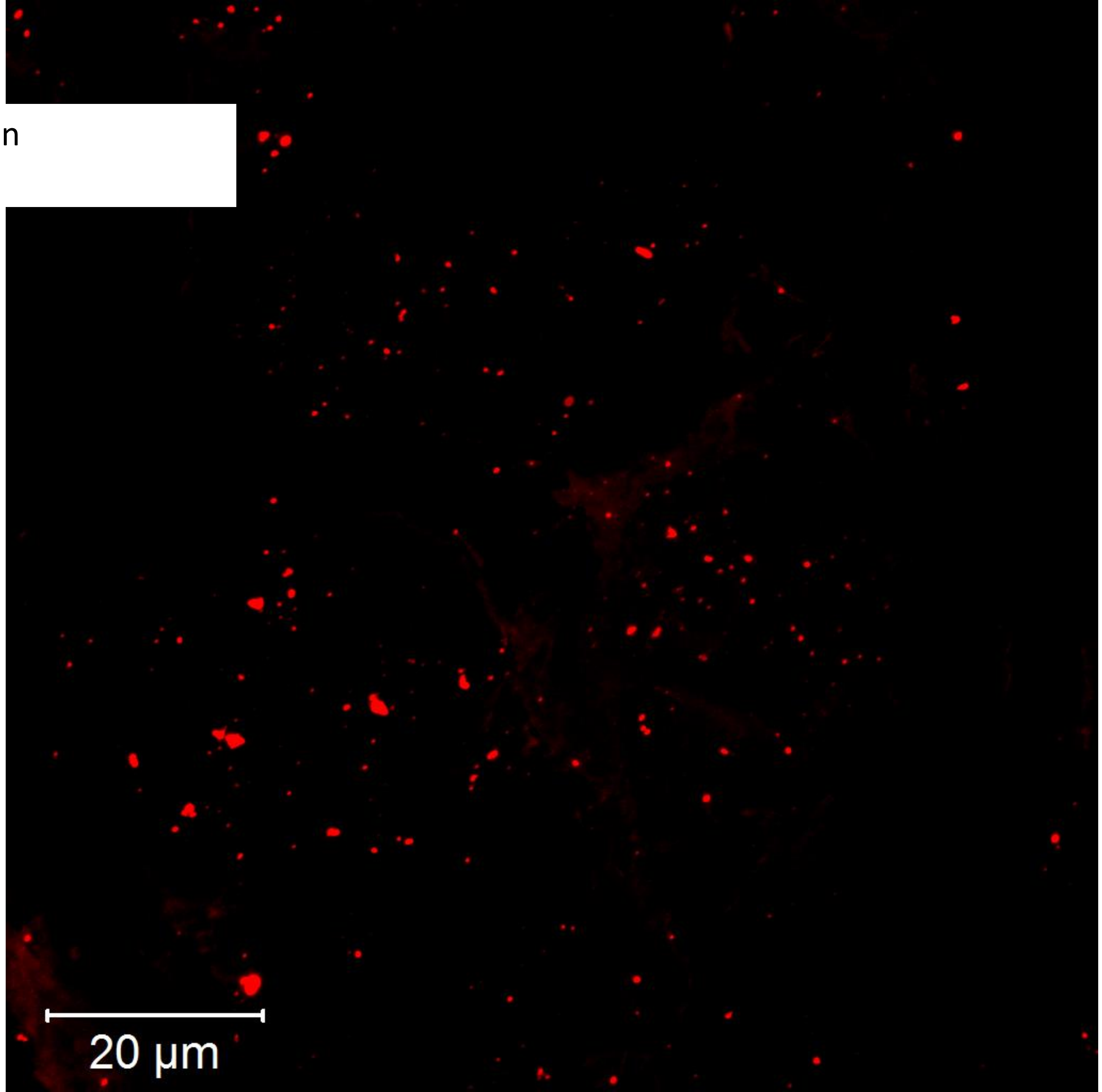

Normal 10-3, Gephyrin & NeuN
2016-8-24

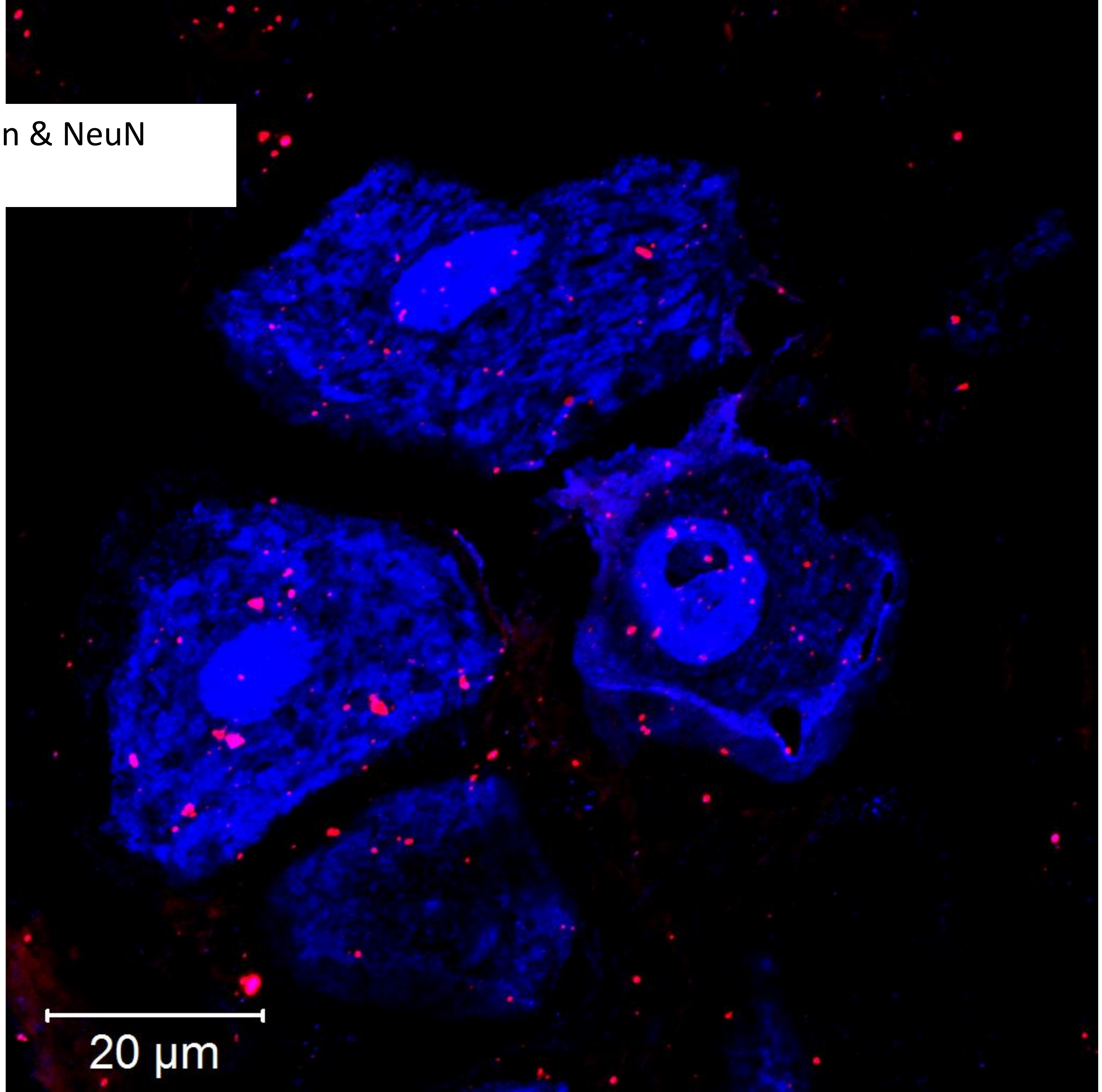

Normal 10-3, GlyRa3
2016-8-24

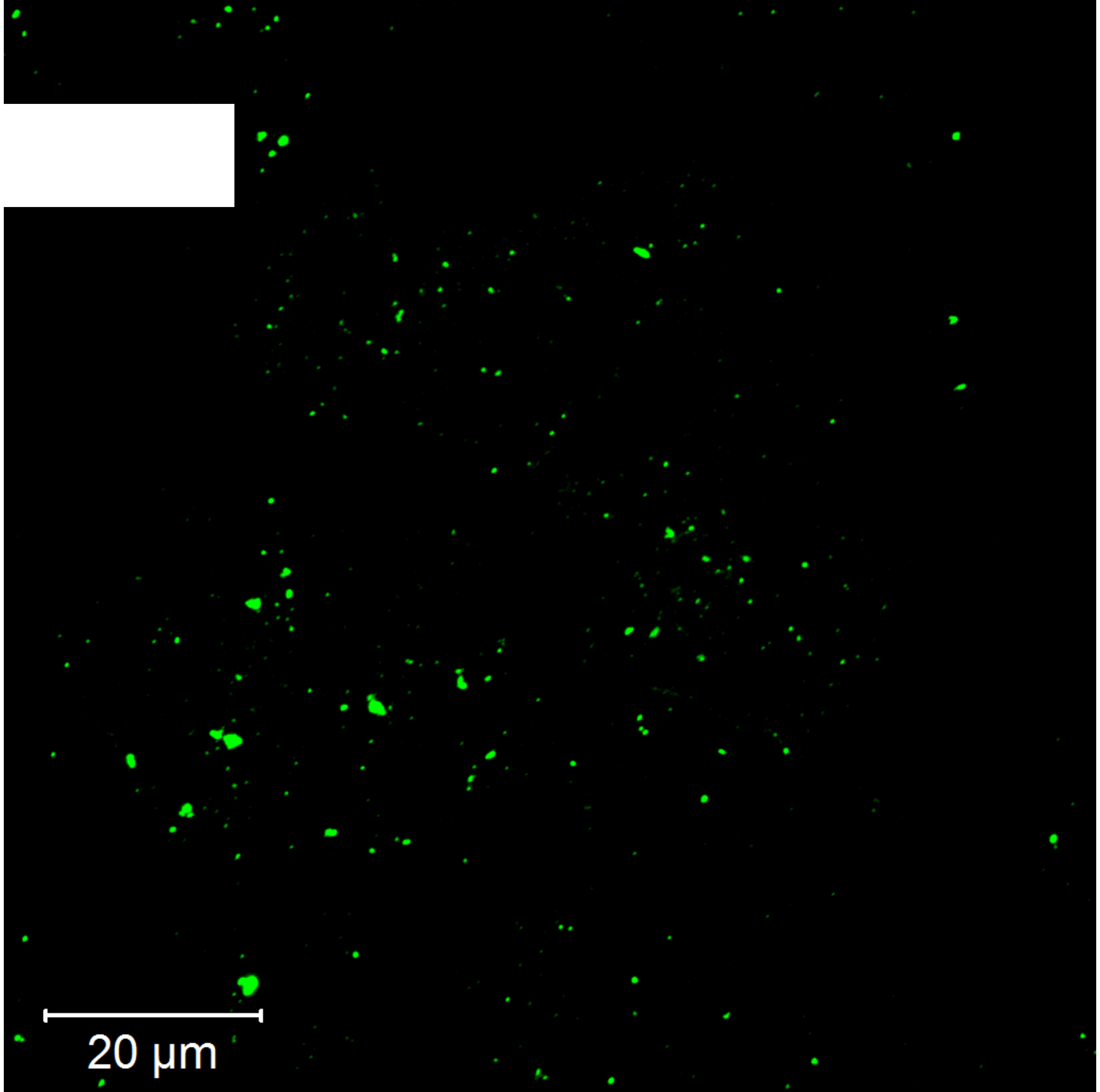

Normal 10-3, GlyRa3 & Gephyrin
2016-8-24

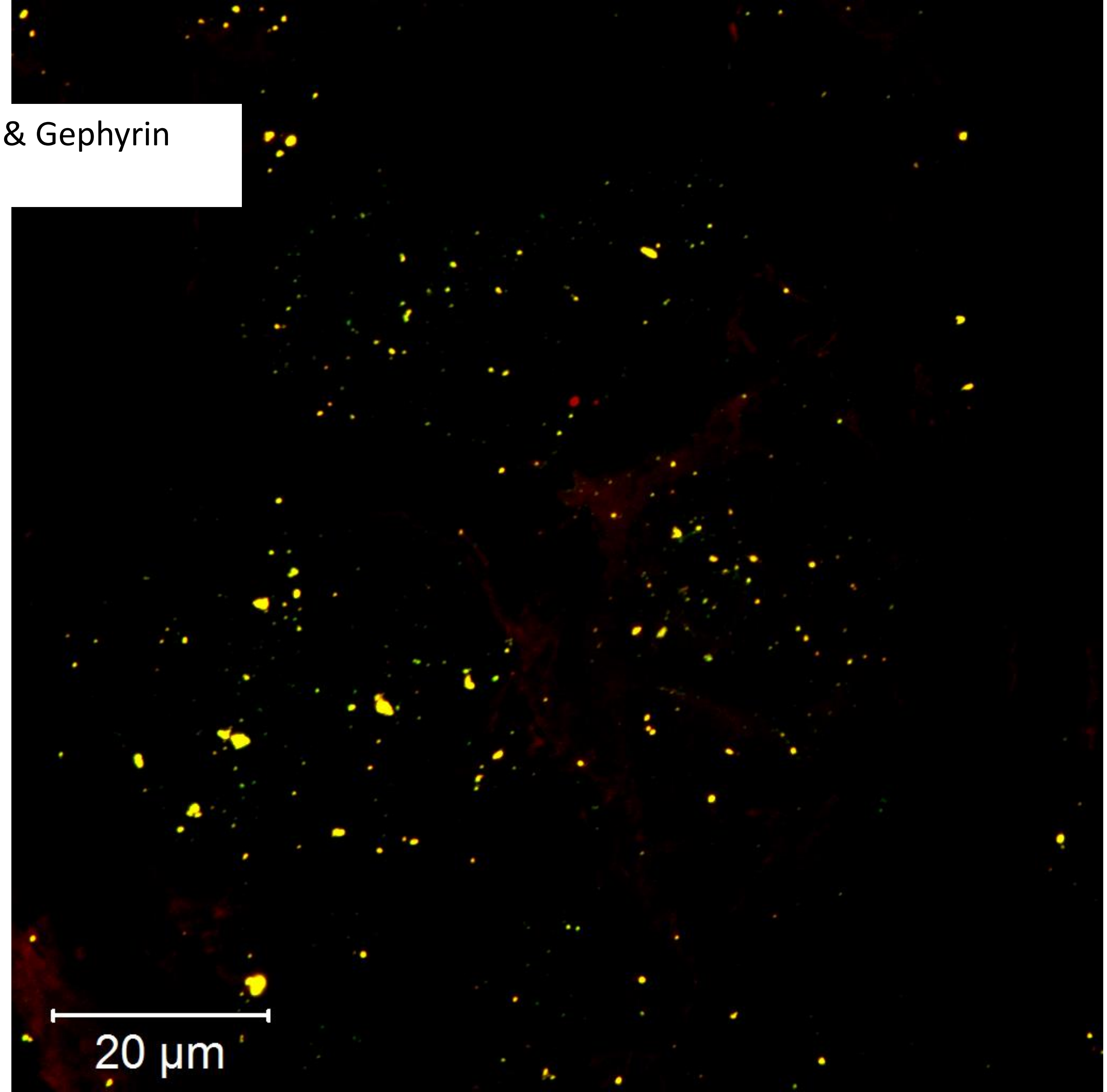

Normal 10-3, GlyRa3 & Gephyrin & NeuN
2016-8-24

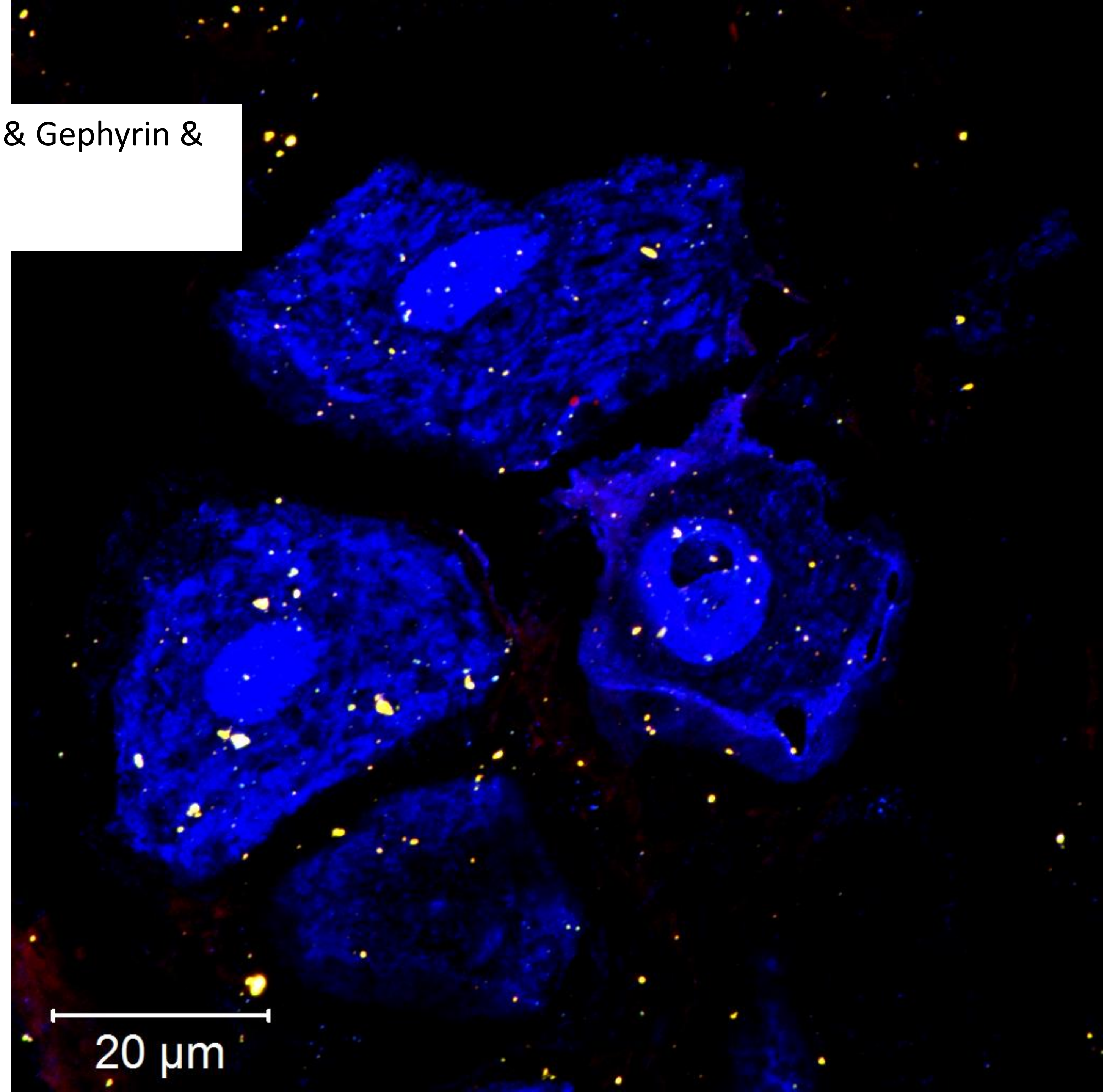

Normal 10-3, GlyRa3 & NeuN
2016-8-24

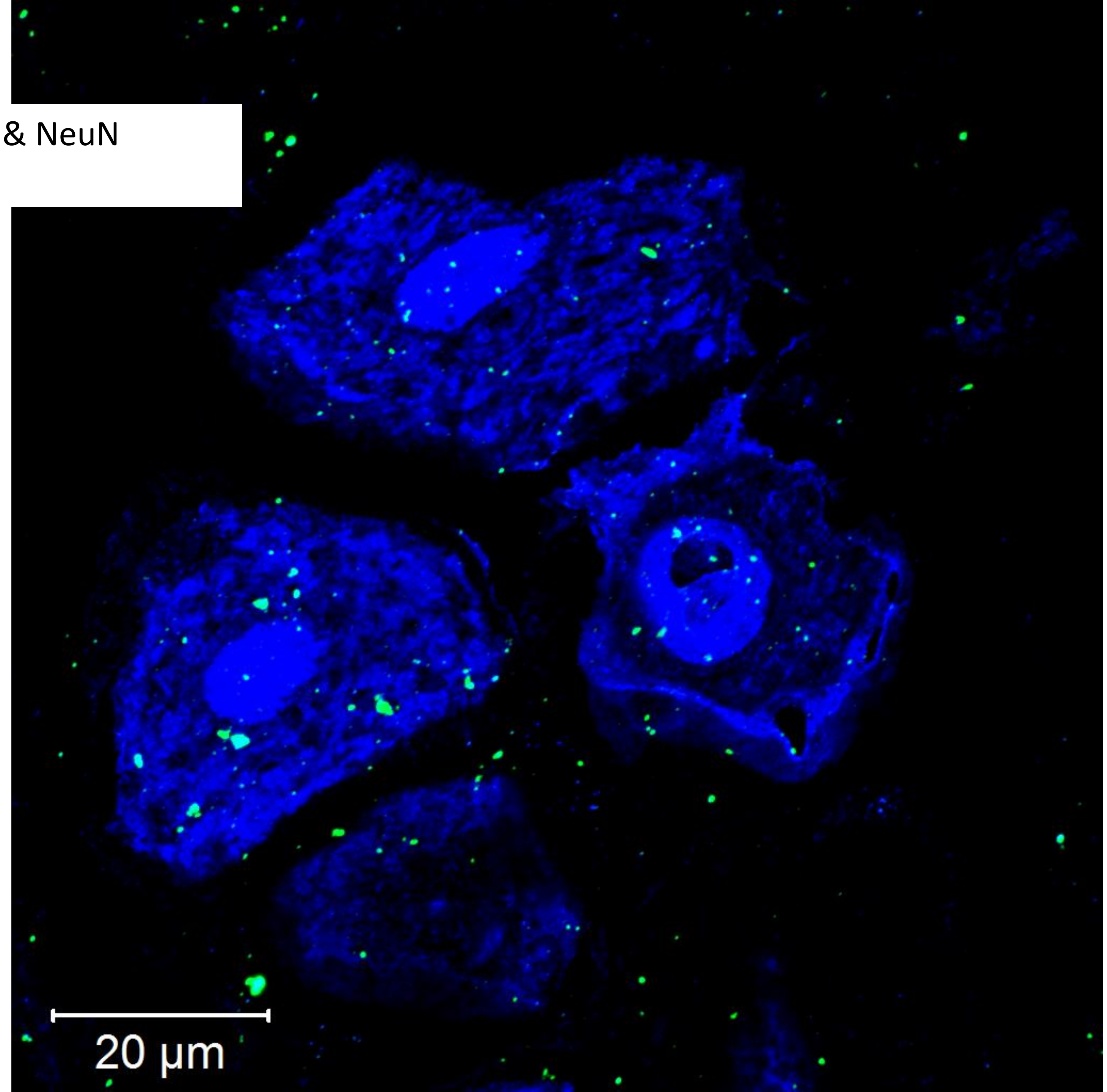

Normal 10-3, NeuN
2016-8-24

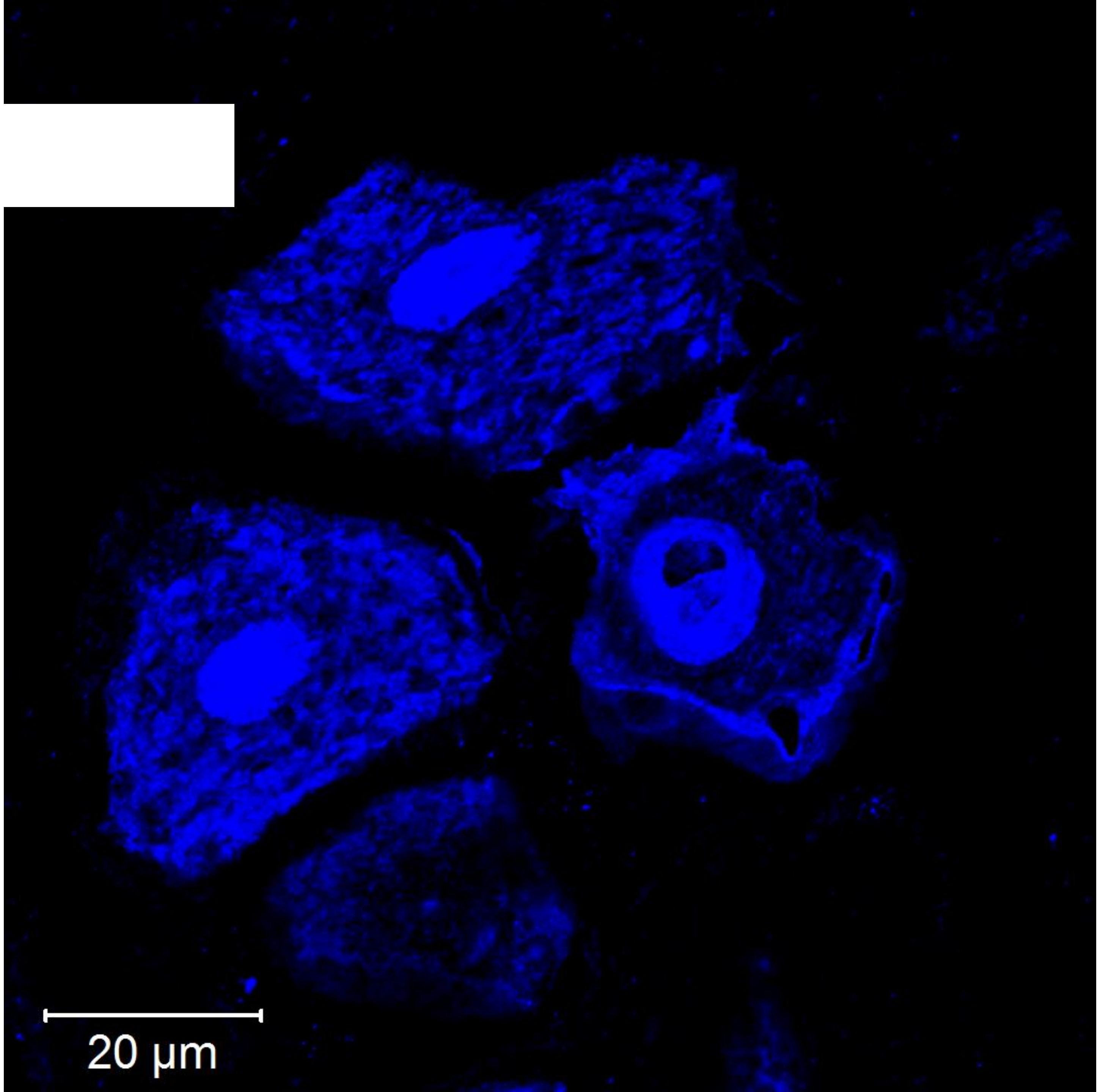

Normal 10-4, gephyrin
2016-8-24

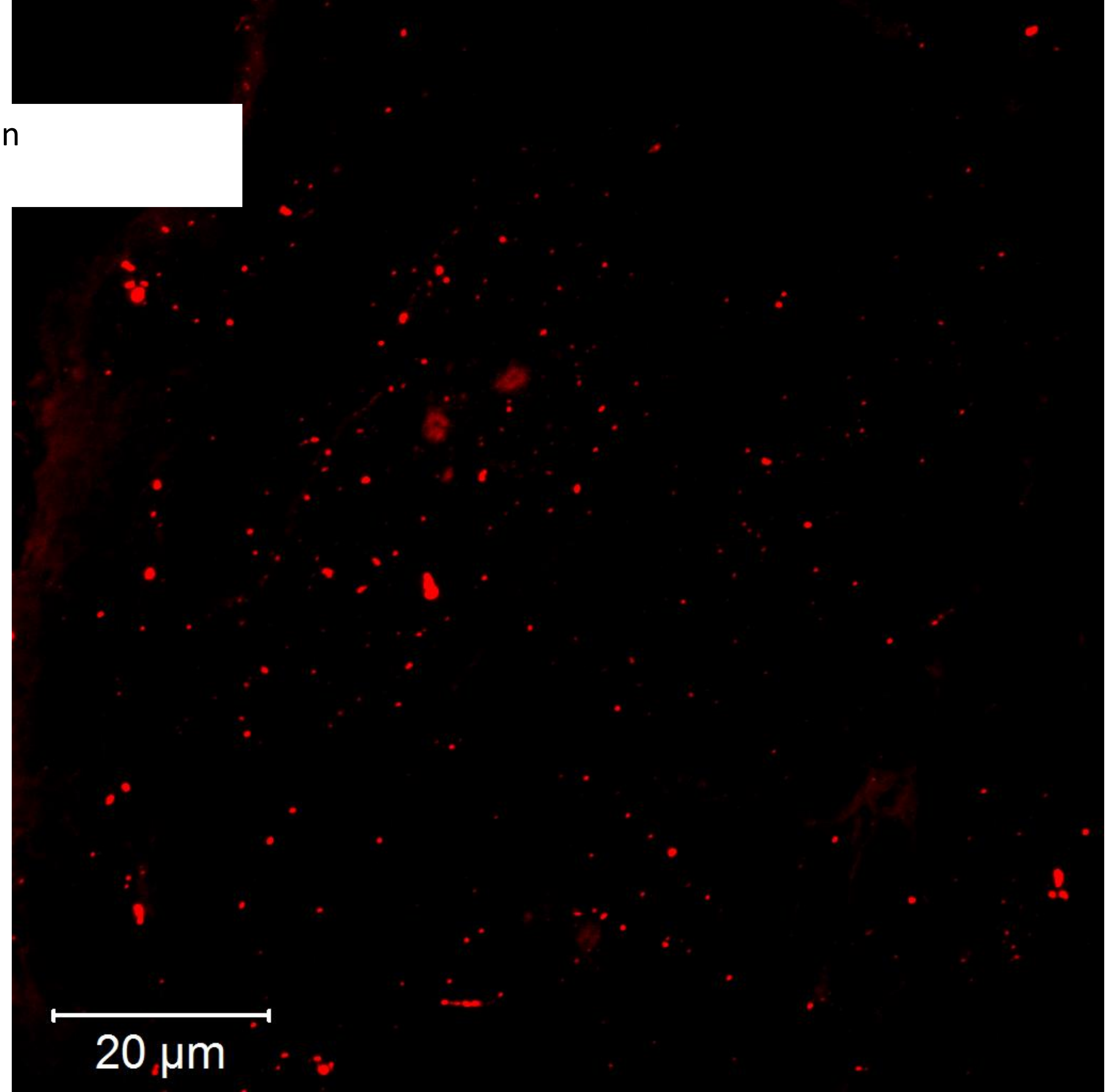

Normal 10-4, gephyrin & NeuN
2016-8-24

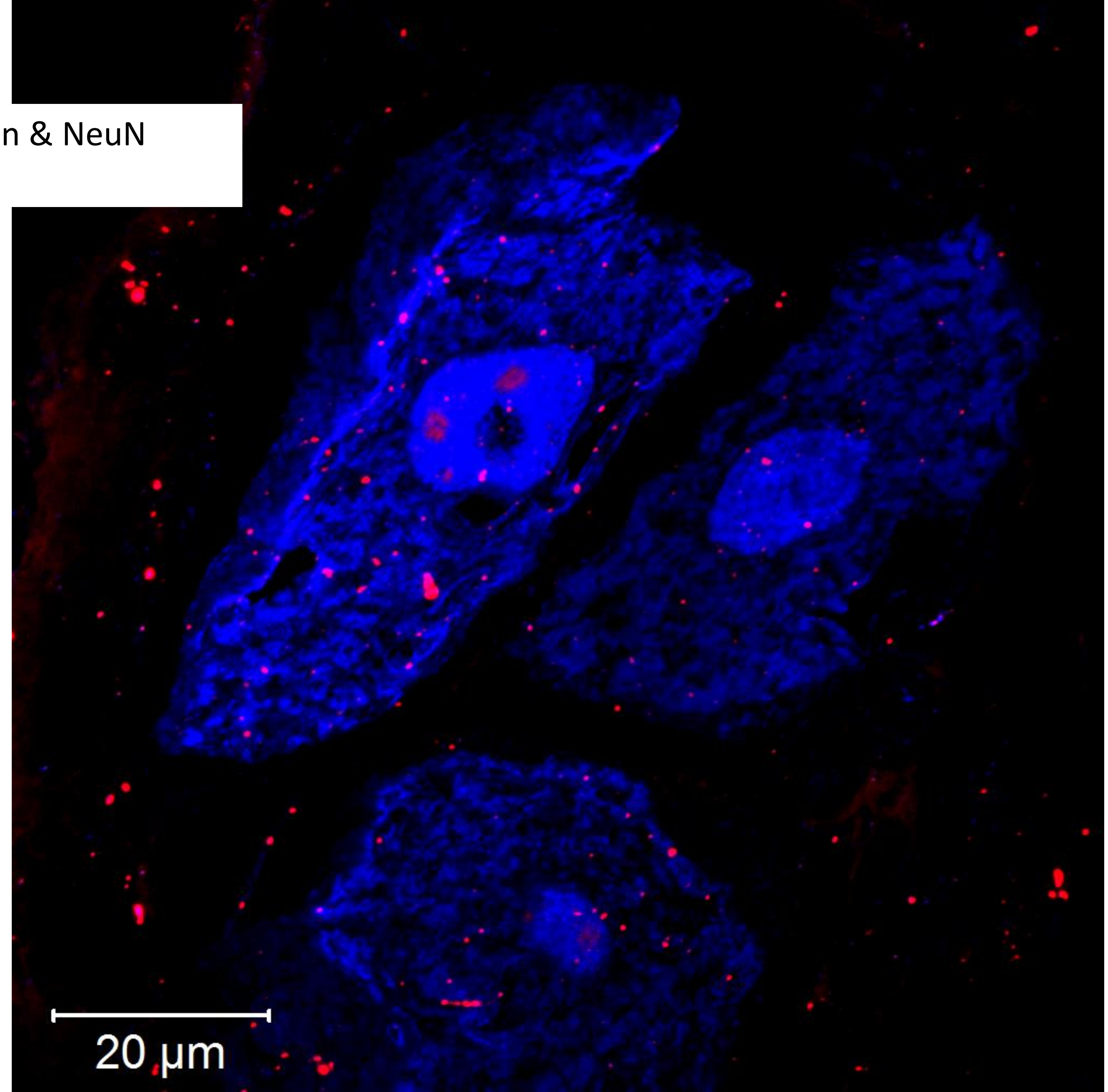

Normal 10-4, GlyRa3
2016-8-24

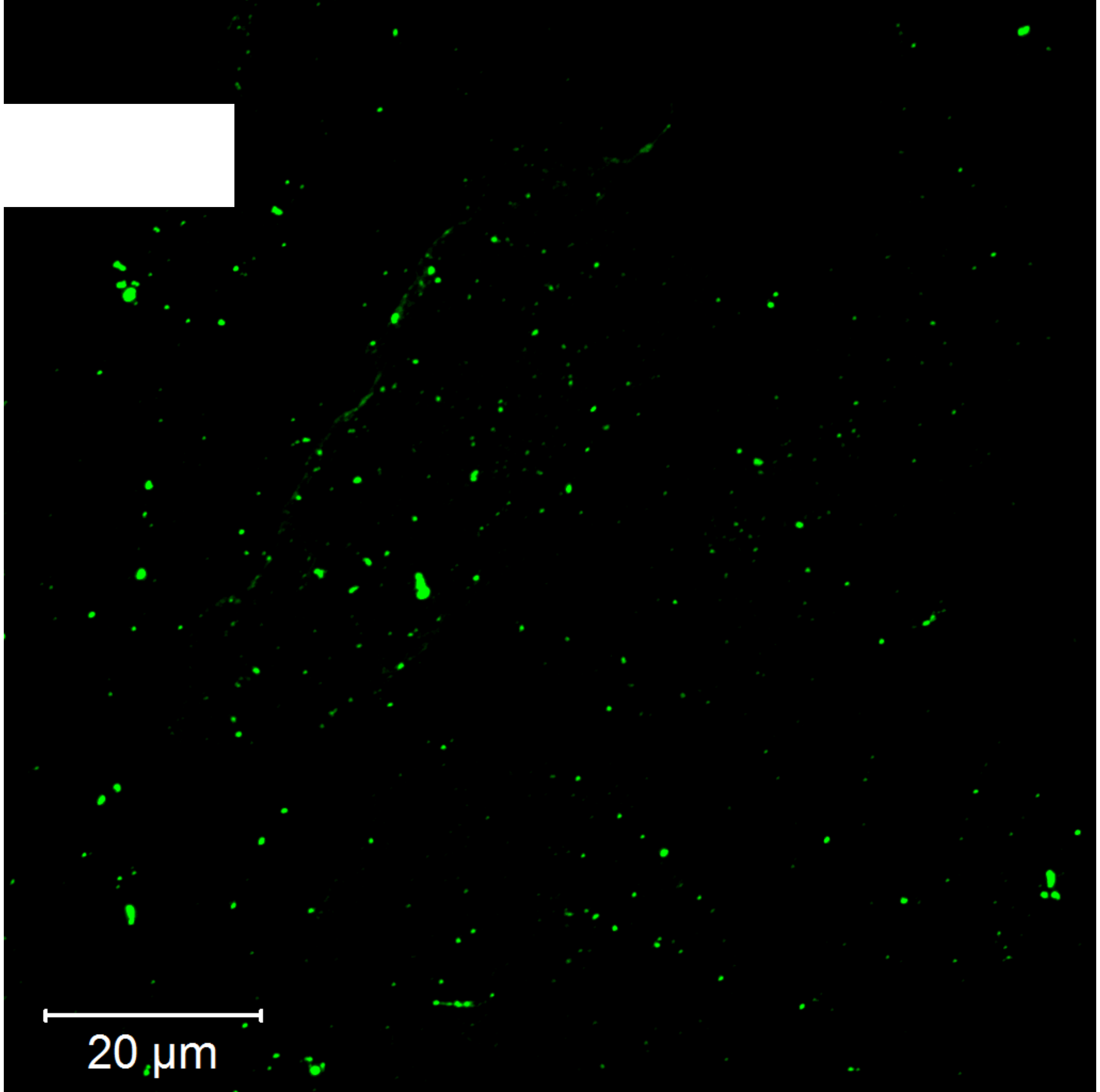

Normal 10-4, GlyRa3 & Gephyrin
2016-8-24

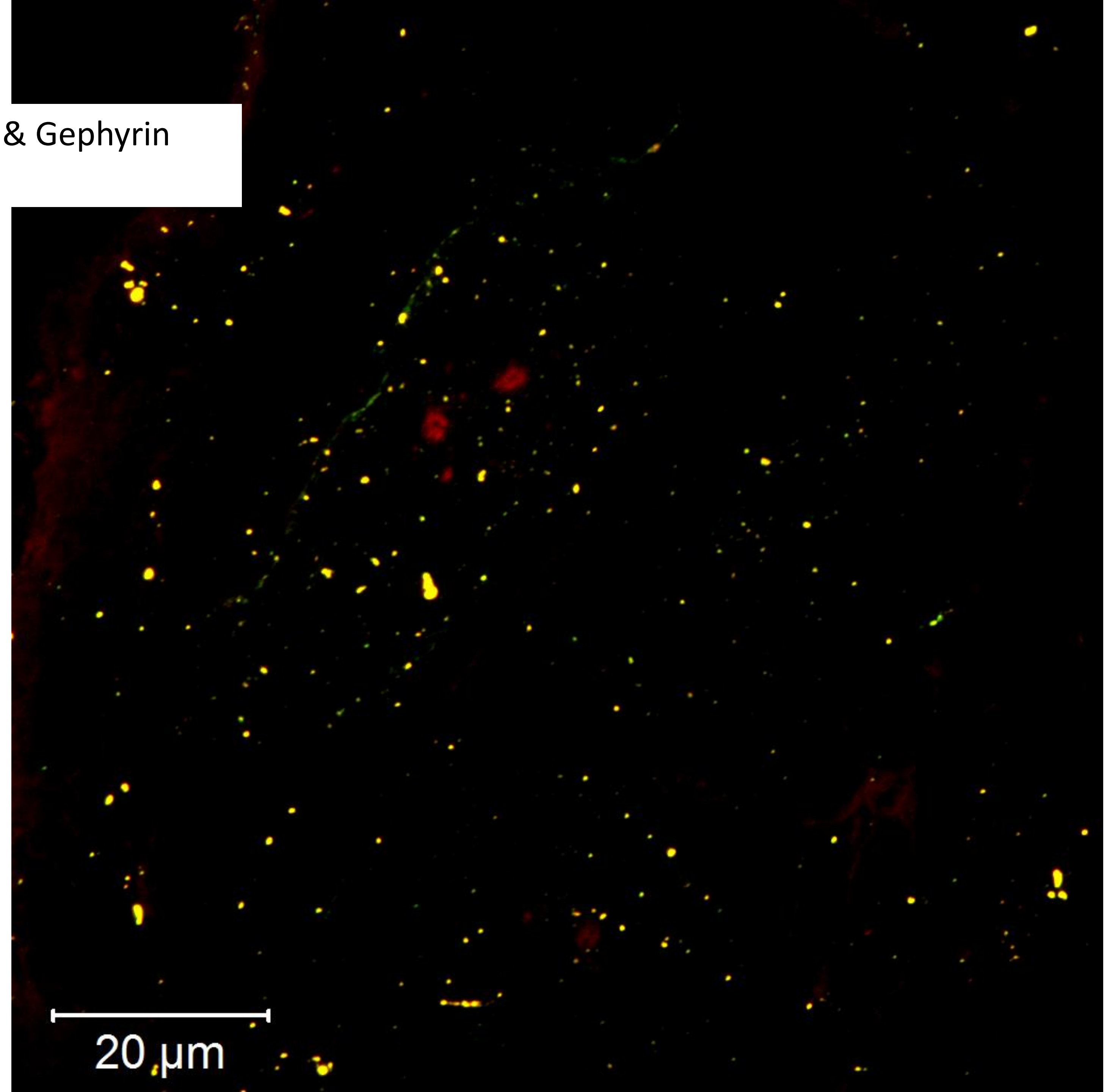

Normal 10-4, GlyRa3 & Gephyrin & NeuN
2016-8-24

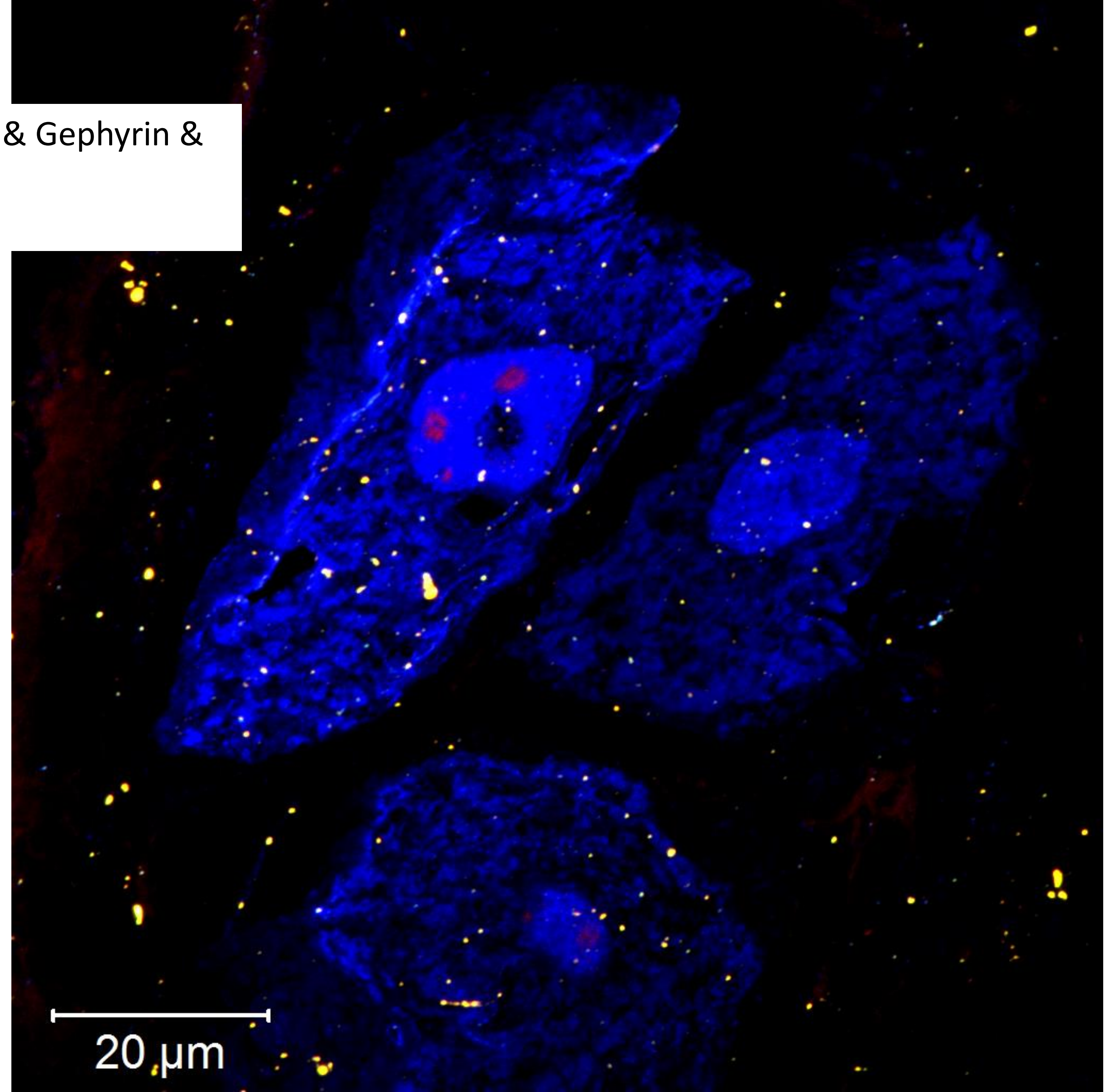

Normal 10-4, GlyRa3 & NeuN
2016-8-24

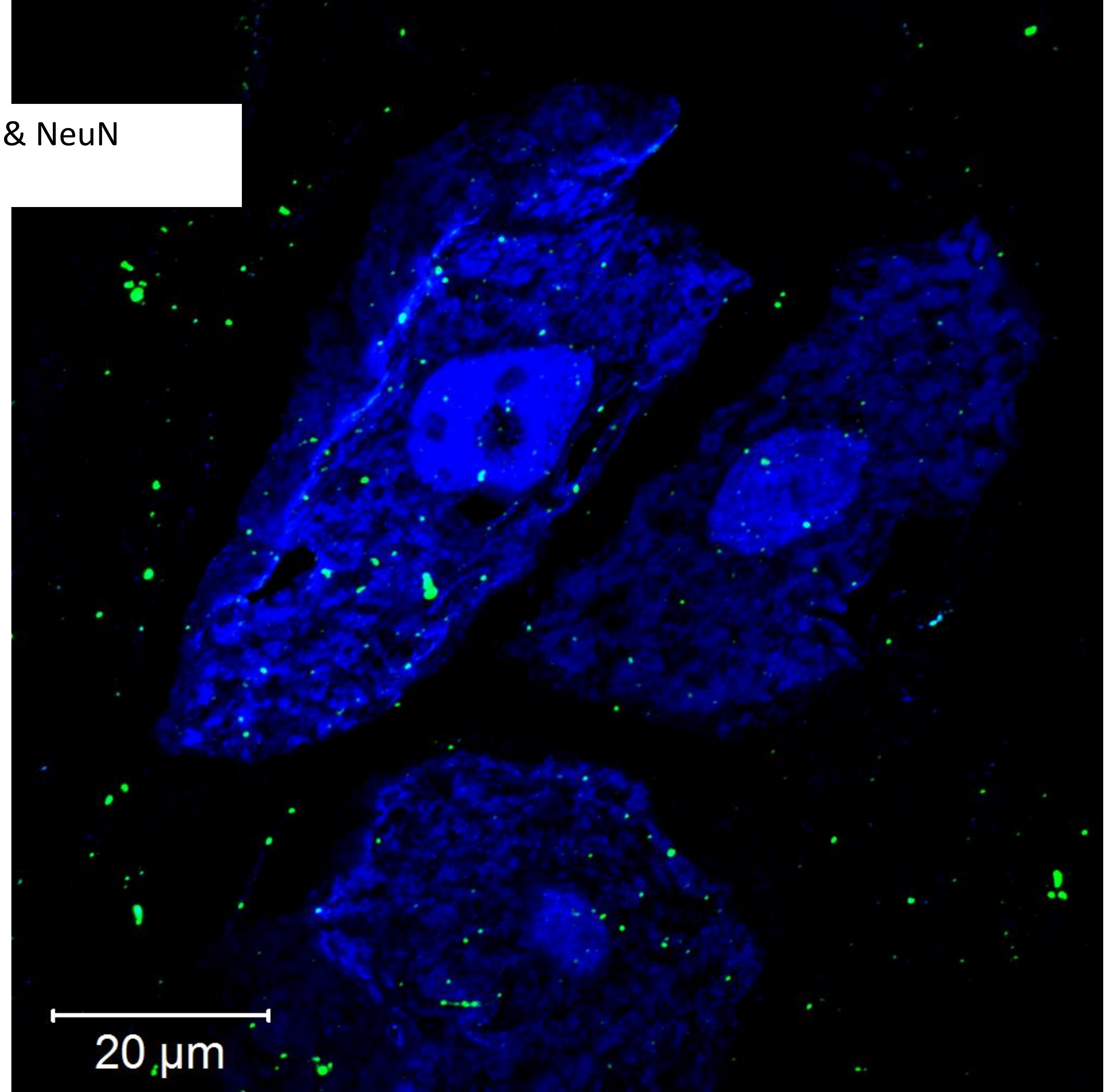

Normal 10-4, NeuN
2016-8-24

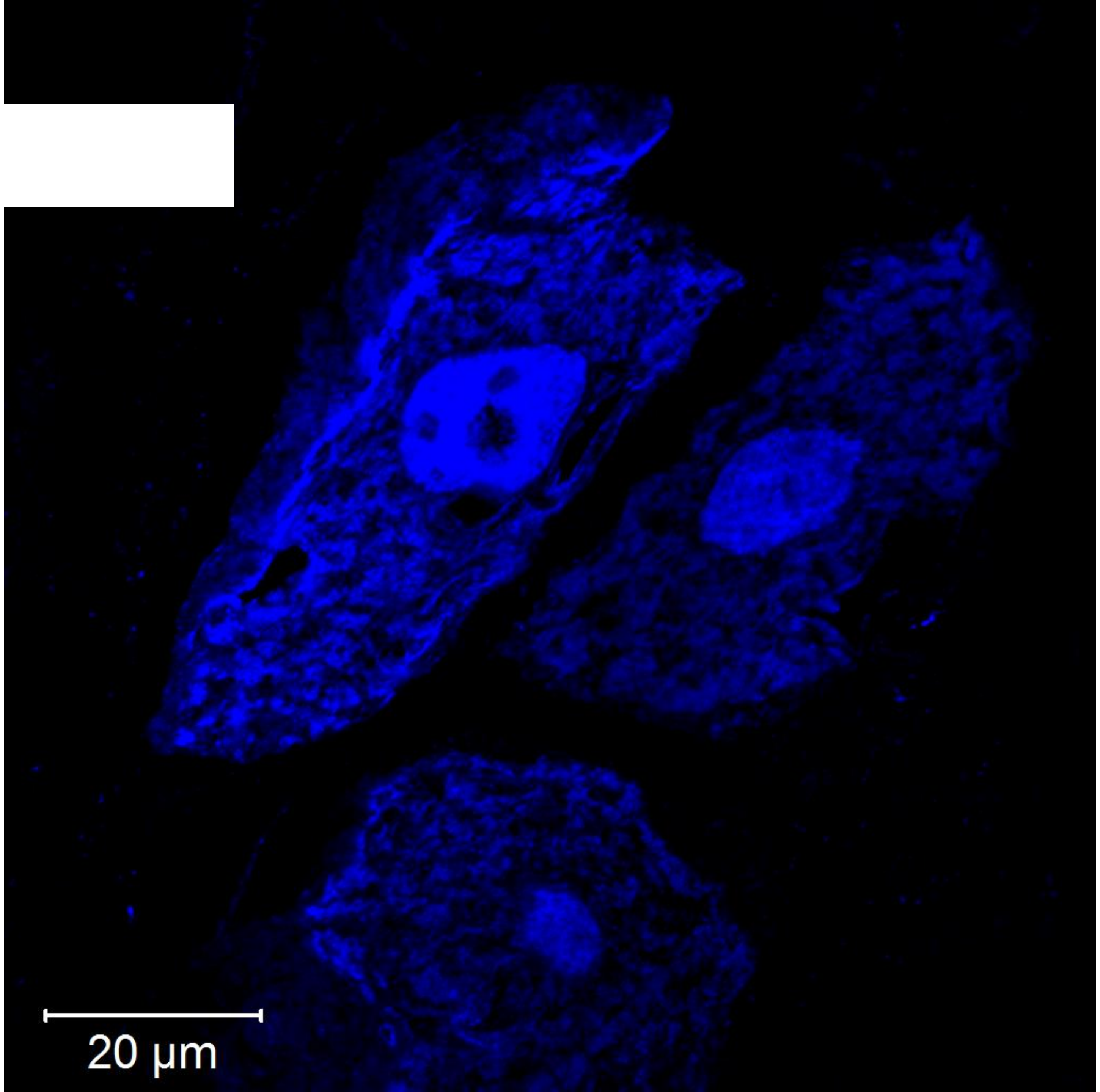

Normal 10-5, Gephyrin
2016-8-24

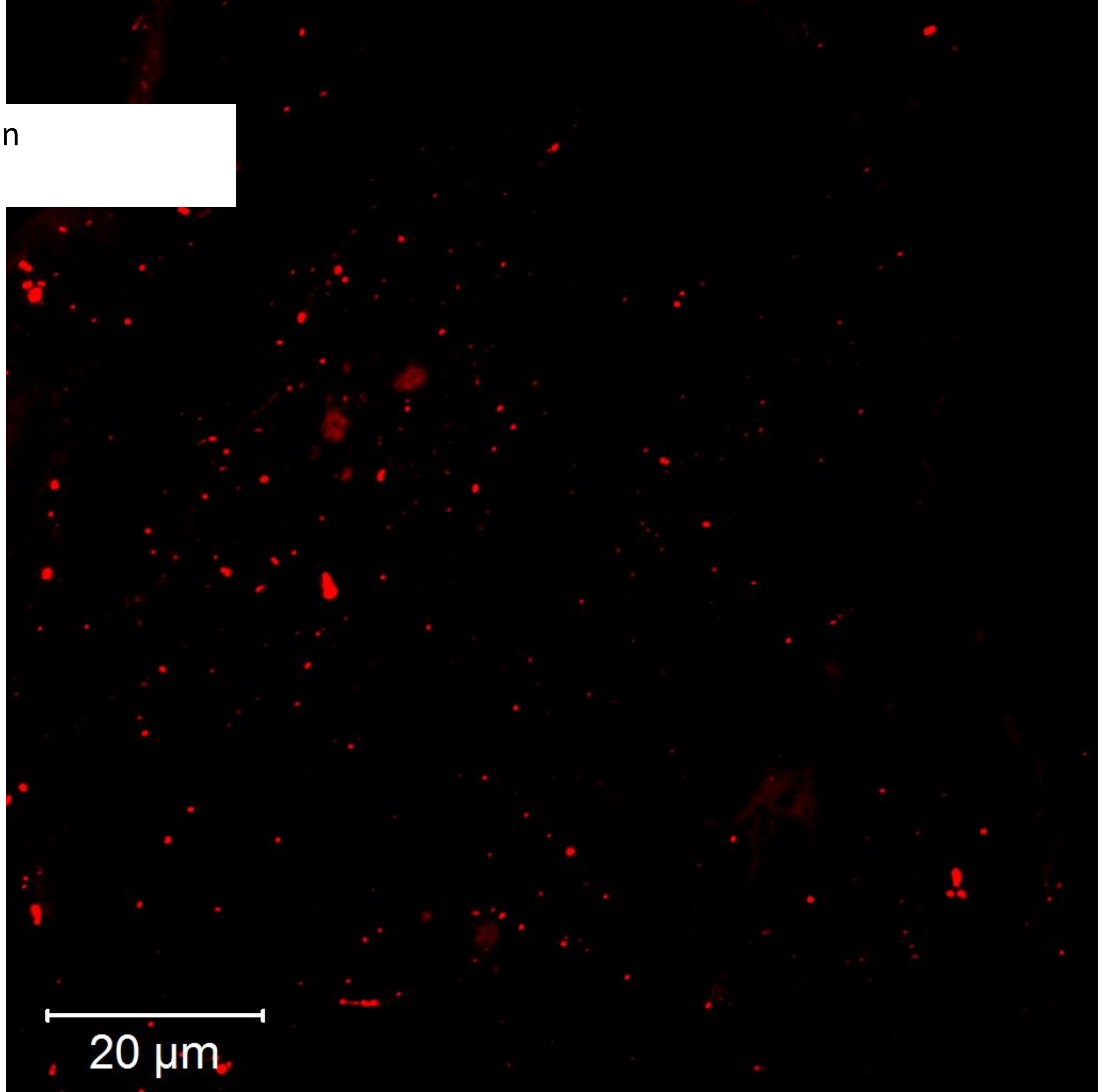

Normal 10-5, Gephyrin & NeuN
2016-8-24

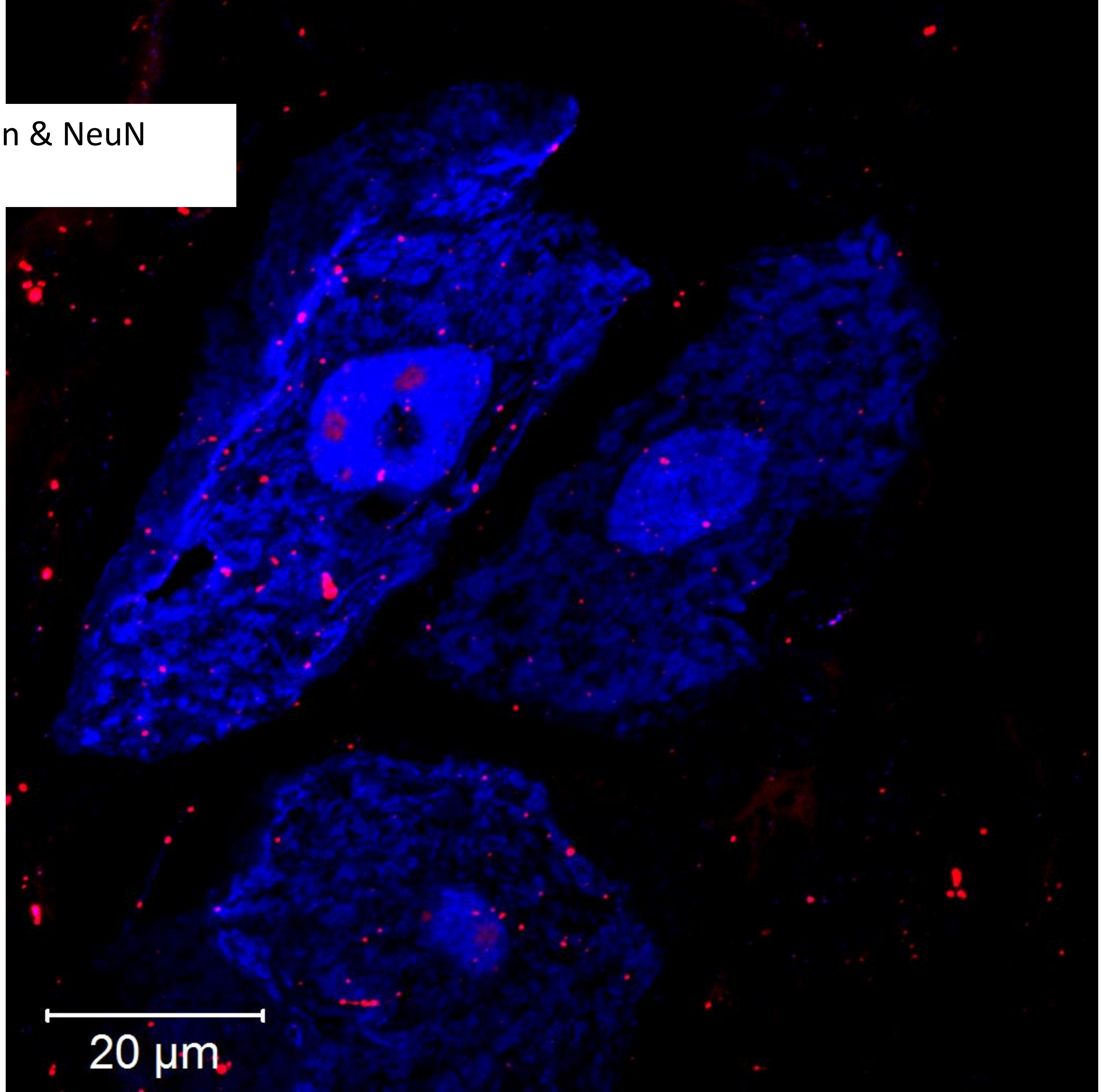

Normal 10-5, GlyRa3
2016-8-24

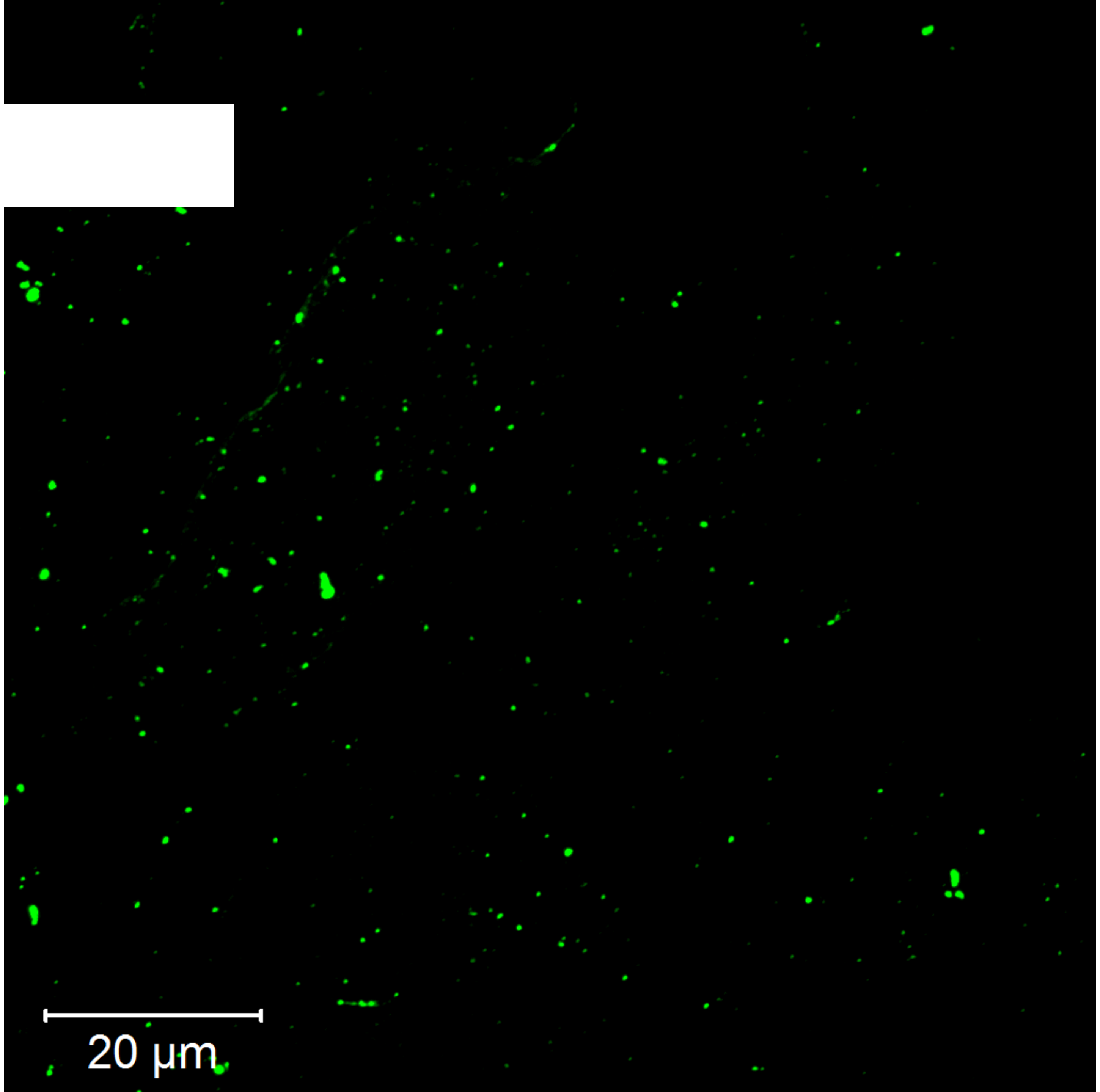

Normal 10-5, GlyRa3 & Gephyrin
2016-8-24

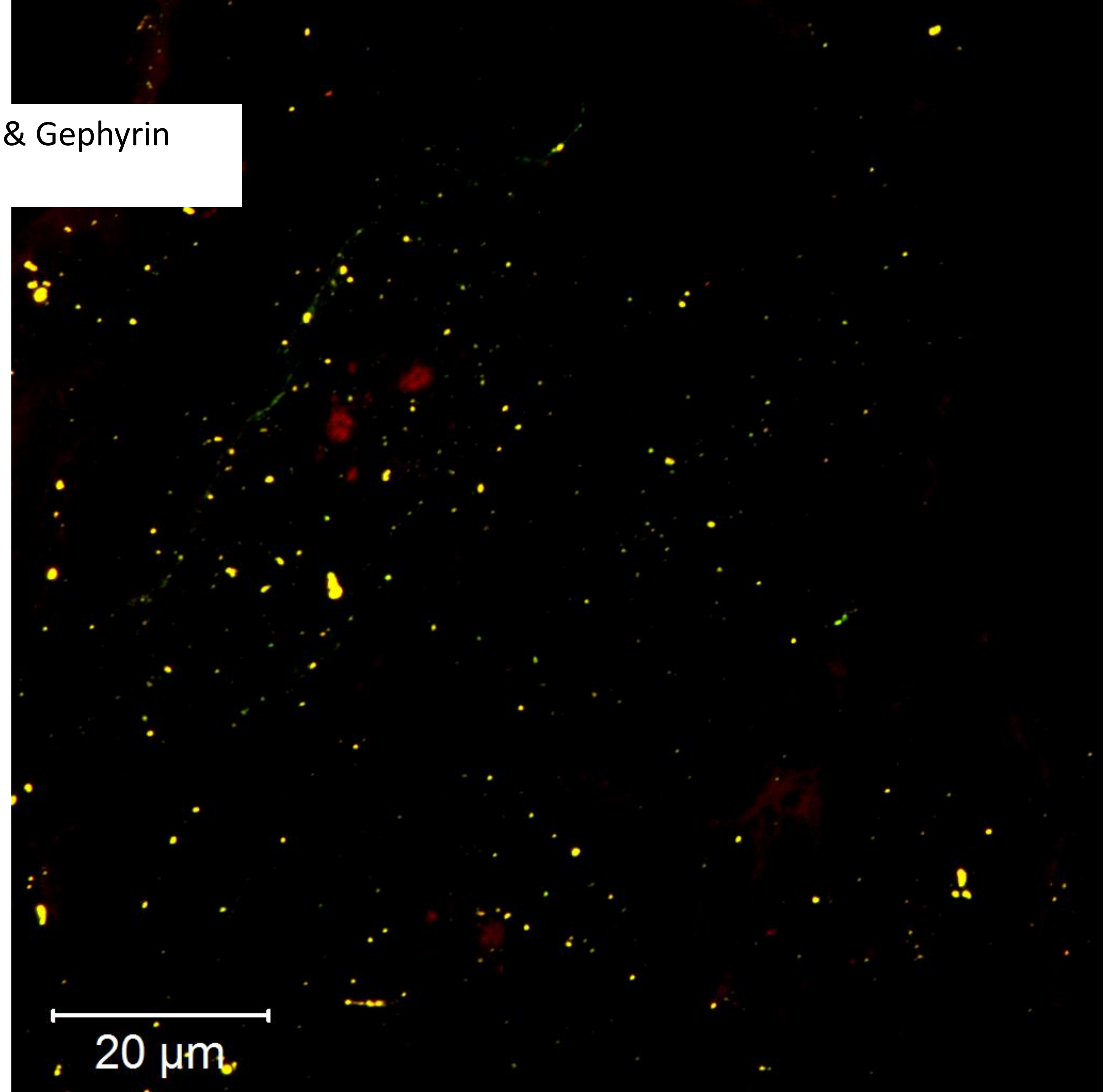

Normal 10-5, GlyRa3 & Gephyrin & NeuN
2016-8-24

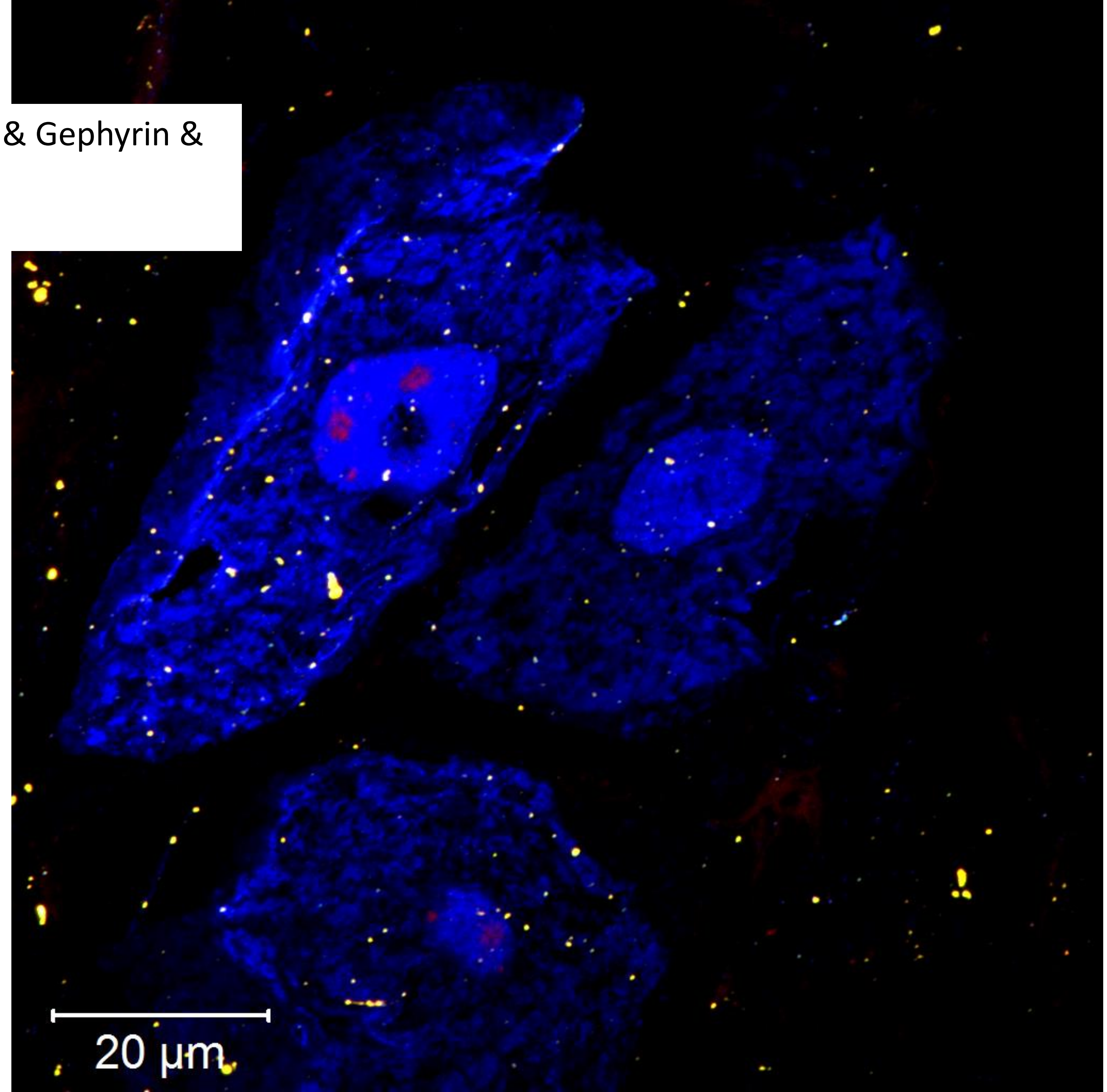

Normal 10-5, GlyRa3 & NeuN
2016-8-24

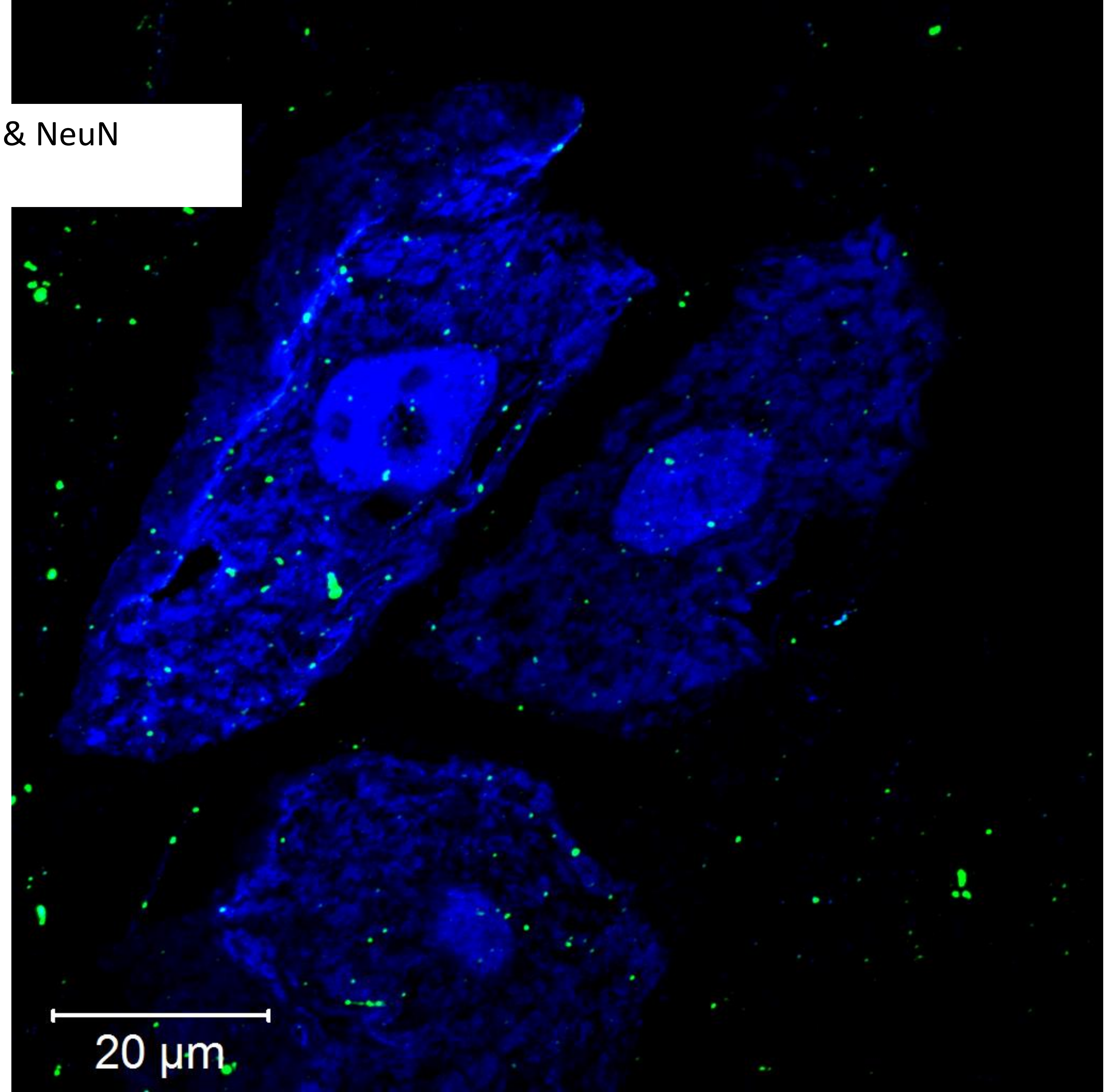

Normal 10-5, NeuN
2016-8-24

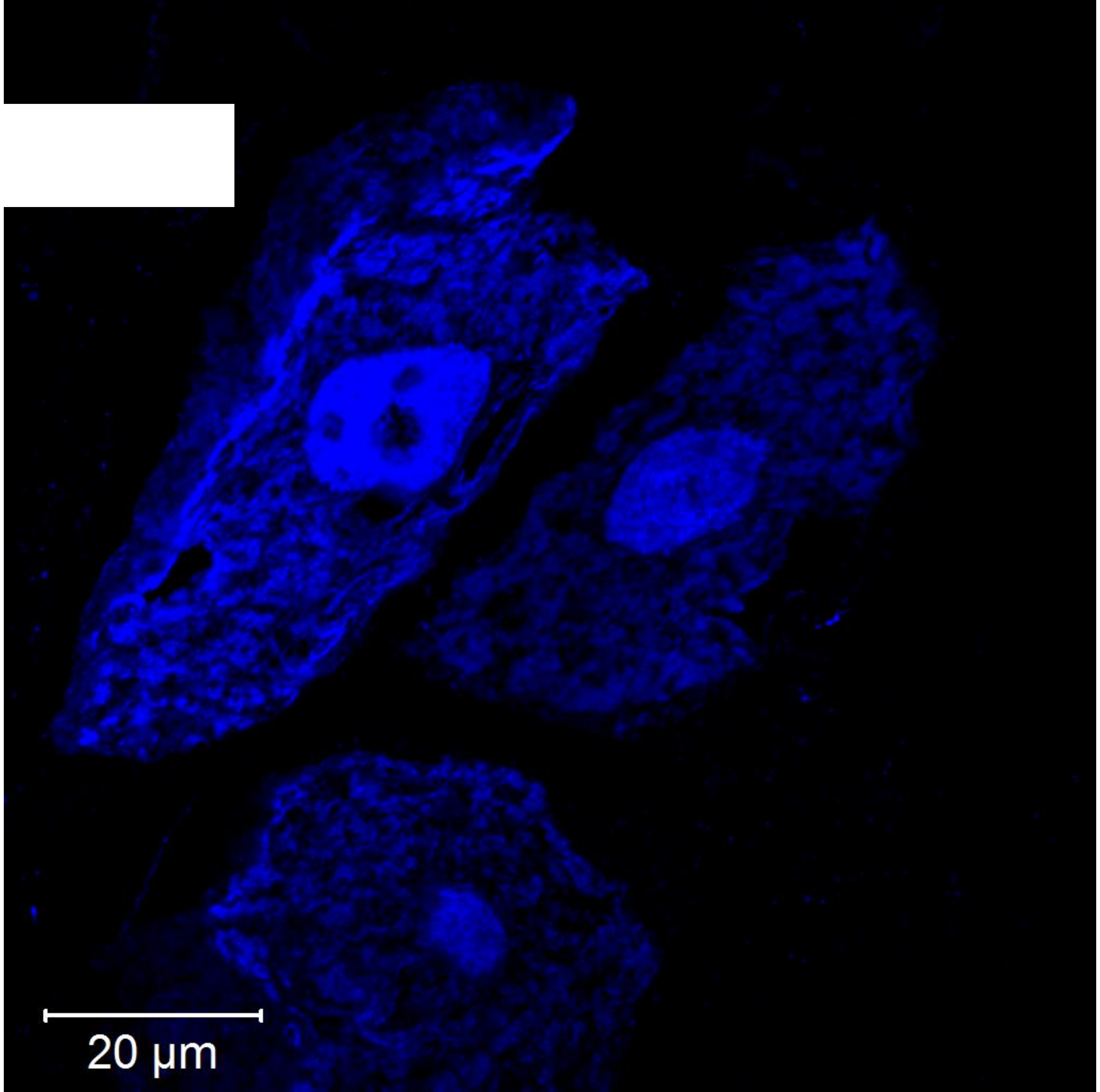

Normal 10-6, Gephyrin
2016-8-24

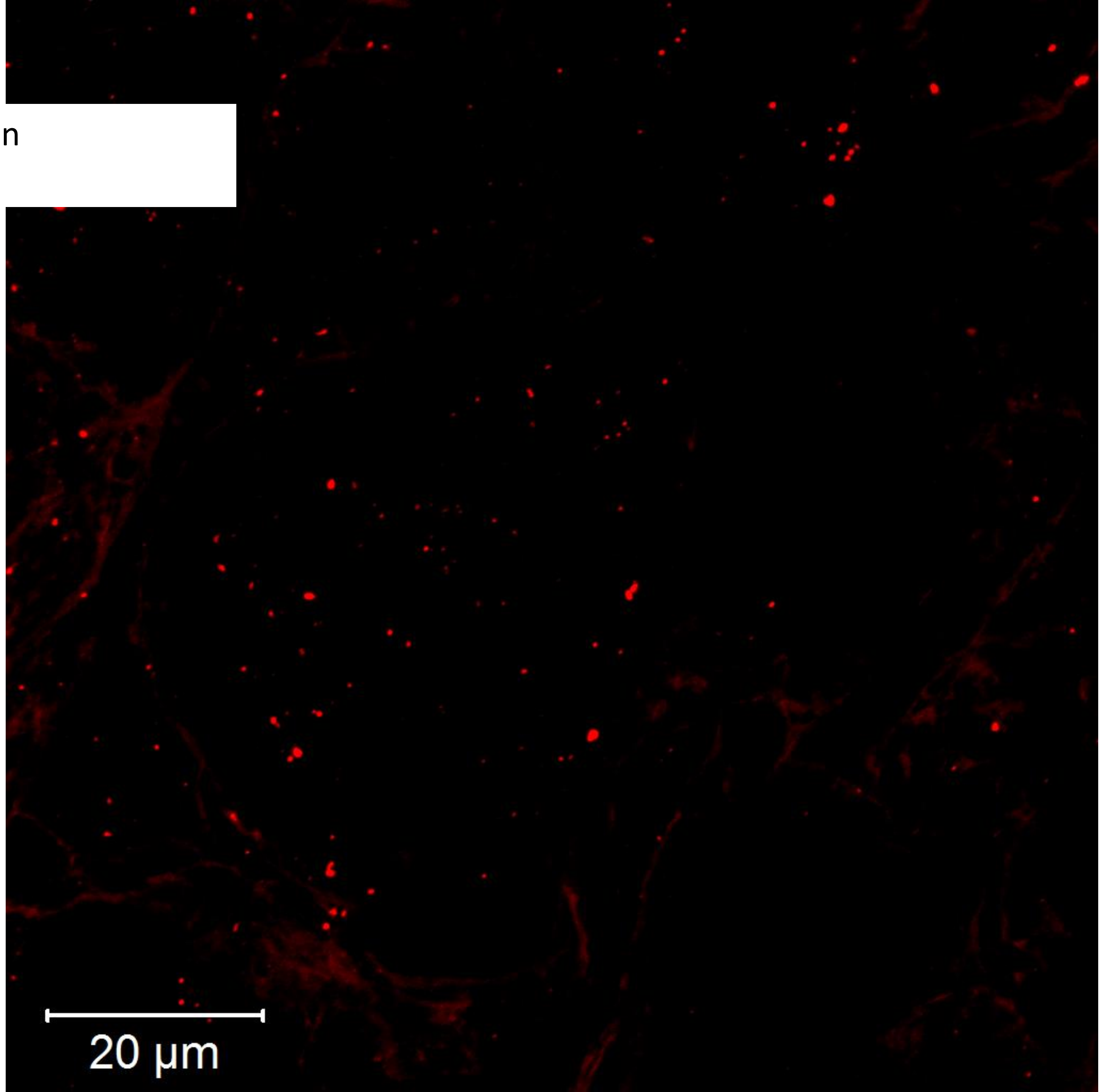

Normal 10-6, Gephyrin & NeuN
2016-8-24

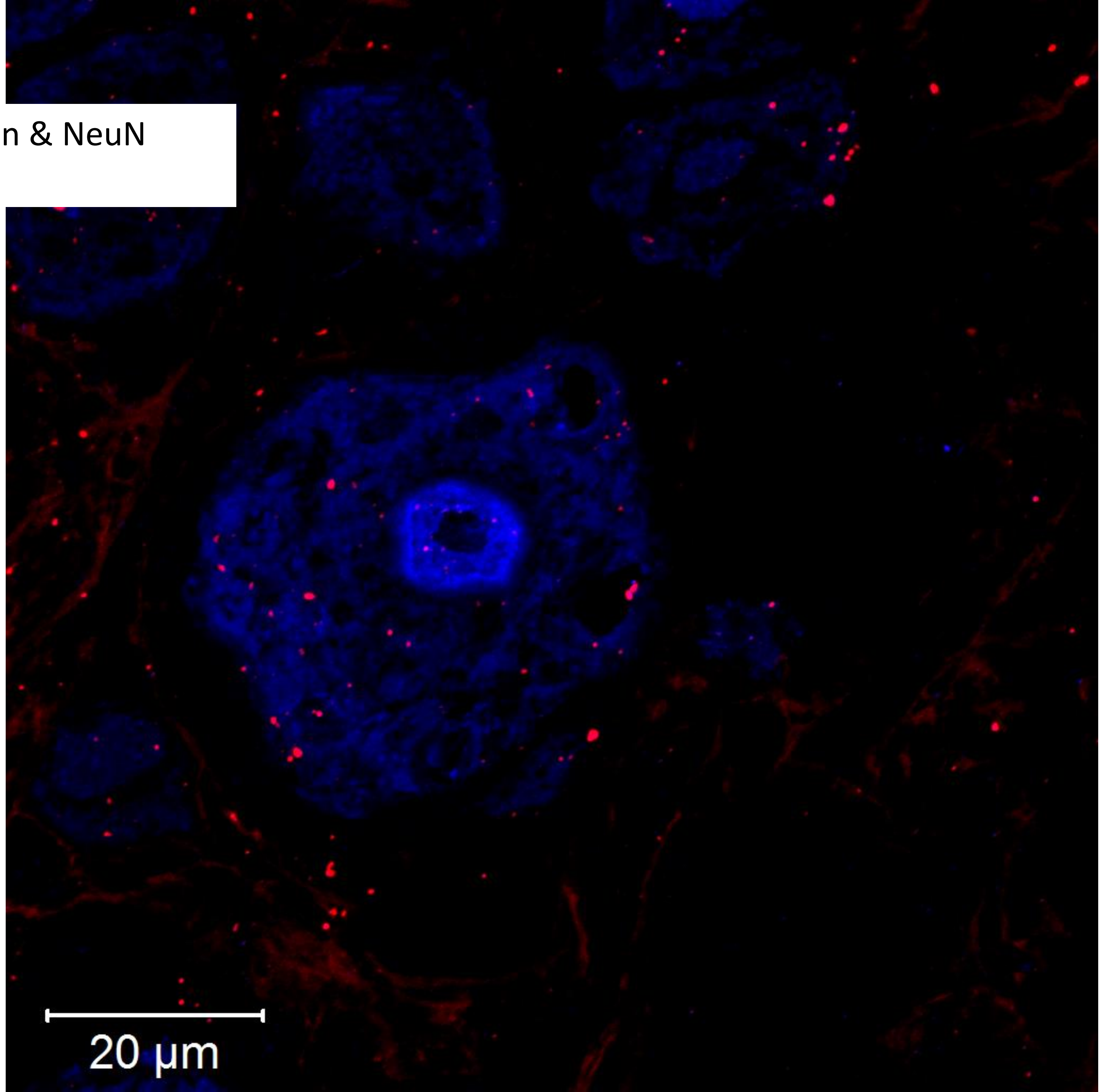

Normal 10-6, GlyRa3
2016-8-24

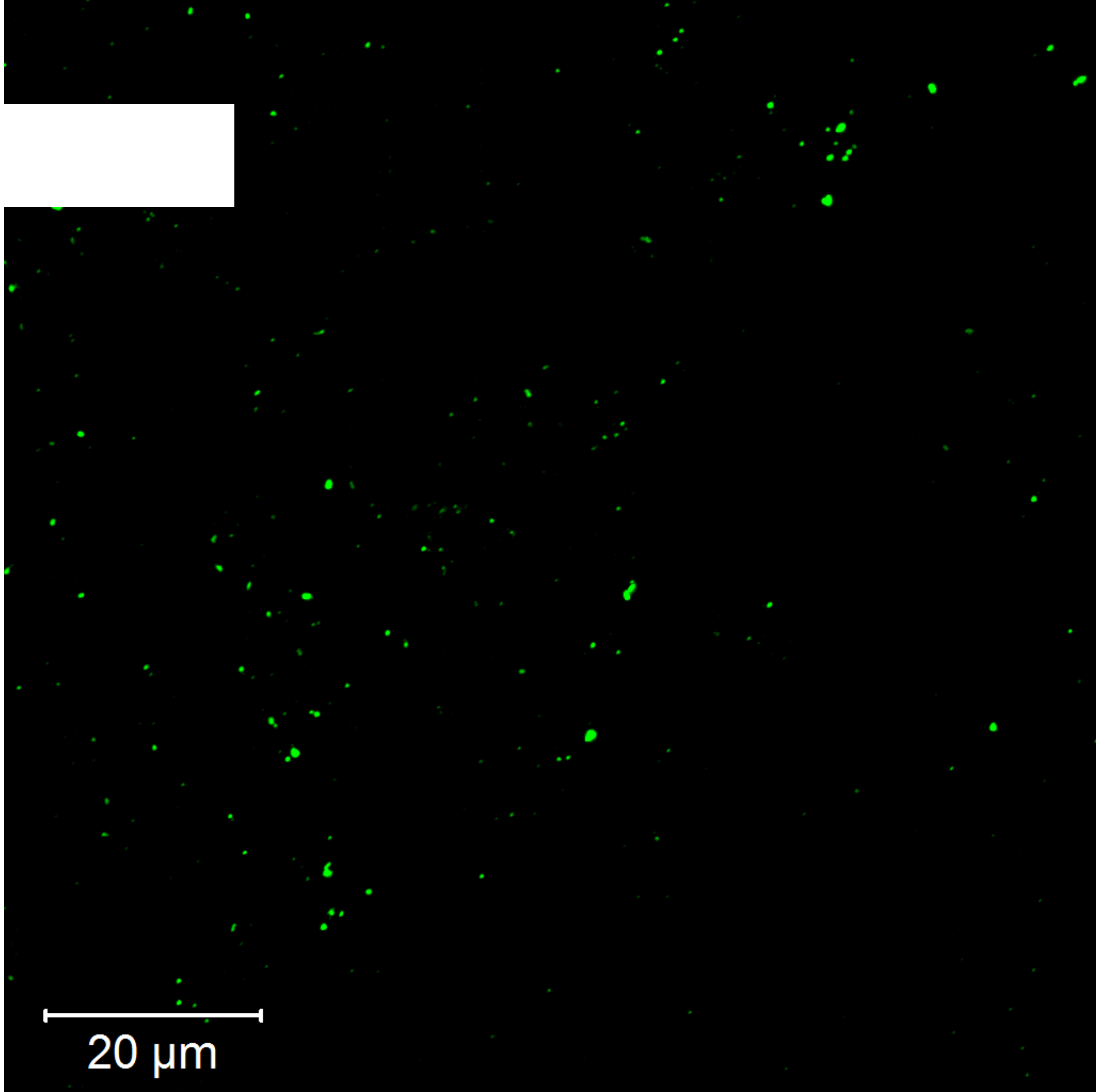

Normal 10-6, GlyRa3 & Gephyrin
2016-8-24

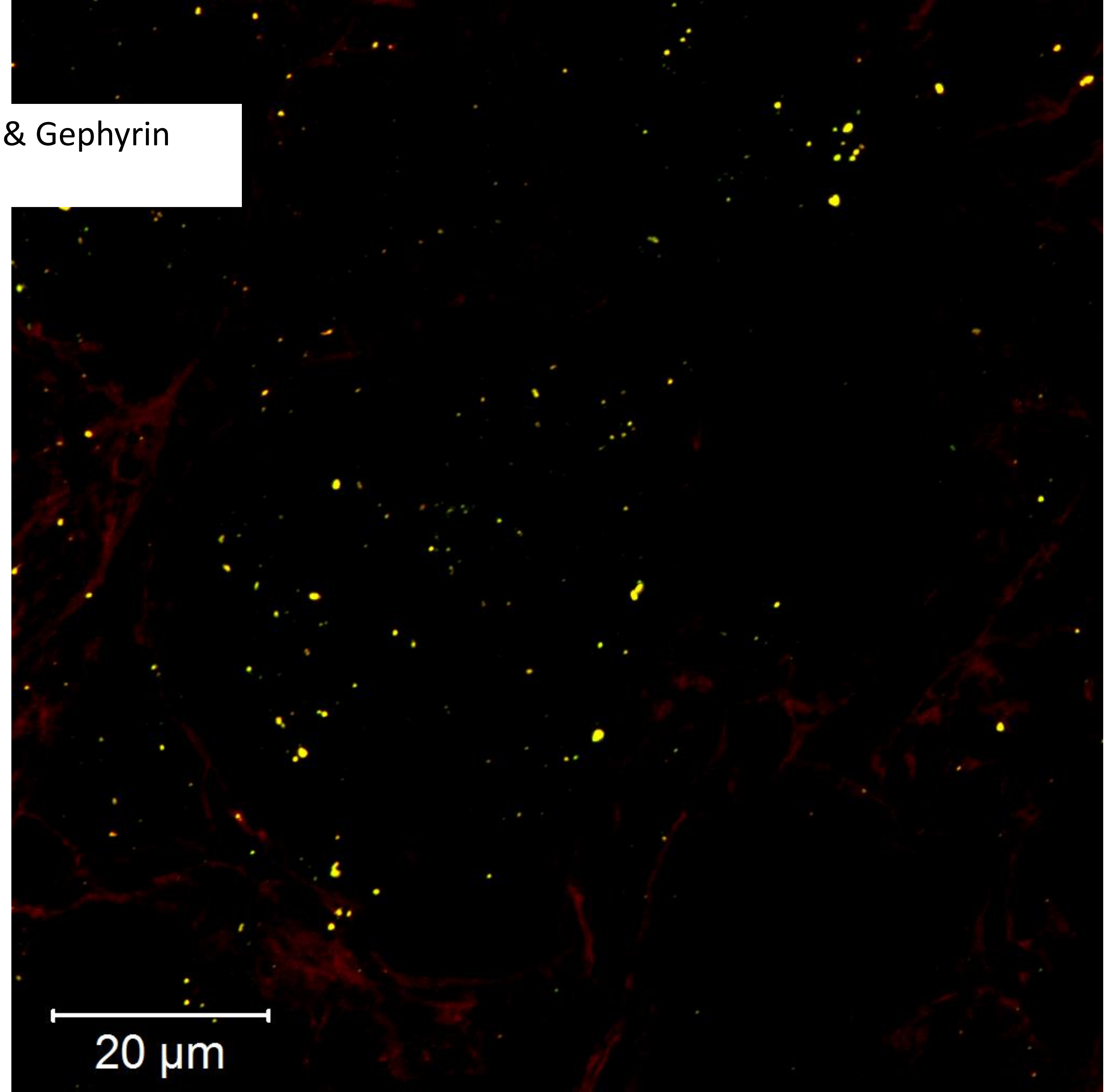

Normal 10-6, GlyRa3 & Gephyrin & NeuN
2016-8-24

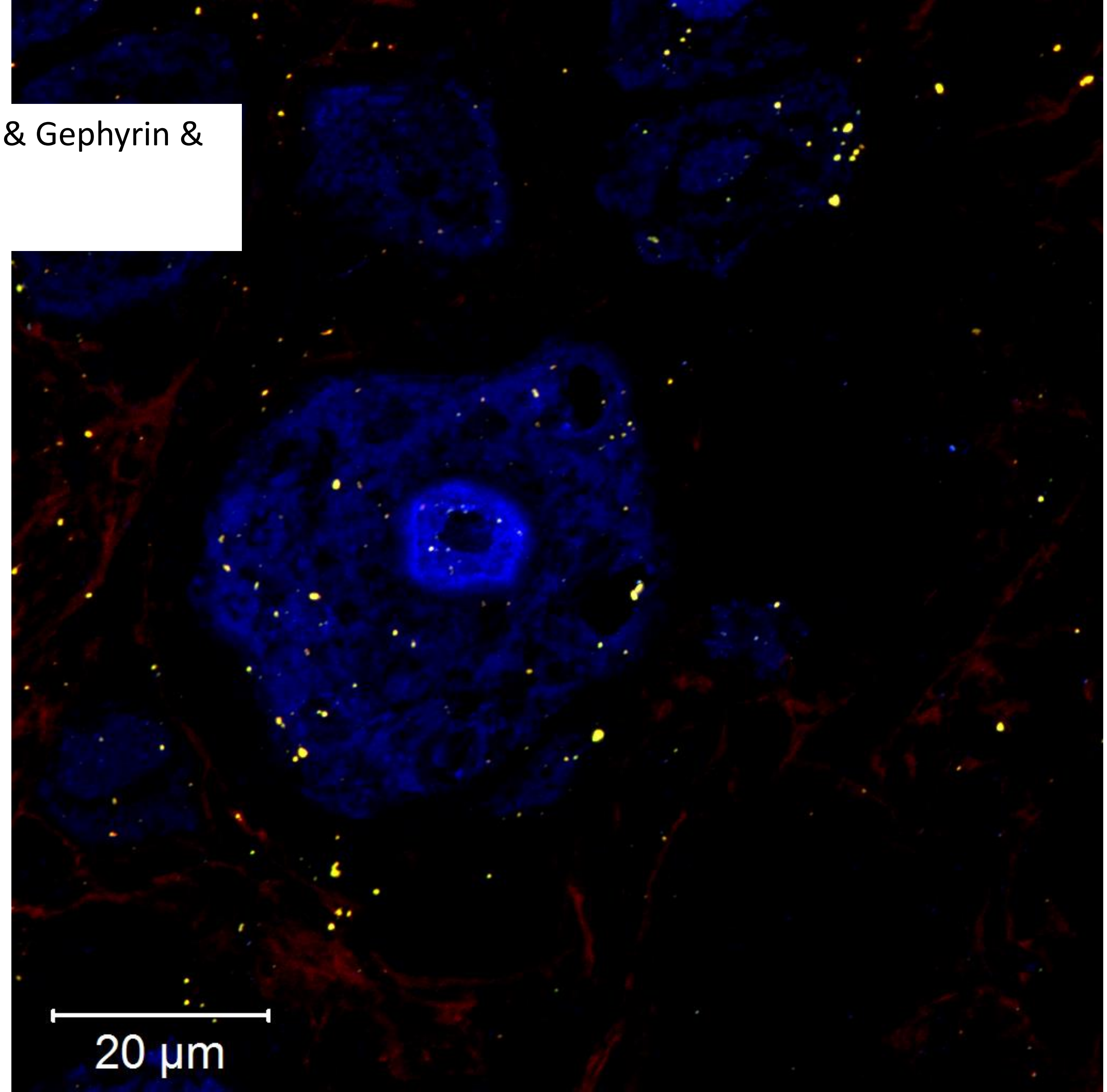

Normal 10-6, GlyRa3 & NeuN
2016-8-24

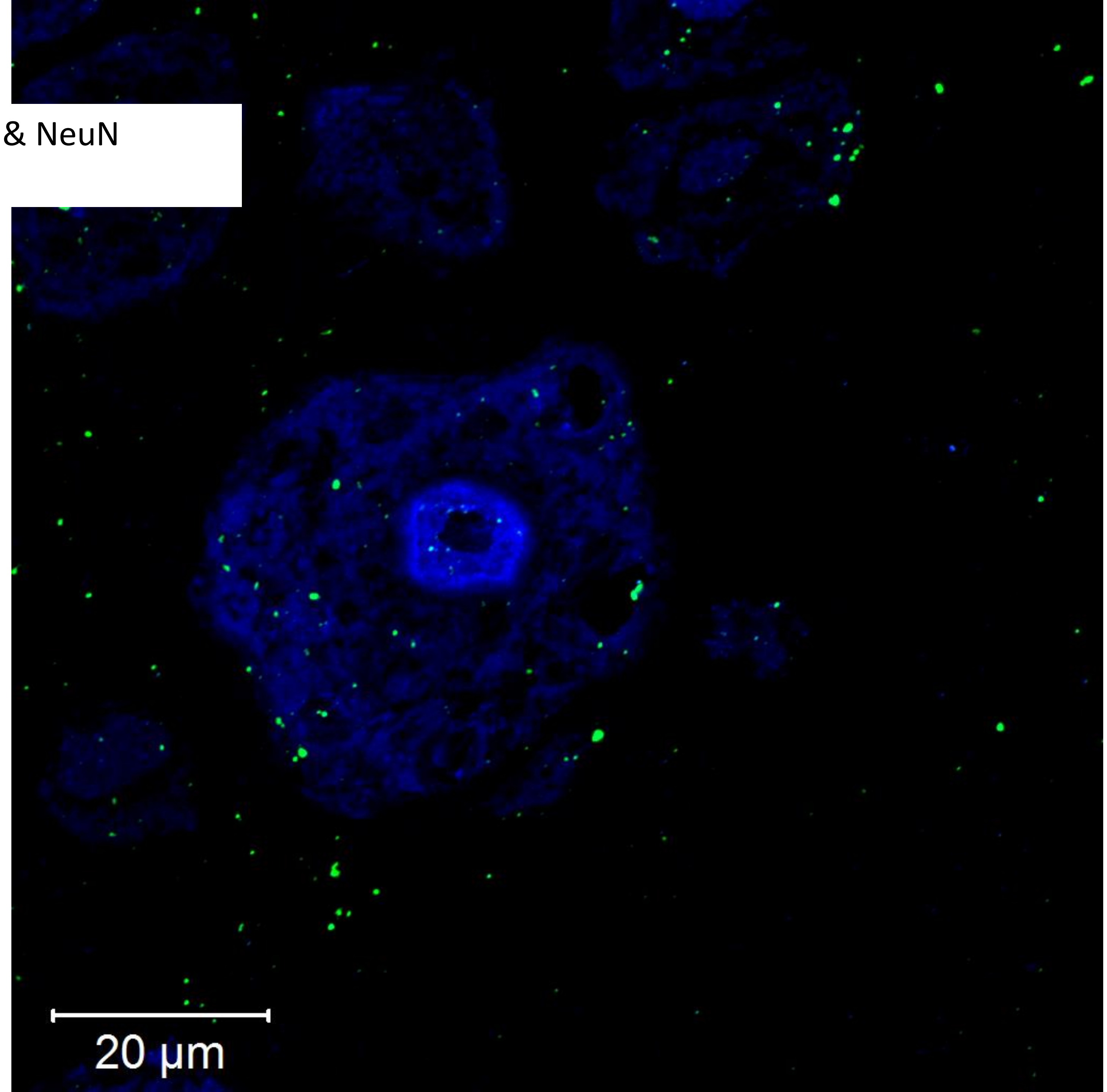

Normal 10-6, NeuN
2016-8-24

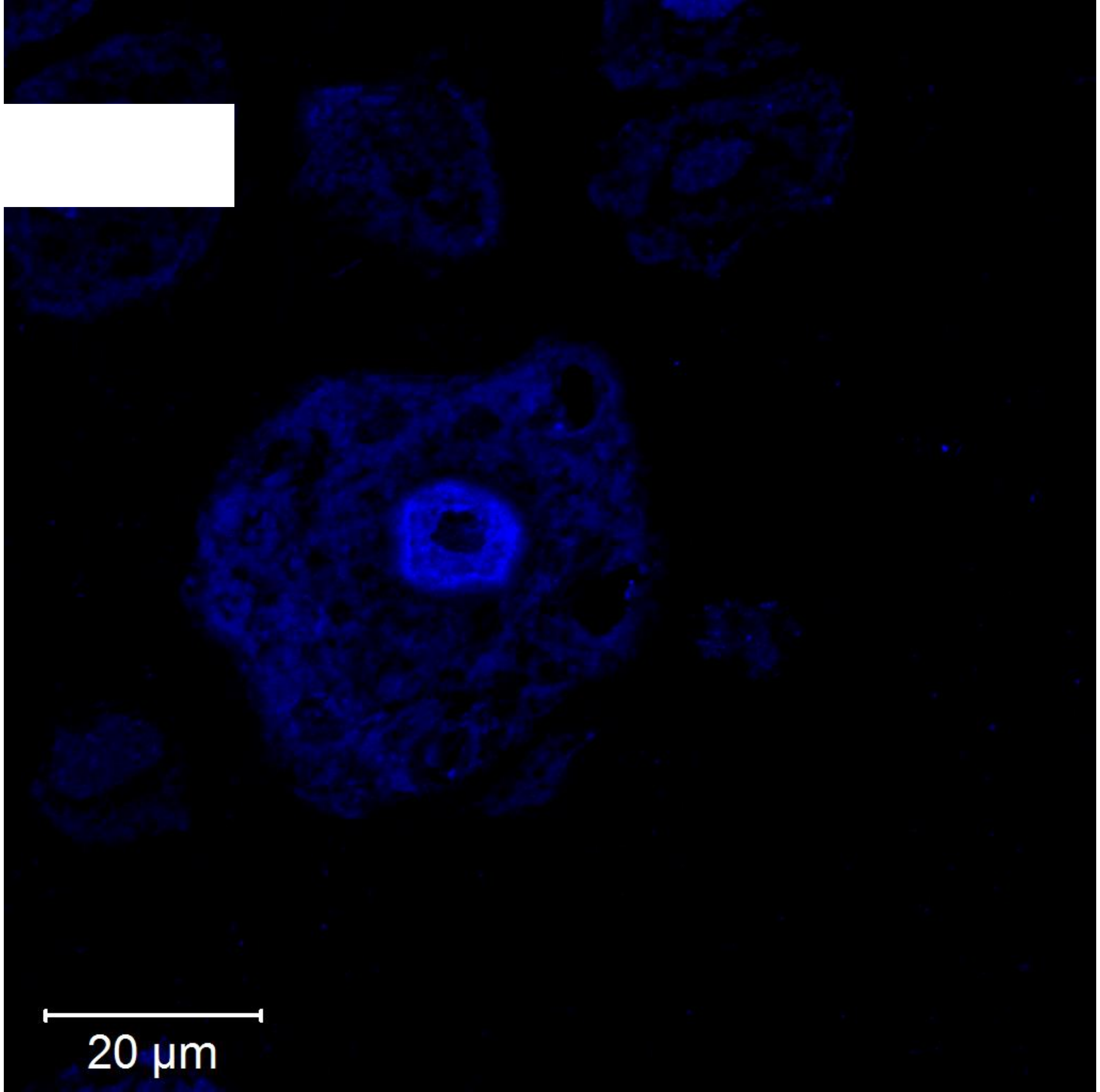

Supplement: Supplementary file 5 — Additional file 5. Triple immunofluorescence staining in the L5 DRG (Control group); Triple immunofluorescence staining showing GlyRα3, Gephyrin and NeuN co-localization in the L5 DRG. [file 12868_2018_470_MOESM5_ESM.pdf]
