# Supplementary material for: Glycine receptors expression in rat spinal cord and dorsal root ganglion in prostaglandin E2 intrathecal injection models
Source: BMC Neurosci. 2018 Nov 9;19:72. doi: 10.1186/s12868-018-0470-8 (PMC6230273; doi:10.1186/s12868-018-0470-8)
Supplement: Supplementary file 6 — Additional file 6. Triple immunofluorescence staining in the L5 DRG; Triple immunofluorescence staining showing GlyRα3, Gephyrin and NeuN co-localization in the L5 DRG. [file 12868_2018_470_MOESM6_ESM.pdf]

整理

1°Ab(host/廠牌/貨號)	dilute	2°Ab (host/廠牌/貨號)	dilute
GlyRα3 (goat/ santa cruz/ SC-17282)	1:50	Alexa Fluor® 488 (Dondey anti-goat / Jackson ImmunoResearch/705-545-147)	1:200
Gephyrin (mouse/ synaptic systems/ 147 021)	1:200	Cy5 (Donkey anti-mouse/ Jackson ImmunoResearch/ 715-175-151)	1:200
Neu N (Chicken/ millpore/ ABN91)	1:200	DyLight™ 405 (Goat anti-chicken/ Jackson ImmunoResearch/ 103-475-155)	1:200

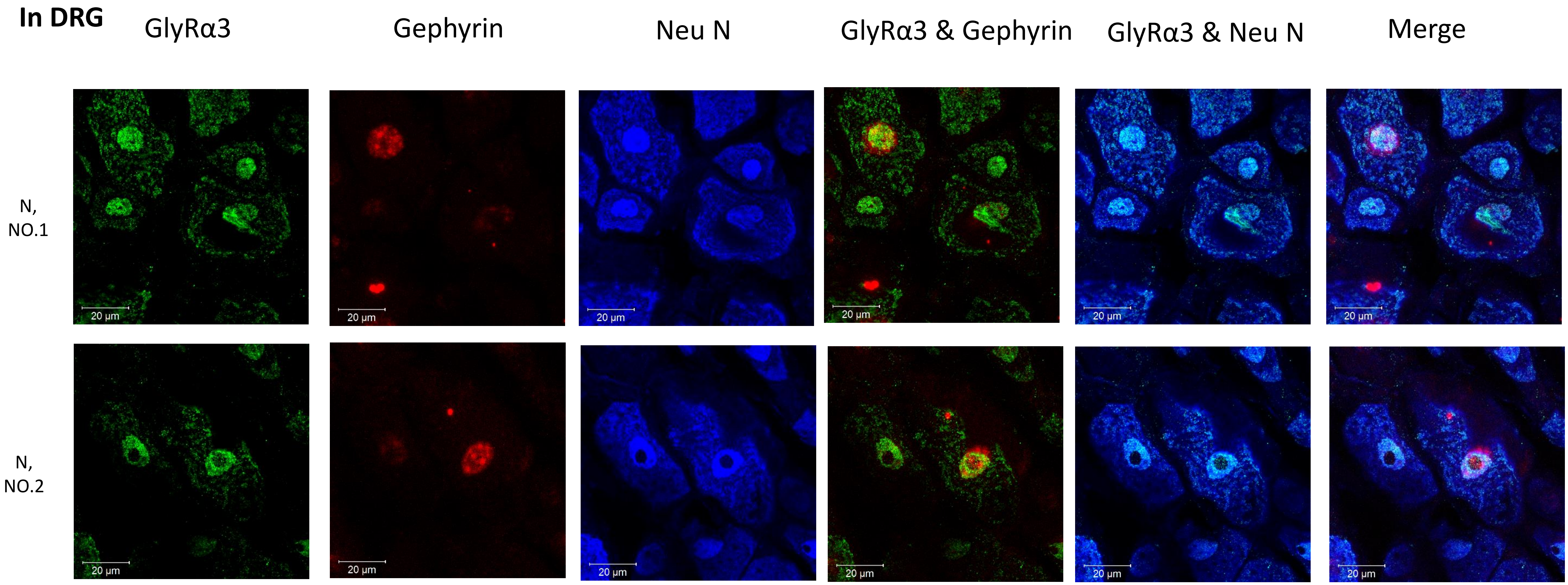
In DRG
GlyRα3
Gephyrin
Neu N
GlyRα3 & Gephyrin
GlyRα3 & Neu N
Merge
N,
NO.1
N,
NO.2
20 µm

1抗(host/廠牌/貨號)	dilute	2抗(host/廠牌/貨號)	dilute
GlyRα3 (goat/ santa cruz/ SC-17282)	1:50	Alexa Fluor® 488 (Dondey anti-goat / Jackson ImmunoResearch/705-545-147)	1:200
Gephyrin (mouse/ synaptic systems/ 147 021)	1:200	Cy™3 (Goat anti-mouse/ Jackson ImmunoResearch/ 115-165-003)	1:200
Neu N (Chicken/ millpore/ ABN91)	1:200	DyLight™ 405 (Goat anti-chicken/ Jackson ImmunoResearch/ 103-475-155)	1:200

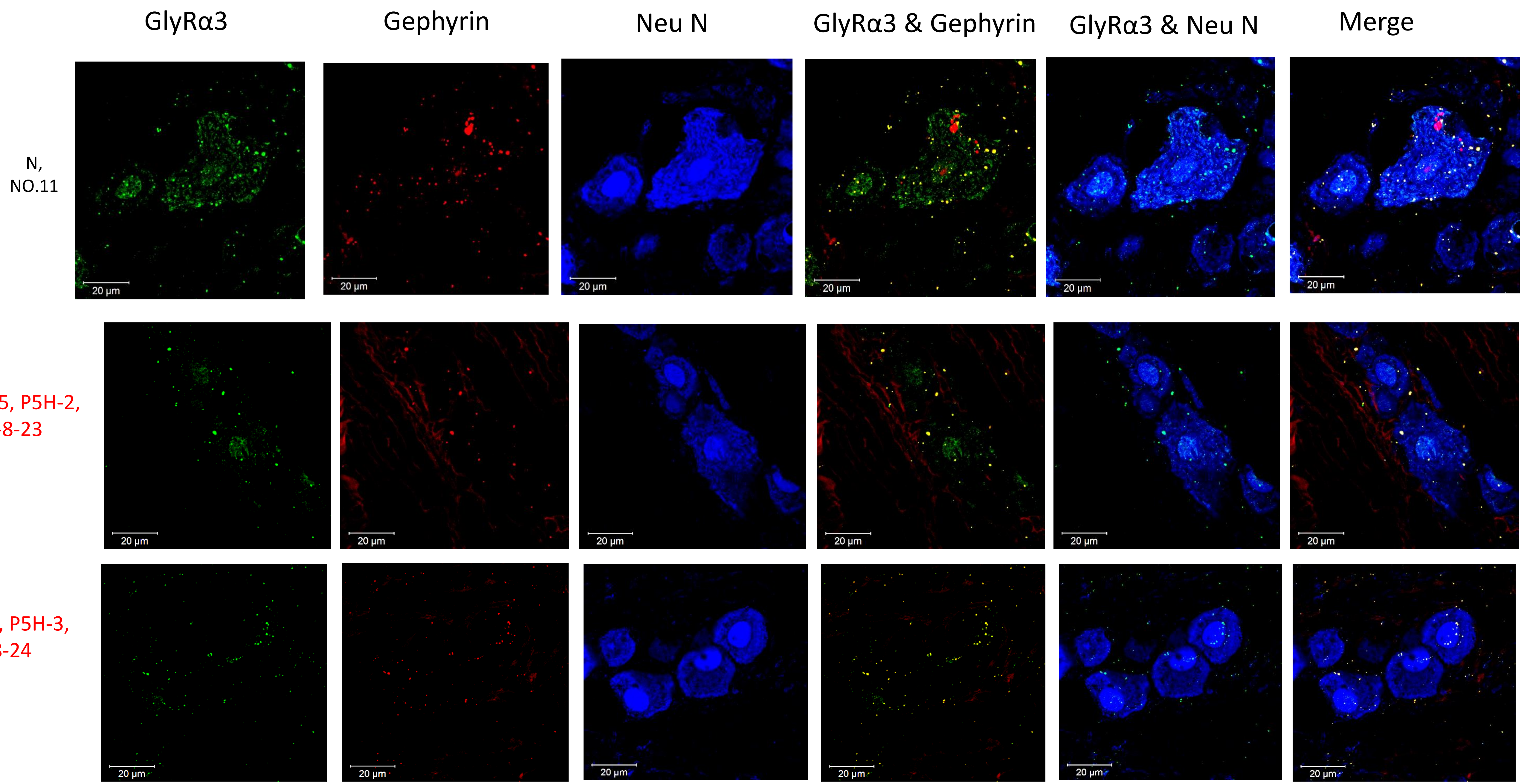
GlyRα3
Gephyrin
Neu N
GlyRα3 & Gephyrin
GlyRα3 & Neu N
Merge
N,
NO.11
5, P5H-2,
-8-23
, P5H-3,
3-24
20 μm

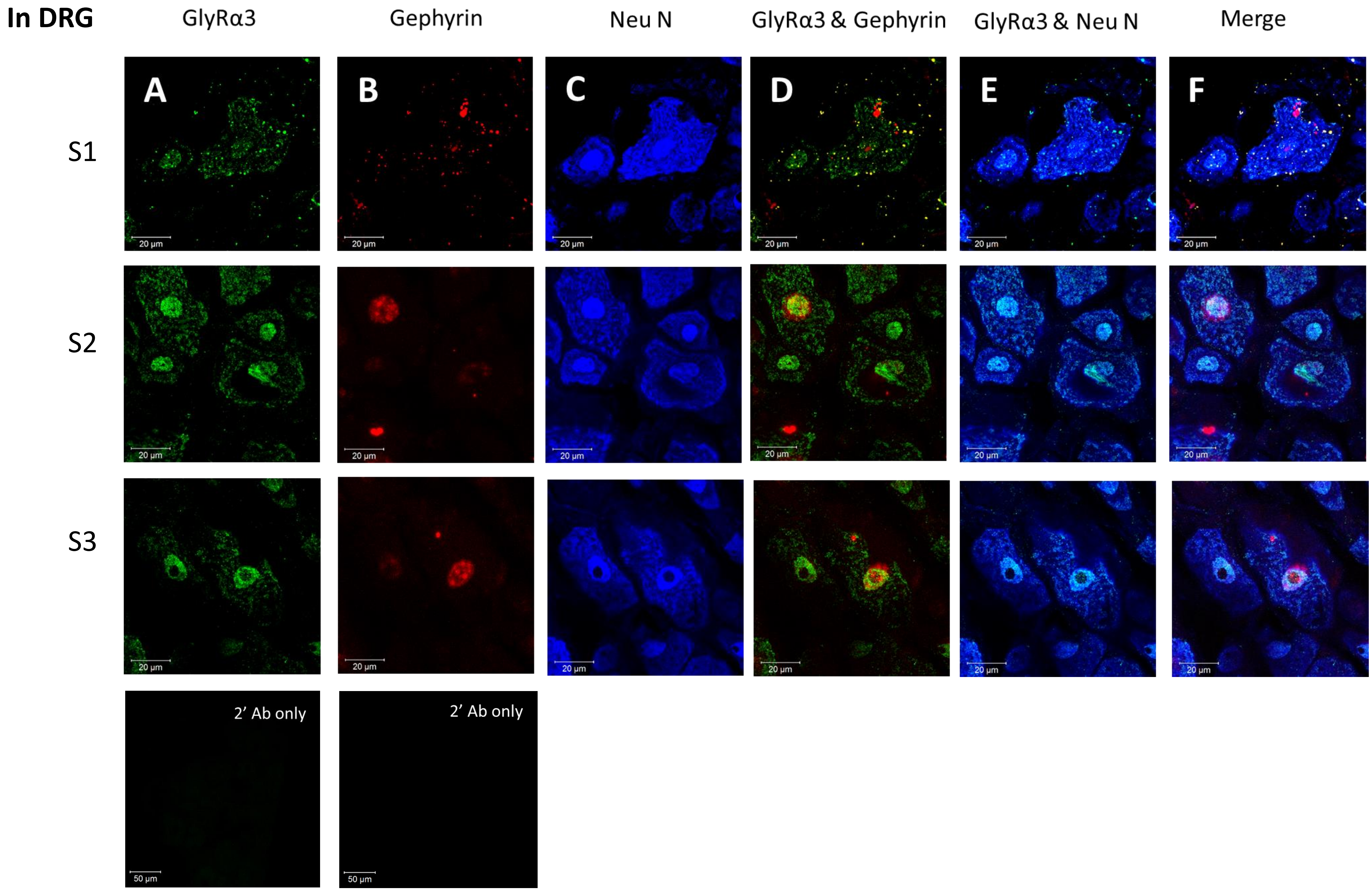
In DRG
GlyRα3
Gephyrin
Neu N
GlyRα3 & Gephyrin
GlyRα3 & Neu N
Merge
A
B
C
D
E
F
S1
S2
S3
20 μm
2' Ab only
50 μm

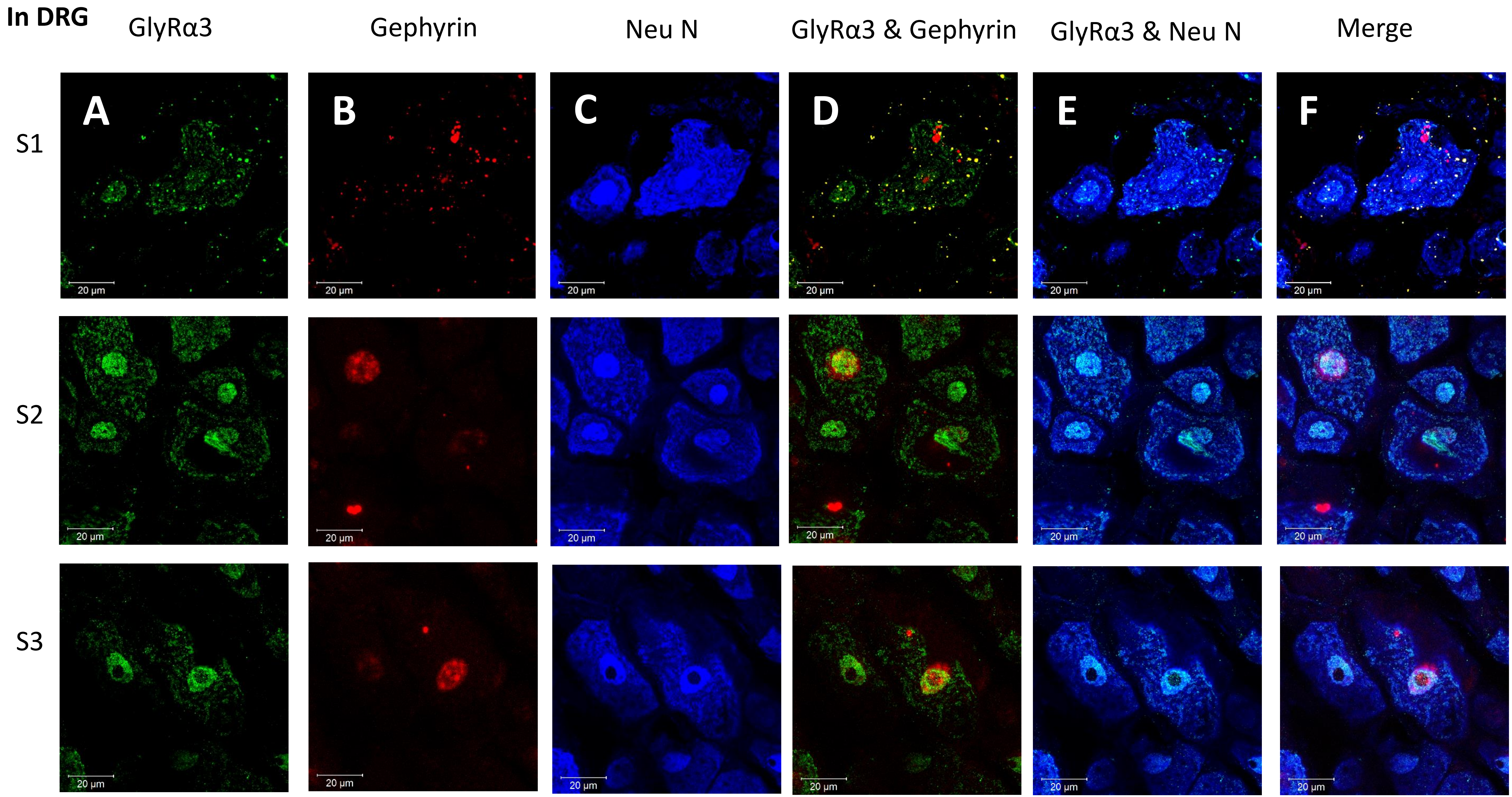
In DRG
GlyRα3
Gephyrin
Neu N
GlyRα3 & Gephyrin
GlyRα3 & Neu N
Merge
A
B
C
D
E
F
S1
S2
S3
20 μm

In DRG

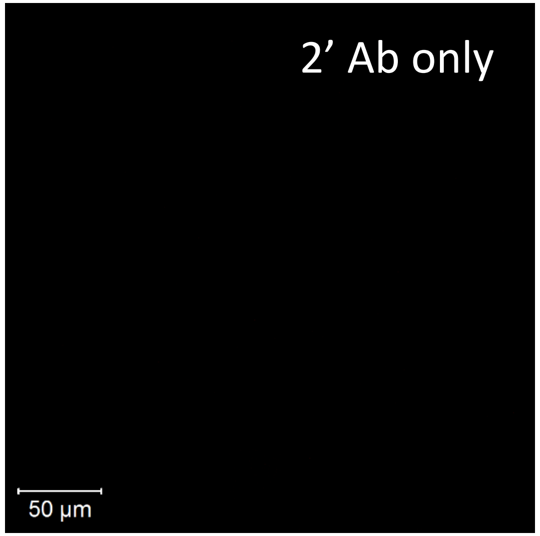

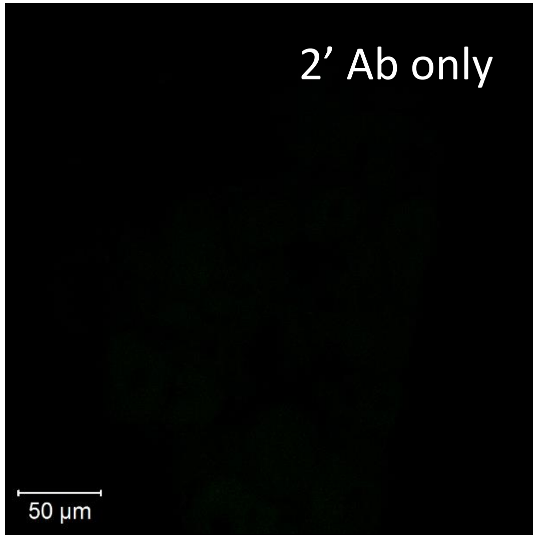

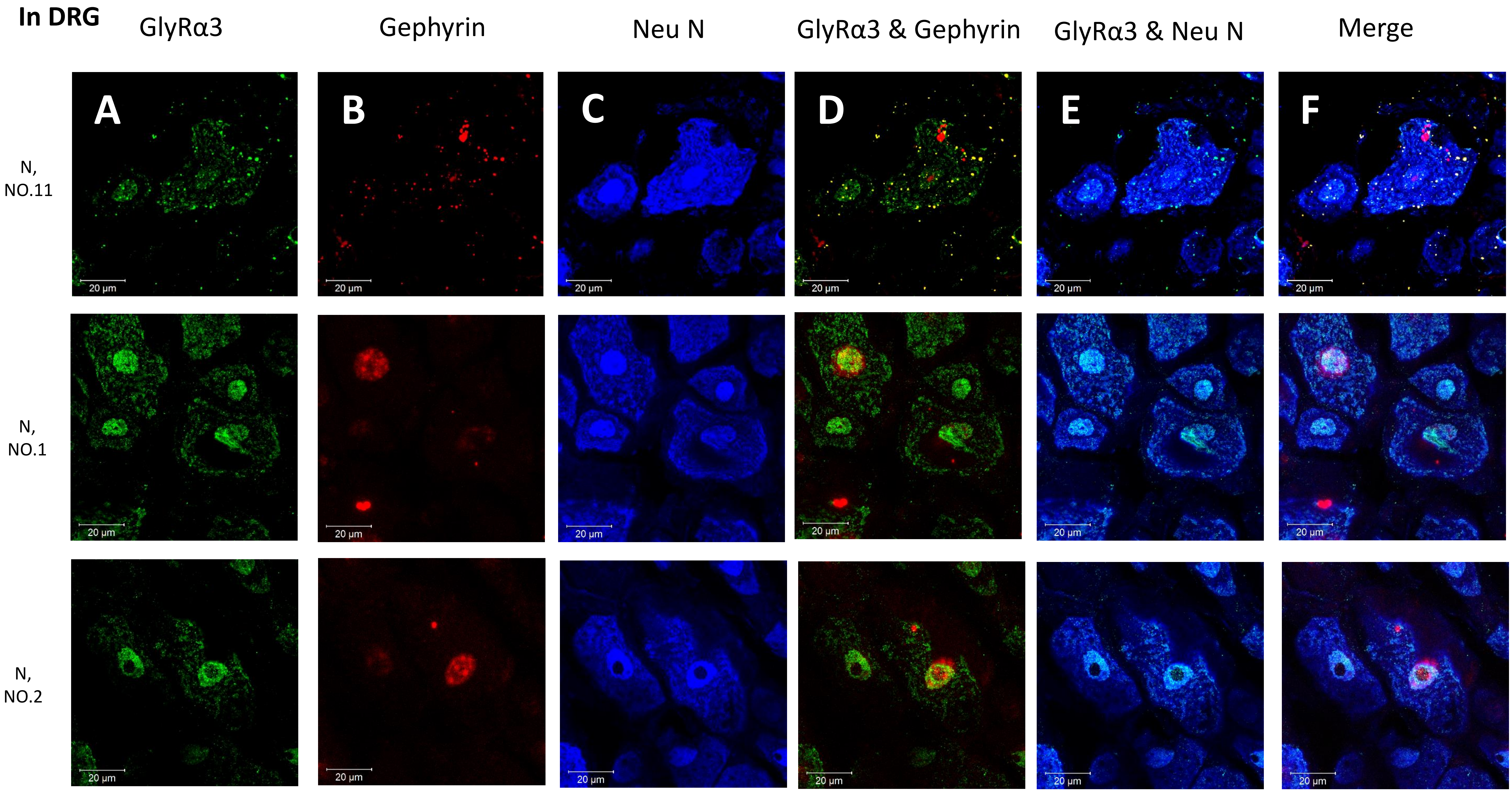
In DRG
GlyRα3
Gephyrin
Neu N
GlyRα3 & Gephyrin
GlyRα3 & Neu N
Merge
A
B
C
D
E
F
N, NO.11
N, NO.1
N, NO.2
20 μm

1˚Ab(host)	2˚Ab(host)
X	Cy5 (donkey anti-mouse)

1˚Ab(host)	2˚Ab(host)
X	Alexa Fluor® 488 (Dondey anti-goat)

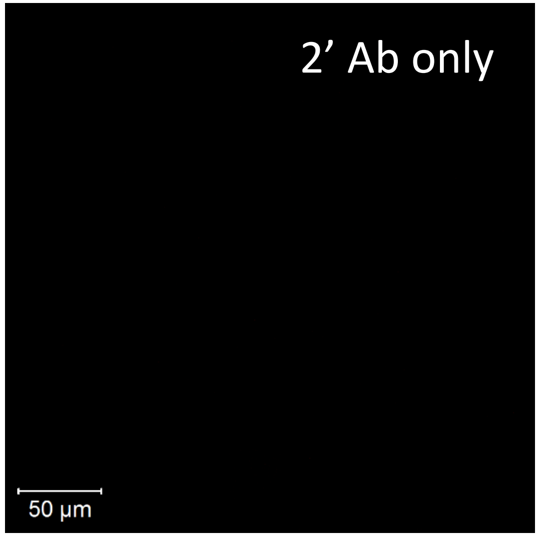

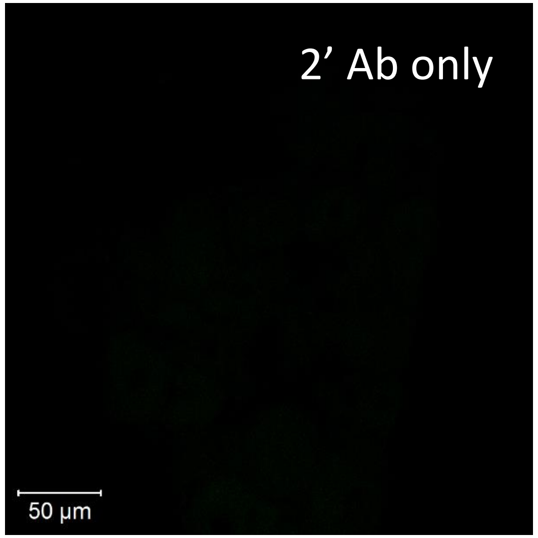

In DRG

1°Ab(host)	2°Ab(host)
GlyRα3 (goat)	Alexa Fluor® 488 (Dondey anti-goat)

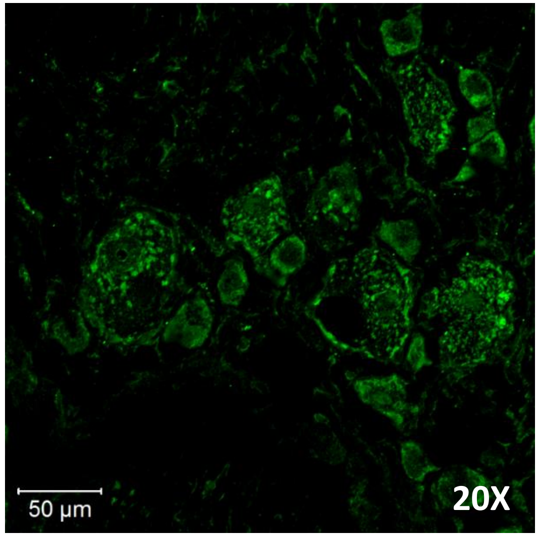

檔案資料夾：20170818 IF_DRG
抗體專一性測試

1°Ab(host)	2°Ab(host)
GlyRα3 (goat)	X

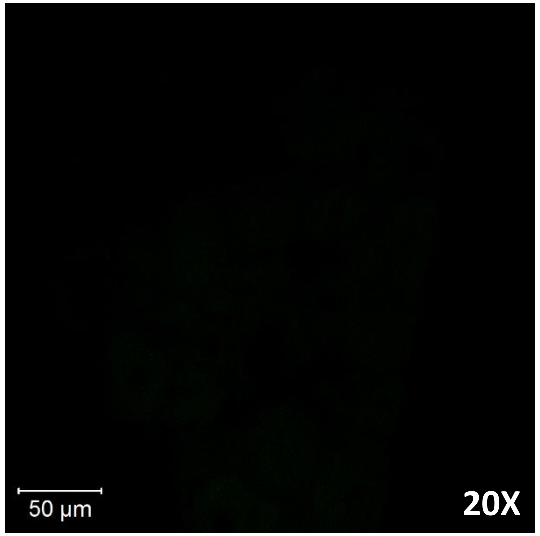

檔案資料夾：20170818 IF_DRG
抗體專一性測試

1°Ab(host)	2°Ab(host)
X	Alexa Fluor® 488 (Dondey anti-goat)

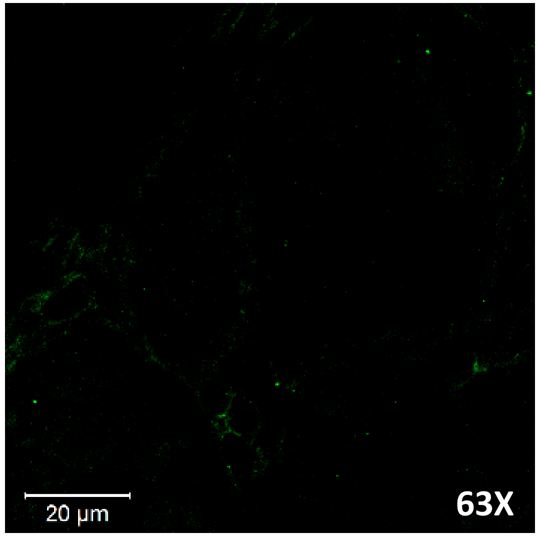

檔案資料夾：20170802 IF_DRG
SC_抗體專一性測試

In DRG

1˚Ab(host)	2˚Ab(host)
Gephyrin (mouse)	Cy5 (donkey anti-mouse)

NO.1

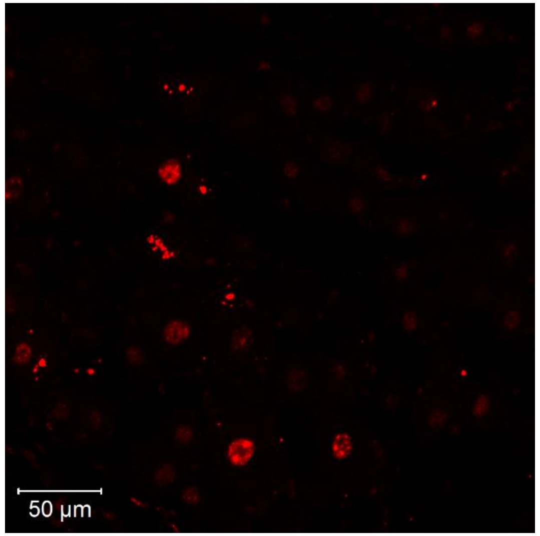

NO.2

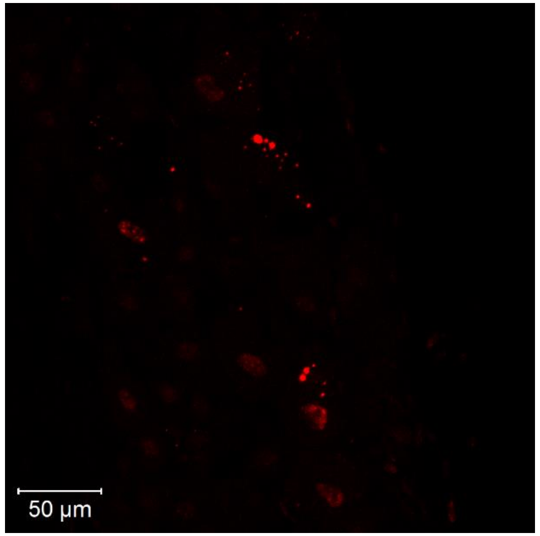

1˚Ab(host)	2˚Ab(host)
X	Cy5 (donkey anti-mouse)

NO.1

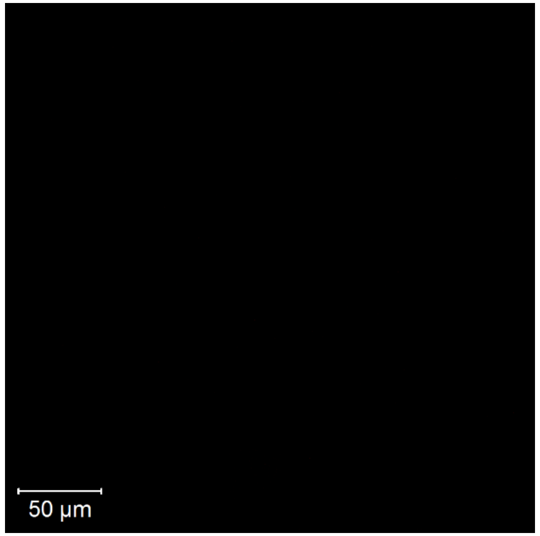

NO.2

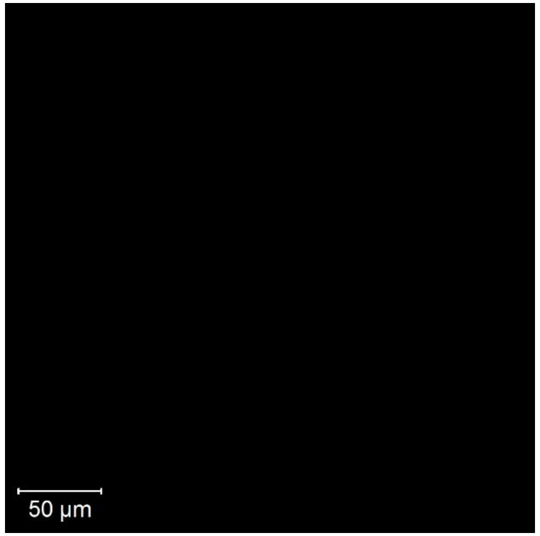

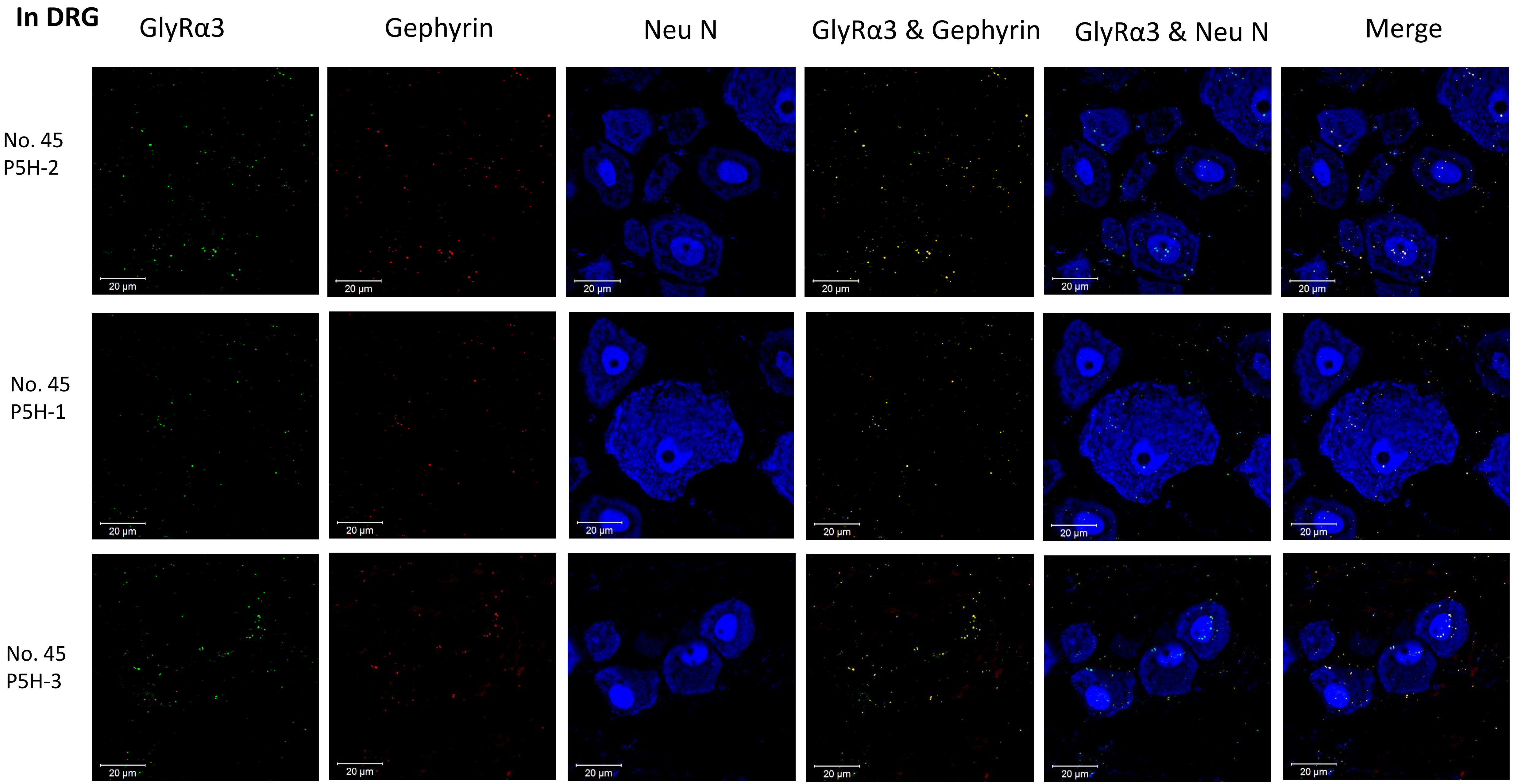

In DRG
GlyRα3
Gephyrin
Neu N
GlyRα3 & Gephyrin
GlyRα3 & Neu N
Merge
No. 45 P5H-2
No. 45 P5H-1
No. 45 P5H-3
20 μm

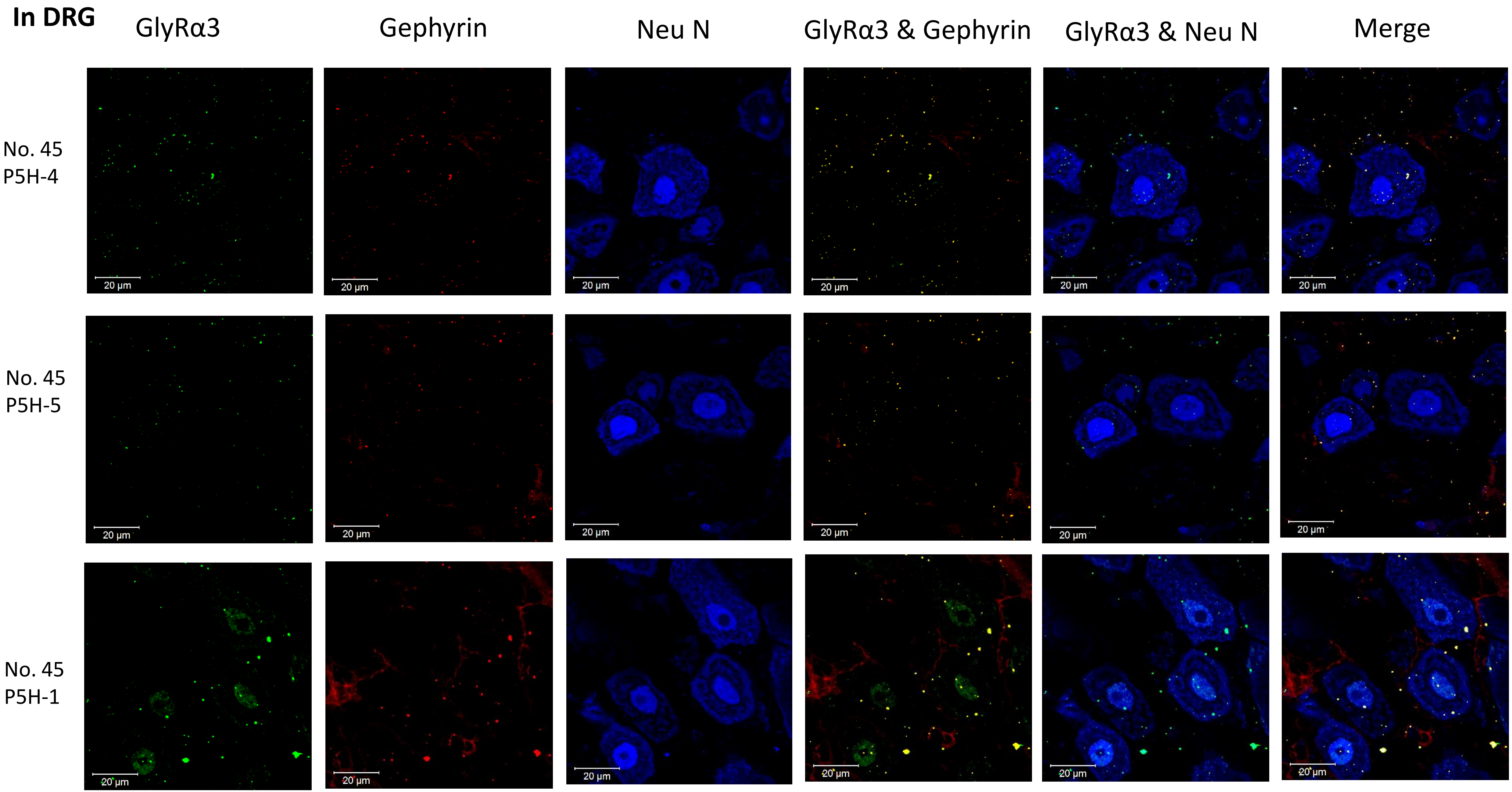

In DRG
GlyRα3
Gephyrin
Neu N
GlyRα3 & Gephyrin
GlyRα3 & Neu N
Merge
No. 45
P5H-4
No. 45
P5H-5
No. 45
P5H-1
20 μm

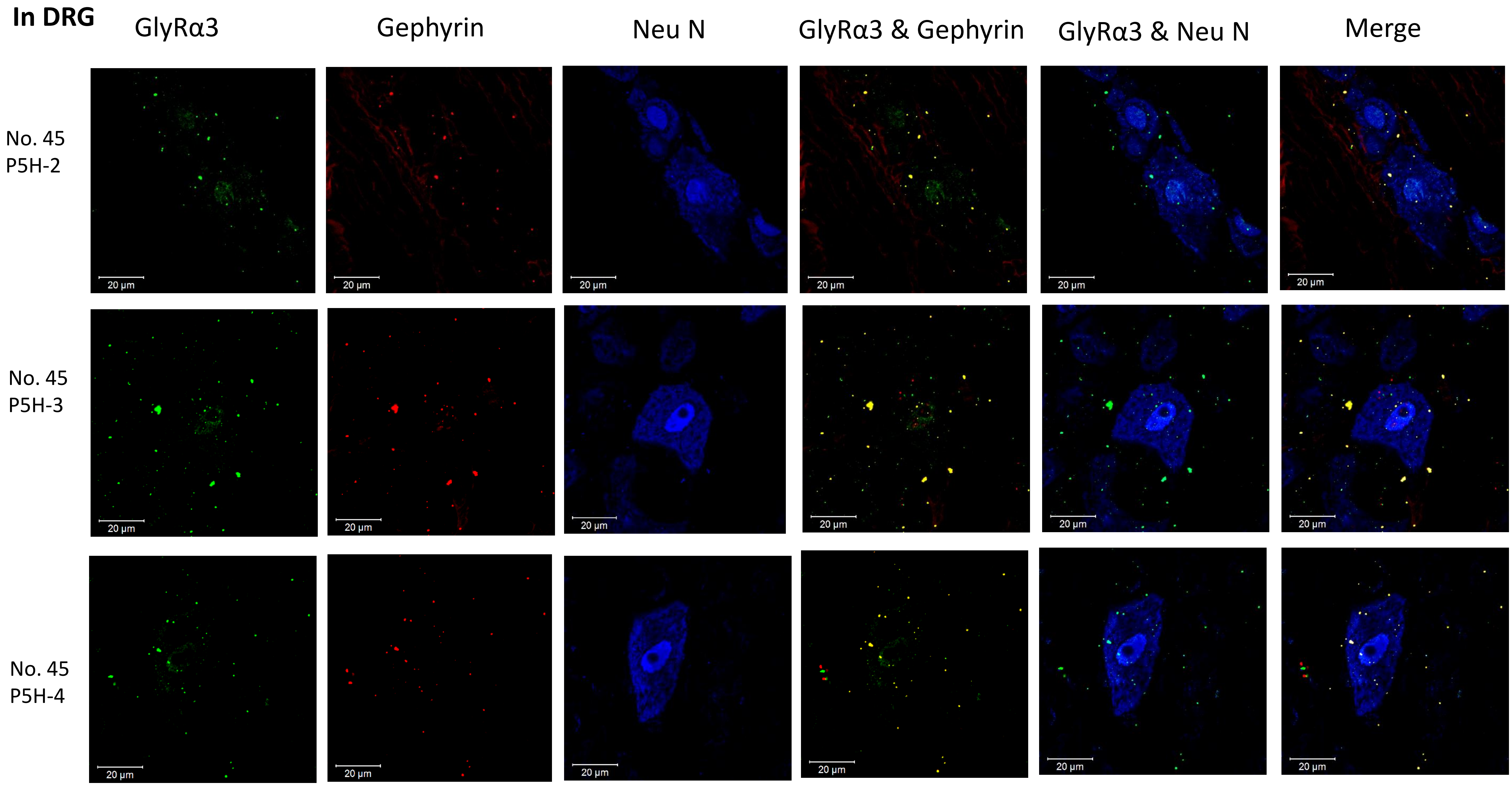
In DRG
GlyRα3
Gephyrin
Neu N
GlyRα3 & Gephyrin
GlyRα3 & Neu N
Merge
No. 45
P5H-2
No. 45
P5H-3
No. 45
P5H-4
20 μm

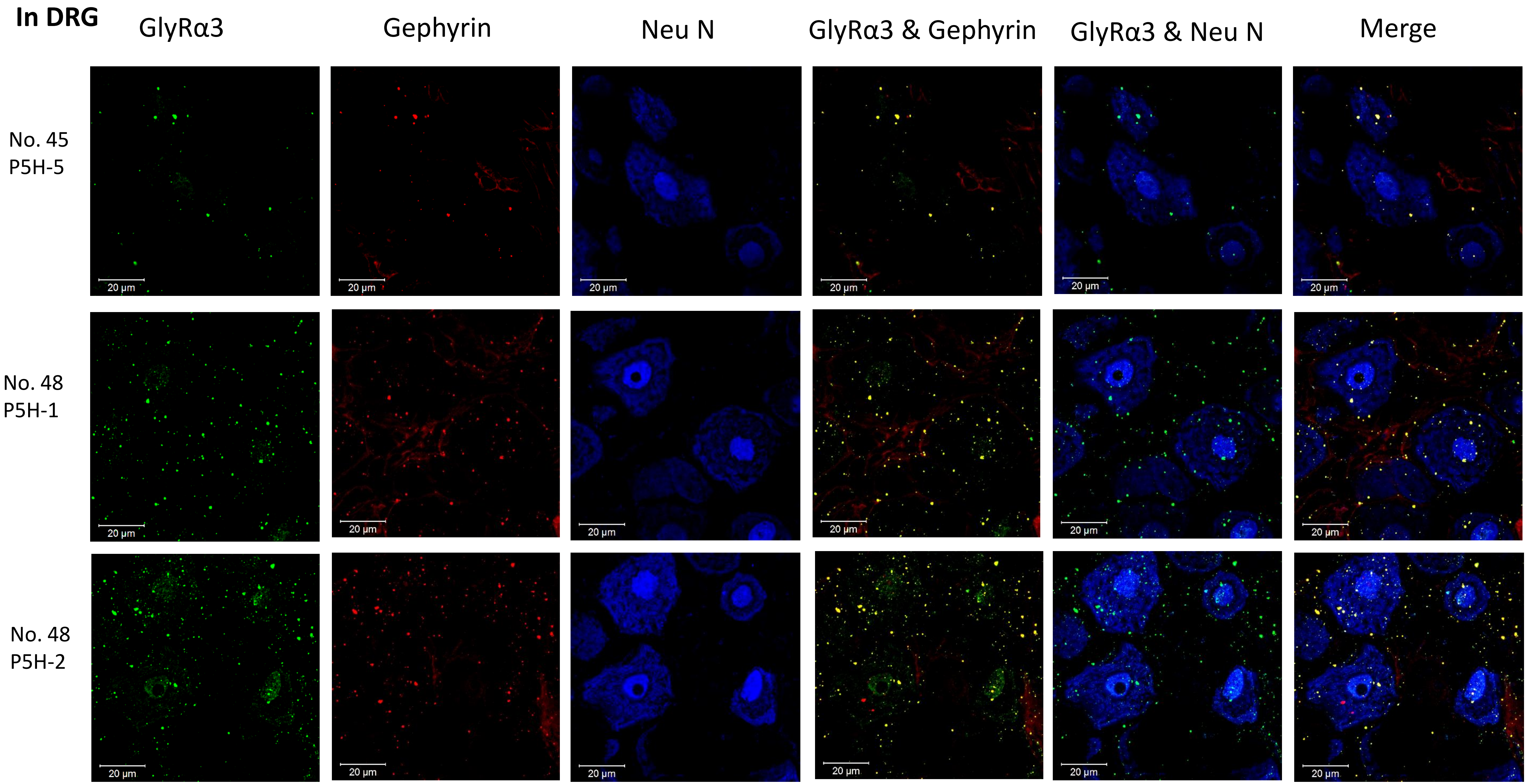
In DRG
GlyRα3
Gephyrin
Neu N
GlyRα3 & Gephyrin
GlyRα3 & Neu N
Merge
No. 45
P5H-5
No. 48
P5H-1
No. 48
P5H-2
20 μm

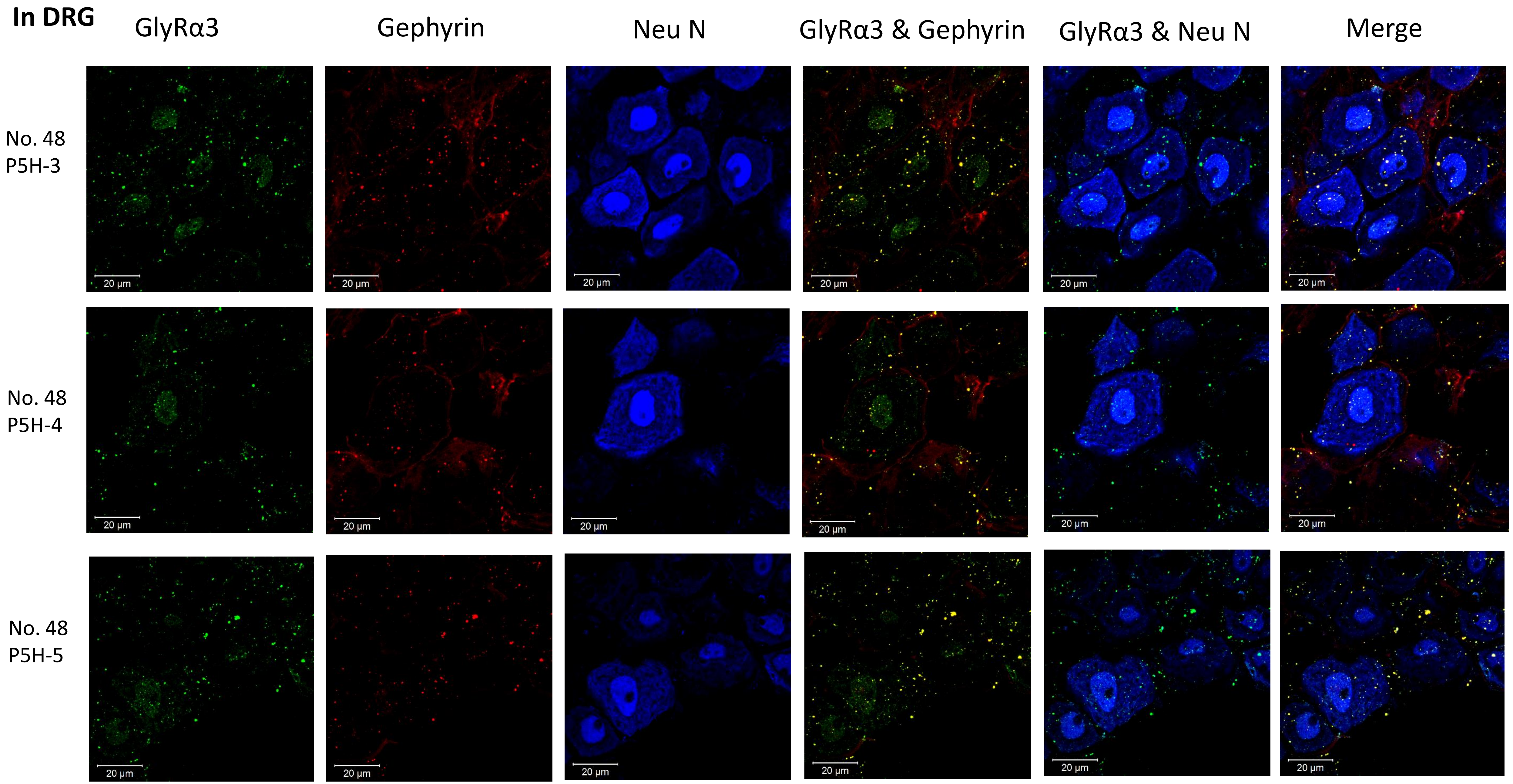

In DRG
GlyRα3
Gephyrin
Neu N
GlyRα3 & Gephyrin
GlyRα3 & Neu N
Merge
No. 48
P5H-3
No. 48
P5H-4
No. 48
P5H-5
20 μm

In DRG

GlyRα3 | Gephyrin | Neu N | GlyRα3 & Gephyrin | GlyRα3 & Neu N | Merge
